# Artificial intelligence technologies and compassion in healthcare: A systematic scoping review

**DOI:** 10.3389/fpsyg.2022.971044

**Published:** 2023-01-17

**Authors:** Elizabeth Morrow, Teodor Zidaru, Fiona Ross, Cindy Mason, Kunal D. Patel, Melissa Ream, Rich Stockley

**Affiliations:** ^1^Research Support Northern Ireland, Downpatrick, United Kingdom; ^2^Department of Anthropology, London School of Economics and Political Sciences, London, United Kingdom; ^3^Faculty of Health, Science, Social Care and Education, Kingston University London, London, United Kingdom; ^4^Artificial Intelligence Researcher (Independent), Palo Alto, CA, United States; ^5^Iheed, Dublin, Ireland; ^6^Kent Surrey Sussex Academic Health Science Network (AHSN) and the National AHSN Network Artificial Intelligence (AI) Initiative, Surrey, United Kingdom; ^7^Head of Research and Engagement, Surrey Heartlands Health and Care Partnership, Surrey, United Kingdom

**Keywords:** artificial intelligence (AI), compassion, compassionate healthcare, empathy, healthcare technology

## Abstract

**Background:**

Advances in artificial intelligence (AI) technologies, together with the availability of big data in society, creates uncertainties about how these developments will affect healthcare systems worldwide. Compassion is essential for high-quality healthcare and research shows how prosocial caring behaviors benefit human health and societies. However, the possible association between AI technologies and compassion is under conceptualized and underexplored.

**Objectives:**

The aim of this scoping review is to provide a comprehensive depth and a balanced perspective of the emerging topic of AI technologies and compassion, to inform future research and practice. The review questions were: How is compassion discussed in relation to AI technologies in healthcare? How are AI technologies being used to enhance compassion in healthcare? What are the gaps in current knowledge and unexplored potential? What are the key areas where AI technologies could support compassion in healthcare?

**Materials and methods:**

A systematic scoping review following five steps of Joanna Briggs Institute methodology. Presentation of the scoping review conforms with PRISMA-ScR (Preferred Reporting Items for Systematic reviews and Meta-Analyses extension for Scoping Reviews). Eligibility criteria were defined according to 3 concept constructs (AI technologies, compassion, healthcare) developed from the literature and informed by medical subject headings (MeSH) and key words for the electronic searches. Sources of evidence were Web of Science and PubMed databases, articles published in English language 2011–2022. Articles were screened by title/abstract using inclusion/exclusion criteria. Data extracted (author, date of publication, type of article, aim/context of healthcare, key relevant findings, country) was charted using data tables. Thematic analysis used an inductive-deductive approach to generate code categories from the review questions and the data. A multidisciplinary team assessed themes for resonance and relevance to research and practice.

**Results:**

Searches identified 3,124 articles. A total of 197 were included after screening. The number of articles has increased over 10 years (2011, *n* = 1 to 2021, *n* = 47 and from Jan–Aug 2022 *n* = 35 articles). Overarching themes related to the review questions were: (1) *Developments and debates* (7 themes) Concerns about AI ethics, healthcare jobs, and loss of empathy; Human-centered design of AI technologies for healthcare; Optimistic speculation AI technologies will address care gaps; Interrogation of what it means to be human and to care; Recognition of future potential for patient monitoring, virtual proximity, and access to healthcare; Calls for curricula development and healthcare professional education; Implementation of AI applications to enhance health and wellbeing of the healthcare workforce. (2) *How AI technologies enhance compassion* (10 themes) Empathetic awareness; Empathetic response and relational behavior; Communication skills; Health coaching; Therapeutic interventions; Moral development learning; Clinical knowledge and clinical assessment; Healthcare quality assessment; Therapeutic bond and therapeutic alliance; Providing health information and advice. (3) *Gaps in knowledge* (4 themes) Educational effectiveness of AI-assisted learning; Patient diversity and AI technologies; Implementation of AI technologies in education and practice settings; Safety and clinical effectiveness of AI technologies. (4) *Key areas for development* (3 themes) Enriching education, learning and clinical practice; Extending healing spaces; Enhancing healing relationships.

**Conclusion:**

There is an association between AI technologies and compassion in healthcare and interest in this association has grown internationally over the last decade. In a range of healthcare contexts, AI technologies are being used to enhance empathetic awareness; empathetic response and relational behavior; communication skills; health coaching; therapeutic interventions; moral development learning; clinical knowledge and clinical assessment; healthcare quality assessment; therapeutic bond and therapeutic alliance; and to provide health information and advice. The findings inform a reconceptualization of compassion as a *human-AI system of intelligent caring* comprising six elements: (1) Awareness of suffering (e.g., pain, distress, risk, disadvantage); (2) Understanding the suffering (significance, context, rights, responsibilities etc.); (3) Connecting with the suffering (e.g., verbal, physical, signs and symbols); (4) Making a judgment about the suffering (the need to act); (5) Responding with an intention to alleviate the suffering; (6) Attention to the effect and outcomes of the response. These elements can operate at an individual (human or machine) and collective systems level (healthcare organizations or systems) as a cyclical system to alleviate different types of suffering. New and novel approaches to human-AI intelligent caring could enrich education, learning, and clinical practice; extend healing spaces; and enhance healing relationships.

**Implications:**

In a complex adaptive system such as healthcare, human-AI intelligent caring will need to be implemented, not as an ideology, but through strategic choices, incentives, regulation, professional education, and training, as well as through joined up thinking about human-AI intelligent caring. Research funders can encourage research and development into the topic of AI technologies and compassion as a system of human-AI intelligent caring. Educators, technologists, and health professionals can inform themselves about the system of human-AI intelligent caring.

## Highlights

- Artificial intelligence (AI) refers to computer systems that are designed to think or act like humans (human approach) and systems that think or act rationally (rational approach). However, current thinking about AI falls short of understanding the underlying motivational systems of thinking and acting like a human (e.g., compassion) or the influence of such motivational systems on complex societal systems (e.g., healthcare).
- Exploration of the associations between AI technologies and compassion have been hindered by two widely held assumptions (1) that compassion is a felt emotion in the body produced through relationships and social dynamics, and (2) that technologies are objective and incapable of compassion (again an assumption based on a view that compassion only involves felt emotion). Although it is debated whether AI can feel or express genuine empathy, compassion is different because it is a system.
- The literature shows that AI technologies can be (a) individually programmed (i.e., “build compassion in”) to mimic elements of human compassion (e.g., emotion detection, affective response, empathetic display, socio-cultural intelligence) to various degrees of authenticity and success, and (b) be used collectively within a system of healthcare to enhance compassion (e.g., increasing empathetic awareness, assessing needs in high-risk patient groups, understanding the person), (i.e., “use it for compassion”).
- Compassion can be conceptualized as a *human-AI system of intelligent caring* comprising six elements: (1) Awareness of suffering (e.g., pain, distress, risk, disadvantage); (2) Understanding the suffering (significance, context, rights, responsibilities etc.); (3) Connecting with the suffering (e.g., verbal, physical, signs and symbols); (4) Making a judgment about the suffering (the need to act); (5) Responding with an intention to alleviate the suffering; (6) Attention to the effect and outcomes of the response. Future research into these elements could develop new and novel approaches to human-AI intelligent caring.

## 1. Introduction

Artificial intelligence (AI) refers to computer systems that are designed to think or act like humans (human approach) and systems that think or act rationally (rational approach) (Russell and Novig, 2020). This article uses a systematic scoping review of the literature to explore the possible association between AI technologies and compassion in healthcare. This topic relates to current debates about the way AI might be perceived or imagined to be caring (De Togni et al., 2021) or compassionate (Day et al., 2021).

Exploring the possible association between AI and compassion is important because AI mediates every area of healthcare systems (e.g., complex systems involving purchasers, providers, payers, patients, and so on) by powering search engines, analysing data and making recommendations (Bajwa et al., 2021), as well as through clinical and health-related applications (Davenport and Kalakota, 2019). AI can be incredibly powerful for processing (e.g., using pattern recognition or predictive capabilities) “big data,” which refers to the masses of data that are increasingly readily available in society through digital devices (Topol, 2019). Machine learning is the most common form of AI and largely relies on supervised learning, when computers are trained with labels decided by humans. Deep learning and adversarial learning involve training on unlabeled data to reveal underlying patterns (e.g., algorithms are used to find clusters or variances in data) (see^1^). However, current thinking about AI falls short of understanding the underlying motivational systems of thinking and acting like a human (e.g., compassion) or the influence of such motivational systems on complex societal systems (e.g., healthcare).

Yet current research shows that AI technologies (i.e., AI-driven machines, devices, programs, or applications) influence not only how humans think and act but how healthcare professionals work and learn (Bin Kamarudin and Zary, 2019) (“healthcare professionals” is used here to mean the wide range of trained professionals that deliver clinical treatments and care e.g., medical, surgical, nursing, professions allied to medicine, mental health professionals, and so on, rather than the broader health professions, general managers, administrative staff etc.). For example, by informing more accurate imaging and diagnosis (Nichols et al., 2019), improving the efficiency of clinical screening (Grzybowski et al., 2020), enabling personalized medicine (Schork, 2019), and precision medicine that is tailored to individual patient needs (Mesko, 2017; Chang, 2020). Within healthcare organizations, AI may support improved productivity, workload, performance, teamwork, and satisfaction (Hazarika, 2020; Morley et al., 2020). Patients will increasingly experience new e-health (electronic health) applications in clinical settings (Lupton, 2017), in their own homes and mhealth (mobile health) applications in their lives (Torous et al., 2018). So, to explore how AI technologies might support compassion in healthcare systems it is important to look more deeply at what compassion is.

Compassion has been described as a sensitivity to suffering in self and others, with a commitment to try and alleviate and prevent it (Gilbert, 2014). It is perceived to be an evolutionary survival feature of a social species, which promotes helpful caring behaviors in an interconnected field of social relations, steered by ethical values and social norms (Goetz et al., 2010; Gilbert, 2019). Compassionate behavior is modeled and learnt through human interactions, such as parenting and teaching (Goetz et al., 2010). Compassion research demonstrates how the psychology of compassion in the mind (experiencing or witnessing helpful interactions) (Walter et al., 2015) affects the body, improves human health (Kim et al., 2009), and benefits societies (Seppälä et al., 2017). Yet, compassion is under conceptualized and underexplored in relation to AI technologies (Bloom, 2016; Kerasidou, 2020) or the question of how AI technologies might be used to generate or enhance compassion (Day et al., 2021). Exploration of the associations between AI technologies and compassion have been hindered by two widely held assumptions (1) that compassion is a felt emotion in the body produced through relationships and social dynamics and (2) that technologies are objective and incapable of compassion (White and Katsuno, 2019) (again an assumption based on a view that compassion only involves felt emotion). Although it is debated whether AI can feel or express genuine empathy (Montemayor et al., 2021), compassion is different because it is a motivational caring system (Gilbert, 2019).

In healthcare contexts there is considerable interest in compassion for ethical and clinical reasons (Fotaki, 2015; Papadopoulos and Ali, 2016). Compassion is described as a “medical virtue” (De Bakey, 2018), a “virtuous response” (Sinclair et al., 2016a,b) or “intelligent kindness” (Gallagher and Wainwright, 2005). Compassion is an expectation of recruitment to healthcare jobs (Straughair, 2019); a component of ethical professional practice (Flores and Brown, 2018); an indicator of healthcare quality (Sinclair et al., 2017; Durkin et al., 2018; Clavelle et al., 2019; Thomas and Hazif-Thomas, 2020; Baguley et al., 2022); and a dynamic interactional experience that includes motivation, capacity, and connection (Uygur et al., 2019). Compassionate caregiving has been described as involving meaningful actions to alleviate suffering and meet individual needs and prevent further suffering (Durkin et al., 2021). Compassionate behaviors (Straughair, 2019) are taught through pedagogy (Hendry, 2019), learning objectives (Lown, 2016; Sinclair et al., 2021; Wang et al., 2022) assessment (Lown et al., 2016), and skills sets such as reflective listening (Braillon and Taiebi, 2020; Su et al., 2021). Healthcare research has examined compassion from the perspective of: the predictors of compassion in healthcare professionals (Fernando and Consedine, 2014; Bleiker et al., 2020; Pavlova et al., 2022); how care environment and organizational culture affect compassion (Casagrande, 2016; Ali and Terry, 2017; Dev et al., 2019; Tehranineshat et al., 2019; Wiljer et al., 2019; Ali et al., 2022); compassion-maintaining strategies and interventions (Blomberg et al., 2016; Terry et al., 2017; Flores and Brown, 2018; Baguley et al., 2020; Hopkins et al., 2021; Malenfant et al., 2022); compassionate leadership (Dewar and Cook, 2014; de Zulueta, 2015; Lown et al., 2019; West et al., 2020); and regulation of compassionate caregiving (Harris et al., 2019; Pedersen and Roelsgaard Obling, 2019). Culturally and critically informed perspectives of compassion highlight that different societies, professional groups, cultures, and generations hold different expectations and views about compassion (Koopmann-Holm and Tsai, 2017; Sundus et al., 2020) which change over time (Salvador Zaragozá et al., 2021). Compassion has been described as a lens for critically considering the cultural and social significance of AI technologies and the different ways that such technologies may serve or disserve the societies that created them (White and Katsuno, 2019) including how technologies affect their users (Day et al., 2021).

In recent years some AI technologists and researchers have become interested in how AI technologies might demonstrate caring or be caring (De Togni et al., 2021). Artificial empathy refers to the coding of empathy into machines (Dial, 2018) whereby emotion recognition and display technologies are designed to sense and/or show a sense of empathy in their users e.g., giving life-like virtual agents the capabilities to mimic user’s facial expressions. However, technologies that appear to be empathetic may not necessarily be genuine or authentic empathy (Montemayor et al., 2021). A machine capable of artificial compassion requires more than emotion recognition and expression (Mason, 2015). Artificial compassion refers to the steps that technologists may take to intentionally design adaptive responsiveness into technologies (Critchley, 2015). For example, building cognitive architecture (a control loop that the computer runs through) that Sense-{Think + Feel}-React (Mason, 2015, 2021, 2023). In this type of computing the ability to “think” and “feel” are made possible by connecting to external reference points such as information in the cloud, or other agents, to develop a form of socio-cultural intelligence (Mason, 2021). Not all technologies need these types of “in-built” compassion in their programming but these developments in AI systems will influence societal systems.

This article draws on different fields of systems thinking (Dori and Sillitto, 2017) to explore the associations between the types of systems involved. That is, AI technologies as computational systems (e.g., machine learning, deep-learning, algorithms, network systems etc.); compassion as a motivational caring system (Motivational Systems Theory) (Ford, 1992); and healthcare systems as complex adaptive systems (Complex Adaptive Systems Theory) (Lansing, 2003; Levin, 2003). Motivation is thought to be at the heart of many of society’s and healthcare’s most pervasive and enduring problems (Ford, 1992) (e.g., the “care gap”). These perspectives enabled this review to explore issues about the way technologies are imagined and used, and their capabilities to alleviate suffering through compassion.

### 1.1. Rationale

Advances in AI technologies and research on compassion have seen significant development and progress in recent years. However, understandings about possible associations between AI technologies and compassion are emergent and under conceptualized. It is unclear what type of AI technologies can be designed and used to enhance compassion in healthcare.

Understanding any associations between AI technologies and compassion is important in a western healthcare context that is characterized by numerous politicized issues about supply-demand-challenges in healthcare associated with a clinically complex aging population (Tiersen et al., 2021), historical under resourcing in some health services, and the COVID-19 crisis (Pagliari, 2021). These challenges have been described as a growing “care gap” (Davendralingam et al., 2017). There is also an apparent deficit or lack of compassion in healthcare systems: notions of the “compassion gap” (Trzeciak et al., 2017), or “crisis in caring” with suggestions there is “empathy erosion” or an “empathy deficit” (Stenhouse et al., 2016). The Francis Report (Mid Staffordshire NHS Foundation Trust Public Inquiry, 2013) revealed sub-standard patient care and increased mortality rates in UK hospitals to show the devastating effects of practicing medicine without compassion (Gray and Cox, 2015). Consequently, multiple “compassion cultivation” programs and initiatives such as empathy training have been implemented in health services and staff training (Davendralingam et al., 2017). Other related issues include “compassion fatigue” (Sheppard, 2015; Figley and Regan Figley, 2017), staff resilience and staff burnout (Stevenson et al., 2022), and the “pure hard slog” of caregiving roles (Bogossian et al., 2014). Issues about the human cost of emotional labor (Larson and Yao, 2005) are reflected in “compassionomics”: The study of the effects of compassionate healthcare for patients, healthcare systems, payers, and providers (Trzeciak et al., 2017). This context also includes issues about the prevalence of workplace discrimination and violence in healthcare (Greinacher et al., 2022), intention to leave (Greinacher et al., 2022), COVID-19 related “compassion collapse” (Hagman et al., 2022), as well as staff experiences of “compassion satisfaction” (enjoyment, reward, and passion for work) (Okoli et al., 2020; Baqeas et al., 2021; Qu et al., 2022; Unjai et al., 2022). Other research has investigated “compassion inequalities,” which refers to differentials in patient treatment and care associated with stigmatized health conditions such as opioid use disorder (Singh et al., 2021). These issues set an important but complex context for exploring how AI technologies might be used to address some of the real-world “caring problems” in healthcare systems.

Current conceptualizations of compassion are limited by the fact that they do not consider the possibility of AI technologies as tools for compassion (except for artificial compassion, Mason, 2021, 2023). Compassion science mainly focuses on the bodily (psychological and neurobiological) and behavioral elements of compassion (Kim et al., 2020; Goldberg, 2020) and the effects of oxytocin in the body (Brown and Brown, 2015; Palgi et al., 2016; Seppälä et al., 2017). There is growing evidence about self-compassion and compassionate touch interventions (Bond, 2002; Field, 2014; Serpa et al., 2021), self-care interventions (Ehret et al., 2015; Friis et al., 2016; Brown et al., 2020), professionals’ self-care and self-compassion and compassion for others (Mills et al., 2017); and resilience in caring roles (Bleazard, 2020; Baqeas et al., 2021). Compassion is often used interchangeably with the notion of empathy (Håkansson Eklund and Summer Meranius, 2021); previously defined as a person’s ability to sense another’s thoughts, feelings, and experiences, to share the other’s emotional experience, and to react to the observed experiences of another person (Wieseke et al., 2012). However, compassion is different to empathy. Compassion refers to not only a sensitivity to suffering, but the commitment to try to alleviate and prevent it, i.e., a caring motivational system (Gilbert, 2019).

Understanding any potential of AI technologies to enhance compassion could help to respond to many different concerns about modern technologies in healthcare. Issues that include the safe and ethical use of information and communication technologies as clinical devices (Lupton, 2017), “data entry burden”(Dragano and Lunau, 2020), and “digital tick-boxing” associated with electronic health records (Collier, 2017); information overload and “doctor as machine” (Padmanabhan, 2020); screen fatigue associated with telemedicine and device use (Alameddine et al., 2019); “digital distraction,” frequent prompts and interruptions to care that affect service safety and quality; “technostress” (La Torre et al., 2020) when technologies don’t meet expectations creating negative feelings or behaviors; “disinhibition effect” (Terry and Cain, 2016) associated with online settings that can include “cyberbullying” (Hutson et al., 2018); “digital exclusion” and “digital inequalities” (Crotty and Somai, 2022); maintaining human connection with mediated communication (Nieto et al., 2021); as well as concerns about the safe, ethical and fair uses of AI technologies (Buolamwini, 2017; Figueroa et al., 2021; Martinez-Martin et al., 2021; Oszutowska-Mazurek et al., 2021; Suresh and Guttag, 2021; Tschandl, 2021; Schmidt et al., 2022).

### 1.2. Objectives

The objective of this scoping review was to provide a comprehensive depth and a balanced perspective of the emerging topic of AI technologies and compassion to inform future research and practice.

### 1.3. Approach

The approach was to undertake a scoping review of the topic using a recognized framework and process. We used the approach originally proposed by Arksey and O’Malley (2005), further enhanced by the work of Levac et al. (2010) and consolidated in the Joanna Brigs Institute (JBI) approach to the conduct of scoping reviews (Peters et al., 2020). Presentation of the scoping review conforms with PRISMA-ScR (Preferred Reporting Items for Systematic reviews and Meta-Analyses extension for Scoping Reviews) guidelines and 20 essential item checklist (Tricco et al., 2018) from the EQUATOR (Enhancing the QUAlity and Transparency Of health Research) Network.

Scoping reviews are useful for examining emerging evidence when it is still unclear what other, more specific questions can be posed for evidence syntheses and valuably addressed (Mays et al., 2001). Unlike a systematic review, scoping reviews do not tend to produce and report results that have been synthesized from multiple evidence sources following a formal process of methodological appraisal to judge the quality of the evidence (Peters et al., 2020). Rather, scoping reviews follow a systematic approach to map evidence on a topic and identify main concepts, theories, sources, and knowledge gaps (Tricco et al., 2018).

Five main stages of the review process (Arksey and O’Malley, 2005) were:

(1) identifying the research question.
(2) identifying relevant studies.
(3) study selection.
(4) charting the data.
(5) collating, summarizing, and reporting the results.

Each stage was informed by the team’s multidisciplinary expertise and understanding from fields of nursing, medicine, anthropology, health service research, AI strategy, and AI technology design. Our working methods were to use online meetings for discussions (*via* Microsoft Teams) supported by sharing files, articles, and comments (using Miro whiteboard and file share software).

Four review questions were developed to reflect the aims:

1. How is compassion discussed in relation to AI technologies in healthcare? For example, different schools of thought, controversies, or perspectives.
2. How are AI technologies being used to enhance compassion in healthcare? For example, professional practice, education and learning, clinical care, or health care delivery or outcomes.
3. What are the gaps in current knowledge and unexplored potential? For example, are there uncertainties, problematic concepts, or a lack of empirical research.
4. What are the key areas where AI technologies could support compassion in healthcare? For example, suggestions or claims for how AI technologies may support compassion in healthcare in the future.

### 1.4. Definitions and scope

To explore possible associations between AI technologies and compassion in healthcare a broad scope of the review was defined according to three concept constructs, explained below.

#### 1.4.1. AI technologies construct

A comprehensive list of key terms for the searches was generated by drawing on existing definitions of AI (Russell and Novig, 2020), subject indexing for artificial intelligence (National Library of Medicine), knowledge of the team (CM and MR), search terms used in a previous review of AI technologies in mental health (Zidaru et al., 2021) and digital health interventions (Boucher et al., 2021; Table 1). The terms did not include issues or factors relating to digital health (Lupton, 2017), patient consent, data sharing, electronic health records see (de Zulueta, 2021), remote healthcare delivery, internet-based modes of health information delivery, digital health platforms, web-based health interventions, online health clinics, virtual visits/care or telemedicine, or telehealth.

**TABLE 1:** Key search terms.

AI technologies construct	Compassion construct	Healthcare construct
Affective computing	Compassion	Health care
Artificial intelligence	Empathy	Health-care
Automation	Self-compassion	Healthcare
Bioinformatics	Compassion fatigue	
Chatbot		
Computer-assisted		
Data mining		
Decision support systems		
Deep learning		
Digital health		
eHealth/e-health		
electronic health		
Health app		
Human machine systems		
Information systems		
Machine learning		
Medical informatics		
mHealth		
Neural networks, computer		
Natural language processing		
Robotics		
Smartphone		
Sentiment analysis		
Virtual reality		
Wearable		

Table 1 shows the key search terms that were developed for each construct and used in the electronic searches.

#### 1.4.2. Compassion construct

As the aim of this review was to focus on the concept of “compassion” in relation to AI technologies the compassion construct for the searches used key terms that are most associated with compassion in the literature (these are “compassion” and “empathy”). Medical subject classification terms were not available for the term “compassion” (MeSH index compassion under Empathy), so key terms were identified from the literature on compassion (previously described in Section “1.1 Rationale”). We also decided to include terms for “self-compassion” and “compassion fatigue” to explore any association between AI technologies and these perspectives of compassion which are important in healthcare. It was also important to develop a working definition of compassion to support the screening and thematic analysis stages of the review, by drawing on existing literature on compassion. Although diverse perspectives and understandings of compassion exist, there is a degree of commonality around the notion of compassion as a prosocial/caring motivational system (Seppälä et al., 2017; Leffel et al., 2018; Gilbert, 2019). Expert consensus is that compassion has 5 component elements (Strauss et al., 2016): (1) recognizing suffering, (2) understanding the universality of suffering in human experience, (3) emotionally connecting with the person in distress, (4) tolerating uncomfortable feelings so that we are able to help, and (5) being motivated to act or acting to help/alleviate suffering (Gu et al., 2017). The present review drew on these understandings to create a working definition of compassion as involving:

(1) Awareness of suffering (e.g., pain, distress, risk, disadvantage).
(2) Understanding the suffering (significance, context, rights, responsibilities etc.).
(3) Connecting with the suffering (e.g., verbal, physical, signs and symbols).
(4) Making a judgment about the suffering (the need to act).
(5) Engaging in a behavior with the intention to alleviate the suffering.

Within this working definition, “suffering” is used to include notions of pain, distress, risk, and disadvantage in healthcare contexts (e.g., physical, or mental pain), as well as more broadly to include suffering associated with risks to health, hardship, social disadvantage (social determinants of health) (Braveman and Gottlieb, 2014), barriers to healthcare (Powell et al., 2016), and health inequalities (Scambler, 2012). This definition acknowledges that health and suffering extend beyond the provision of clinical treatment and clinical care, e.g., through actions to protect human rights, minimize risk to human lives, or promote health equality, for example.

#### 1.4.3. Healthcare construct

Healthcare was defined as a complex adaptive system: a complex dynamic network of interactions that might not always be predictable or perceivable to those within it (Cillers, 1998). It is adaptive, in that, the individual and collective behavior can alter and self-organize corresponding to internal or external micro-events or combined events (Lansing, 2003). Thus, the approach to the searches was to use broad key word terms (“health care,” “healthcare,” “health-care”) as a strategy to include articles relating to any groups of health professionals, different settings/fields (e.g., primary, acute, intermediate care, care homes, educational settings), and all groups of patients, carers. Different forms of the term “healthcare” are used in the literature and internationally, so variations of the term (i.e. single word, phrase, hyphenated) were used to ensure the searches could retrieve all relevant articles.

The AI technologies construct is defined in more specific terms, compared to the more general terms used to define the compassion and healthcare constructs. This is because the concept of compassion is itself complex, in that multiple understandings, perspectives and definitions of this term exist. Thus, in this review we needed to focus (specificity) on the concept of “compassion” to perform a meaningful exploration of how this concept is understood and used in relation to AI technologies. It was appropriate to use a general healthcare construct, a very broad definition, to set a wide context for the searches. Thus, the construct covers healthcare systems, health service organizations, as well as treatment and care provided by healthcare professionals.

## 2. Materials and methods

In accordance with PRISMA-ScR guidelines on the presentation of scoping reviews the methods explain the eligibility criteria, information sources, search strategy and selection of sources of evidence (inclusion/exclusion criteria), key search terms, data charting process, data items, critical appraisal, synthesis of results, reliability, and rigor. A review protocol was not developed or published for this scoping review, which follows JBI methodology (Peters et al., 2020).

### 2.1. Eligibility criteria (inclusions/exclusions)

The review is inclusive of all literature published in English language (articles written in other languages were included if published translations were available). Owing to the newness of the topic, we limited years considered to publication in the last 10 years (2011–2022).

Inclusion/exclusions were:

• Articles published in English between 2011 and the date of the searches (August 2022) were included.
• Included articles were research articles (using any type of study designs or research methods), evaluations or design studies, discussion/commentary, case studies, conference/symposia. Comments on articles were excluded.
• Publication status included articlesx published early online or online only. No unpublished articles were included.
• Included articles described or closely relate to the design, implementation, use, views, or perception of AI technologies (as defined above). Articles relating to “non-AI” technologies (e.g., electronic health records, information communication technologies, social media, online simulation training) were excluded.
• Included articles related to compassion (according to the key search terms above). Other related concepts and terms (dignity, sympathy, kindness, altruism, solidarity) were not included.
• Included articles related to healthcare contexts (any healthcare settings, health professional groups, patient or client groups, students in training), any type of healthcare interventions or practices including self-compassion. Articles outside of healthcare contexts were excluded (i.e., animal health, farming, engineering, architecture, meteorology).

### 2.2. Search process

Preliminary searches were undertaken (using Google search) in September-December 2021 to inform the review topic and questions. The final searches were conducted in August 2022. Information sources were (1) Web of Science (Science Citation Index, Social Sciences Citation Index, Arts and Humanities Citation Index, Conference Proceedings Citation Index, Book Citation Index, Emerging Sources Citation Index, covering over 12,000 high impact journals) (2) PubMed (covering biomedical literature from MEDLINE, life science journals, and books). These sources were chosen because they index extensive health and healthcare research journals as well as computing, data science, information technology, and design sciences. No other sources were used as the low specificity of the searches would have rendered an unfeasible number of returns for screening (Peters et al., 2020). The searches were performed by two experienced researchers (EM and TZ).

Table 2 presents summary information about the electronic searches and results for Web of Science and PubMed databases (Table 2). The table sets out how the constructs were searched using OR and combined using AND functions. The much larger number of articles returned by Web of Science for the AI technologies construct reflects the scope of this database beyond medicine and healthcare. The compassion construct and the healthcare construct retrieved similar numbers of articles for both databases.

**TABLE 2:** Search results.

AI technologies construct	Compassion construct	Healthcare construct	Number of articles (search returns)
AND	AND	AND	
Affective computing OR Artificial intelligence OR Automation OR Bioinformatics OR Chatbot OR Computer-assisted OR Data mining OR Decision support systems OR Deep learning OR Digital health OR eHealth/e-health OR electronic health OR Health app OR Human machine systems OR Information systems OR Machine learning Medical informatics OR mHealth OR Neural networks, Computer OR Natural language processing OR Robotics OR Smartphone OR Sentiment analysis OR Virtual reality OR Wearable	Compassion OR Empathy OR Self-compassion OR Compassion fatigue	Health care OR Health-care OR Healthcare	3,124
**Web of science database (2022-08-24)**			
6,912,998	50,252	2,108,971	1,312
**PubMed database (2022-08-25)**			
2,271,897	45,967	2,619,101	1,812

### 2.3. Screening

**FIGURE 1:**
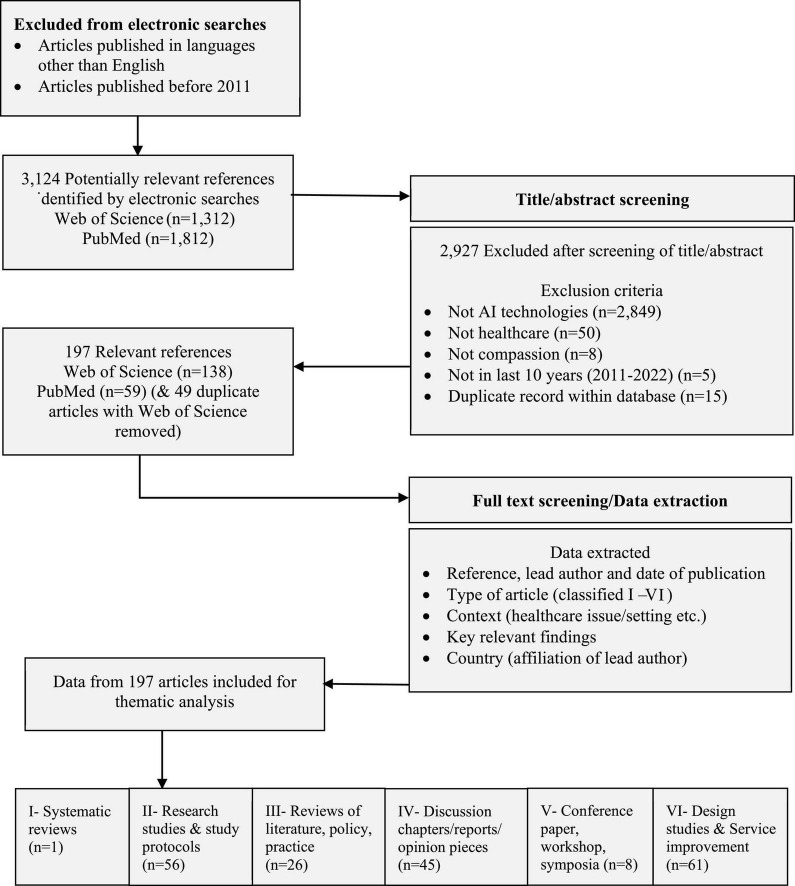
Inclusion flow diagram.

A total of 3,124 articles were identified (Web of Science 1,312 articles, PubMed 1,812 articles). The screening process was to systematically assess eligibility of each article by reading the title and abstract of all returned articles and applying inclusion/exclusion criteria. If articles were considered eligible for inclusion the full article was accessed online. A record of the reasons for exclusion of articles was maintained to support rigor and reliability. Figure 1 illustrates the screening process and information about the article type of the 197 included articles.

### 2.4. Data charting

Data from included articles were extracted to bespoke data tables (using Microsoft Word) designed to hold data about the article and content items relating to the four review questions. Tables were piloted with 10 articles; small adjustments to headings and formatting were made. Categories of data that were extracted were (1) Reference: Author/Date of publication (and citation) (2) Type of article (Categories I–VI, see below) (3) Aim/Context (e.g., healthcare issue/setting etc.) (4) Key relevant findings (5) Country (based on first author). Data charting was done by one experienced qualitative researcher (EM). No other processes were used to obtain or confirm data from investigators/authors. Key findings/points from articles were identified from abstracts and/or full texts where relevant to the review questions. As this was a scoping review not a systematic review of research evidence, selection of key information did not give weighting to research articles or aim to combine value outcomes from research studies. Information about research methods and participants was captured when relevant to contextualize key findings. For design studies information about specific AI technologies was captured where available.

### 2.5. Analysis

The analysis of included articles used a basic assessment of quality using article type. During charting of the data article type was coded (Article Type: I-Systematic review, II-Research studies and study protocol, III-Review of literature/policy/practice, IV-Discussion chapters/report/opinion piece, V-Conference paper/workshop/symposia, and VI-Design study and service improvement) to gain an overview of the data not to make judgments about research quality or to combine evidence of outcomes. Thematic analysis used an inductive-deductive approach (Mays et al., 2001) to generate categories from the review questions and the data itself. The thematic analysis began with “familiarization” to build up an understanding of the nature and content of included articles, “identification” of emerging themes during the process of data extraction, and “synthesizing” key findings or issues. A multidisciplinary team assessed 52 emerging themes for resonance and relevance to research and practice, which were refined into 24 themes.

### 2.6. Reliability and rigor

A multidisciplinary team ensured that the topic and focus of the review had resonance with the challenges and problems in their areas of practice. The review used an established review process (Arksey and O’Malley, 2005). Reliability of the search process was supported by using defined search terms and using two robust sources of data for a comprehensive search of published literature (Peters et al., 2020). Rigor of screening was supported by using defined inclusion/exclusion criteria, consistency of screening decisions, and maintaining a record of the reasons for exclusion (detail in Figure 1). A record of duplicates within sources and between data sources was maintained. Rigor in the identification of themes (Mays et al., 2001) was supported by team discussions and reflections on resonance and meaning of emerging themes and relevance to the review questions. For transparency information about all included articles is provided (Supplementary Appendix 1).

## 3. Results

### 3.1. Overview of the included literature

#### 3.1.1. Included articles

The searches identified 3,124 potentially relevant articles. All were assessed for eligibility based on titles/abstracts. A total of 197 relevant articles were included (literature tables in Supplementary Appendix 1). Total included articles = 197 (6.3% of 3,124 screened).

#### 3.1.2. Year of publication

The number of articles increased steadily over 10 years: 2011 (*n* = 1), 2012 (*n* = 1), 2013 (*n* = 3), 2014 (*n* = 2), 2015 (*n* = 4), 2016 (*n* = 6), 2017 (*n* = 15), 2018 (*n* = 15), 2019 (*n* = 34), 2020 (*n* = 32), and 2021 (*n* = 47). From Jan-Aug 2022 (*n* = 35 articles).

#### 3.1.3. Article types

Approximately a third of the articles were categorized as VI-Design studies (30.9%, *n* = 61) (concept development, proof of concept, design evaluation, and service improvement). Nearly a third of the articles were II-Research study (*n* = 56, 28.4%) (including experimental/intervention studies, qualitative research, survey research, mixed methods, exploratory, pilot, and feasibility studies). Other categories were IV-Discussion (chapter, commentary, perspective, and opinion piece) (*n* = 45, 22.3%), III-Reviews (integrative review, narrative review, literature review, and scoping reviews) (*n* = 26), V-Conference (paper, symposia, workshop) (*n* = 8), I-Systematic review (*n* = 1).

#### 3.1.4. Article country (first author)

A third of the articles were from United States = 65 articles (32.9%), with United Kingdom = 24 articles (12.1%), Canada = 17 articles (8.4%), Netherlands = 10 articles (4.9%), Australia = 10 articles (4.9%), New Zealand = 10 articles (4.9%), Germany = 6 articles, Japan = 6 articles, Italy = 5 articles, and Taiwan = 4 articles. Articles from other countries (40, 19.9%) were: 3 articles each from France, India, Republic of Ireland, Spain, Sweden, Switzerland, 2 articles each from Bangladesh, Korea, Norway, Pakistan, Philippines, Singapore, 1 article each from Belgium, China, Denmark, Finland, Greece, Lebanon, Malaysia, Qatar, Republic of Korea, Romania.

#### 3.1.5. Research articles

The review identified one systematic review (Bevilacqua et al., 2020) on personal health coaching. Of the 56 articles that were classified as II-Research study, more than two-thirds were studies using an intervention or experimental methods (*n* = 42, 73.6%) including one randomized controlled trial (RCT) in an educational setting (Johanson et al., 2019). 11 were qualitative studies (interviews, survey research, focus groups, consensus building approaches, ethnography). Other methods were mixed methods (1), feasibility study (1), pilot study (1).

#### 3.1.6. Frequency of key words

Articles most frequently mentioned the term “empathy” (113 articles). Nearly a quarter of the articles used the term “compassion” in their title or abstract (41 articles). Few articles used the term “self-compassion” (10 articles) or “compassion fatigue” (2 articles).

#### 3.1.7. Themes in the data

There are four overarching themes relating to the review questions and 24 themes, as illustrated by Table 3.

**TABLE 3:** Overview of themes in the literature.

**1. Developments and debates (7 themes)**
• Concerns about AI ethics, healthcare jobs, and loss of empathy (25 articles)
• Human-centered design of AI technologies for healthcare (16 articles)
• Optimistic speculation AI technologies will address care gaps (12 articles)
• Interrogation of what it means to be human and to care (11 articles)
• Recognition of future potential for patient monitoring, virtual proximity, and access to healthcare (10 articles)
• Calls for curricula development and healthcare professional education (5 articles)
• Implementation of AI applications to enhance health and wellbeing of the healthcare workforce (2 articles)
**2. How AI technologies enhance compassion (10 themes)**
• Empathetic awareness (15 articles)
• Empathetic response and relational behaviour (12 articles)
• Communication skills (12 articles)
• Health coaching (11 articles)
• Therapeutic interventions (8 articles)
• Moral development learning (8 articles)
• Clinical knowledge and clinical assessment (7 articles)
• Healthcare quality assessment (6 articles)
• Therapeutic bond and therapeutic alliance (5 articles)
• Providing health information and advice (3 article)
**3. Gaps in knowledge (4 themes)**
• Educational effectiveness of AI-assisted learning (11 articles)
• Patient diversity and AI technologies (10 articles)
• Implementation of AI technologies in education and practice settings (8 articles)
• Safety and clinical effectiveness of AI technologies (4 articles)
**4. Key areas for development (3 themes)**
• Enriching education, learning, and clinical practice (10 articles)
• Extending healing spaces (9 articles)
• Enhancing healing relationships (7 articles)

### 3.2. Developments and debates

#### 3.2.1. Concerns about AI ethics, healthcare jobs, and loss of empathy (25 articles)

This was the strongest theme of the literature and conveyed manifold concerns about AI ethics and regulation (Zelmer et al., 2018; Abdullah et al., 2021); ethical design and use of AI technologies in healthcare contexts (Sikstrom et al., 2022); concerns about data privacy, data biases and data collection (Harris, 2021; Ostherr, 2022); as well as concerns about trust, care quality, and liability (Davenport and Kalakota, 2019; Sanal et al., 2019). There is a strong anticipation perspective relating to concerns about role replacement (Johnston, 2018; Blease et al., 2019; Bridge and Bridge, 2019; Powell, 2019; Blease et al., 2020; Doraiswamy et al., 2020; Alrassi et al., 2021) and which parts of healthcare practice, can and should be entrusted to AI technologies (Loftus et al., 2020; Nadin, 2020). Concerns about role replacement discuss the enduring role of critical human attributes for safe and effective healthcare (Joda et al., 2020; Irfan, 2021). Speculation about the replacement of nurses with robot nurses has led to theoretical development on the interrelationship of technological competency as caring and acknowledgment that AI technologies are already fundamental to the delivery of high-quality healthcare (Locsin, 2017; Buchanan et al., 2020). Research on patient’s views about future uses of AI technologies echoes professional’s concerns regarding trust, communication, regulation, liability risks, cyber-security, accuracy, and loss of human empathy toward patients (Slomian et al., 2017; Esmaeilzadeh et al., 2021; Raja et al., 2021; Zhang et al., 2021; Visram et al., 2022).

#### 3.2.2. Human centered design of AI technologies for healthcare (16 articles)

The second strongest theme of this literature reflects broader debates about design ethics and using human-centered design approaches (HCD) to generate empathetic technological responses to health needs (Portz et al., 2020). In HCD processes designers are felt to gain empathetic understanding by working closely with end users, such as stroke patients to co-design AI technologies to support health and recovery (Willems et al., 2021). User-centered participatory design methods (e.g., interviews, workshops, trials of prototypes) narrow the gap between designers and users by supporting inclusion and engagement in the design process (Hou et al., 2020; Tiersen et al., 2021). For example, user-centered research with 15 people after stroke, led to the idea and creation of a character Stappy for a meaningful interface to support empathy in the use of a sensor-feedback system that enables stroke patients to walk (Jie et al., 2020). Research using co-design methods with young people with type 1 diabetes exposed a radically different view of technology than either their parents or practitioners, illustrating the need to involve target end-users in design (Pulman et al., 2013). This literature suggests HCD supports compassion in healthcare by creating methods and opportunities for inclusion in the design of technologies that address real and significant needs in people’s lives (McCarthy et al., 2020; Majid et al., 2021) as well as promoting trust that empathy will be preserved and acceptance of new AI technologies in a healthcare space (Zhang et al., 2021). HCD to develop an electronic crutch for paralyzed people has been described as a humanitarian project designed with empathy for patients in mind (Sarkar et al., 2020). HCD informs humanitarian applications of AI technologies (Fernandez-Luque and Imran, 2018); the design of “positive technology” to generate motivation and engagement (Riva et al., 2016); and “transformative technologies” to facilitate positive, enduring transformation of the self-world for the benefit of health and wellbeing (Riva et al., 2016). HCD embeds compassion within AI technology design by recognizing and engaging with human suffering, now or in the future (i.e., maintaining health) (Fritzsche et al., 2021), activities to co-design technological solutions that have utility and value for users (Mirkovic et al., 2018; Raman and McClelland, 2019); and ethical attention to when technology might not be a suitable solution (Pulman et al., 2013).

#### 3.2.3. Optimistic speculation AI technologies will address care gaps (12 articles)

There is hope in this literature, that AI technologies can preserve the “spirit” of welfare state and the principles of risks-sharing and equal access to care for all (Weil-Dubuc, 2019). Literature on social robots argues for the potential social utility of robots as treatment providers, custodial caregivers, social assistants, and home companions (Pedersen et al., 2018). Health professionals are hopeful that e-mental health technologies may offer a solution to the growing problem of unmet mental health needs, provided that human centered principles are maintained (Strudwick et al., 2020). VR technology and research on implicit bias are perceived to be tools to address bias, prejudice, cultural insensitivity, eroding levels of empathy, and social disparities of health (Jones-Schenk, 2016). In these discussions there is a collective aspiration for AI to reflect human wisdom in the provision of more compassionate (Lee et al., 2021; Ali et al., 2022) and “compassionomic” solutions to healthcare (i.e., safe and cost effective) (Trzeciak et al., 2017). Other expressions of optimism relate to the hope of improvements in service efficiency and quality (Blease et al., 2019; Kemp et al., 2020); entrepreneurial opportunities (Shepherd and Majchrzak, 2022); and the design of AI technologies that can encourage collective good and increase prosocial behavior (Day et al., 2021).

#### 3.2.4. Interrogation of what it means to be human and to care (11 articles)

Discussion of the complexity of interwoven “gossamer threads” of disparate, conflicting information about technologies in society raises questions about human development and empathetic response (Bjorklund, 2016). Research on transhumanism and posthumanism has explored the idea of self, soul, and human consciousness and what makes humans human (Fleury-Perkins and Paris, 2019; Ajeesh and Rukmini, 2022). Suggestions that AI and humans can create harmonious bios built on bioethical human properties, attitudes, and virtues (Sass, 2014), have been expressed creatively in medical arts with particular emphasis on preserving, or indeed enhancing, “3Cs” of communication, compassion, and competence (Yaghy et al., 2019). Research into VR simulation-based training suggests such technologies are valuable for cultivating humanization competencies (Jiménez-Rodríguez et al., 2021) and assessing professional moral actions (Francis et al., 2018). Authors have argued that techno-commercial motives are discordant with professional-relational foundation for care (Andersson et al., 2017); that AI technologies could fundamentally alter the way in which empathy, compassion and trust are currently regarded and practiced in healthcare (Kerasidou, 2020); and that failing to understand difficult to quantify human inputs into AI-based therapeutic decision-making processes could lead to important errors in clinical practice (Brandt et al., 2018; Kerr and Klonoff, 2019).

#### 3.2.5. Recognition of future potential for patient monitoring, virtual proximity, and access to healthcare (10 articles)

Studies of healthcare professionals show they value the capabilities of AI technologies for remote monitoring of patient’s physical and mental health status, and the advantages of virtual proximity for maintaining compassionate connection (Montayre, 2018; Walker et al., 2020). Although technologies are being developed for remote monitoring of older people in their own homes (Yokoo et al., 2020), little is known about clinical effectiveness or patient outcomes (Bouabida et al., 2021). Virtual proximity is also recognized as a benefit of interventions for mental health that use ecological momentary interventions (EMIS), a specific type of mobile health that enables patients to access interventions in each moment and context of daily life called a “therapist in your pocket approach” (Schick et al., 2021) such as encourage physical activity in blue-green spaces (HEART by BioAsssist) (Gallos et al., 2022). Automated topic modeling is recognized as being useful for personalized digital self-compassion interventions and overcoming barriers to traditional care (van der Lubbe et al., 2022). eHealth coaching for older people’s self-management may have benefits for addressing unmet need in mental health services (Bevilacqua et al., 2020). There is optimistic debate concerning the potential to increase access to health information and advice using widely available conversational agents (such as Apple Siri, Google Assistant, Amazon Alexa, and Microsoft Cortana) if the safety and effectiveness of these systems can be improved (Kocaballi et al., 2020a). Embodied conversational agents (ECA) (i.e., a lifelike virtual human) could have potential for engaging and motivating users for health-related learning and behavioral change (Scholten et al., 2017).

#### 3.2.6. Calls for curricula development and healthcare professional education (5 articles)

There is a sense of urgency in this literature to teach health professionals essential digital skills and overhaul curricula (Konstantinidis et al., 2022) as well as to introduce AI technologies in educational environments in safe and effective ways that address risks and responsibilities (Combs and Combs, 2019). Such as the opportunities and implications of using standardized virtual patients (VPs) (Gavarkovs, 2019), patient clinical scenarios (Yang et al., 2022), and digital simulations (Patel et al., 2020).

#### 3.2.7. Implementation of AI applications to enhance health and wellbeing of the healthcare workforce (2 articles)

Few studies have investigated the use of AI technologies for healthcare provider wellbeing but there does seem to be a link with compassion for staff and patients. Examples are music virtual reality for healthcare workers (Hayakawa et al., 2022) and The Provider Resilience app for mental health care providers (Wood et al., 2017).

### 3.3. How AI technologies enhance compassion

This section of the results presents themes relating to applications and uses of AI technologies to enhance compassion in healthcare, as reported in the literature. Ten themes are presented in order of their strength in the literature (number of articles mentioning the issues not number of technologies).

Table 4 maps the themes in this section to the working definition of compassion (the 5 elements defined in Section “1.4 Definitions and scope”). Organizing the themes in this way, Table 4 highlights the different ways that AI technologies are associated with compassion in healthcare. No articles or studies were found in this literature which map onto the compassion element, “(4) Making a judgment about the suffering (the need to act).” It is unclear why, but this lack of discussion could reflect assumptions about clinical judgment (i.e., perceived to be an objective assessment) and compassion (i.e., perceived to be a felt emotion) in healthcare. As a result of the findings in this section, and the mapping work, the wording of element five has been altered from the working definition (Engaging in a behavior with the intention to alleviate the suffering) to “Responding with an intention to alleviate the suffering.” This change marks a move away from only perceiving compassion in terms of a human behavioral response to suffering toward a broader understanding of compassion as a system as well as the possibility of an AI or human response that is not behavioral i.e., not only visible acts of caring but also digital empathetic responses, provision of health information, advice or coaching by AI technologies. An additional sixth element of the compassion construct emerged from this analysis of the literature (“Attention to the effect and outcomes of the response,” illustrated by the final row in Table 4). The implications of this additional element for the reconceptualization of compassion are discussed later (Section “3.3 Reconceptualizing compassion as a human-AI system of intelligent caring”).

**TABLE 4:** Artificial intelligence (AI) technologies mapped to compassion in healthcare.

Compassion	Healthcare	AI technologies (applications and studies reported in the literature)
(1) Awareness of suffering (e.g., pain, distress, risk, disadvantage)	Empathetic awareness (15 articles)	← Immersive VR experiential learning for healthcare professionals e.g., experiencing old age, disabilities (Brydon et al., 2021; Demarinis, 2022; Kim and Chun, 2022) ← Empathy training VR technologies for specific conditions (e.g., dementia, Parkinson’s disease) (Ball et al., 2015; Slater et al., 2019; Hirt and Beer, 2020; Sung et al., 2022; Torrence et al., 2022) ← Old age simulation suits for education, research, or technology design (Groza et al., 2017; Schmidt et al., 2022) ← Tele-empathy (Palanica et al., 2019) ← Serious games (Sterkenburg and Vacaru, 2018), perspective switch (Buijs-Spanjers et al., 2019; Ma et al., 2021) ← Robot attentional behaviours (Tanioka, 2019; Tanioka et al., 2019; Tanioka et al., 2021)
	Moral development learning (8 articles)	← VR simulations for moral development learning (Wartman, 2019; Wartman and Combs, 2019) e.g., cultural competencies and anti-discriminatory communication practices (Roswell et al., 2020); promoting understanding of social determinants of health (Gillespie et al., 2021; Brammer et al., 2022) ← Safe investigation of medical decisions/care ethics using VR scenarios (Francis et al., 2018) ← Game-based VR immersions or VR simulations with virtual patients to teach social determinates of health (Amini et al., 2021; Hershberger et al., 2022)
(2) Understanding the suffering (significance, context, rights, responsibilities etc.)	Clinical knowledge and clinical assessment (7 articles)	← Immersive VR training on symptoms of disease (Jones et al., 2021) e.g., vignettes for Parkinson’s disease (Hess et al., 2022), VR training for testicular disease (Jacobs and Maidwell-Smith, 2022; Saab et al., 2022) ← Learning about anatomy and physiology of disease awareness using digital anatomy (Osis, 2021) ← Automated student skills assessment in pain assessment skills development (Moosaei et al., 2017) ← Automated patient health status and mood assessment (Yokoo et al., 2020) ← Automated assessment of Parkinson’s disease (Sabo et al., 2022)
(3) Connecting with the suffering (e.g., verbal, physical, signs, and symbols)	← Communication skills (12 articles)	← Communication skills training using virtual humans (Wu et al., 2017; Guetterman et al., 2019), VR patients (Guetterman et al., 2017; Yao et al., 2020) ← Simulated language translator/translation apps (Herrmann-Werner et al., 2021) ← Virtual worlds (VW) for communication and teamworking skills development (Mitchell et al., 2011; Wu et al., 2019) ← VR environments for communication skills and research (Sanders et al., 2021) ← Robot facial expression research (Broadbent et al., 2018; Milcent et al., 2021; Kovalchuk et al., 2022), human engagement and attention in research contexts (Johanson et al., 2019)
	Therapeutic bond and therapeutic alliance (5 articles)	← Digital therapeutic bond research in conversational agents (Darcy et al., 2021) ← Automated VR exposure therapies (VRETs) for patient adherence and efficacy of self-guided treatments (Brandt et al., 2018; Miloff et al., 2020) ← Digital therapeutic alliance research (Tong et al., 2022) ← Using apps to promote access and adherence to treatment for people who experience stigma (beneficent dehumanization of care) (Palmer and Schwan, 2022)
(4) Making a judgement about the suffering (the need to act)	-	-
(5) Responding with an intention to alleviate the suffering	Empathetic response and relational behaviour (12 articles)	← Robot/artificial emotional response behaviours (artificial empathy) (Kennedy et al., 2012; Pepito et al., 2020; Kerruish, 2021; Montemayor et al., 2021) ← Empathetic chatbots (Amini et al., 2013; Liu and Sundar, 2018; Daher et al., 2020) ← Empathetic medical conversations (Yun et al., 2021) digital voice (James et al., 2021) ← Empathetic service robots (Kipnis et al., 2022) ← Therapeutic zoomorphic robots (Kerruish, 2021)
	Providing health information and advice (3 articles)	← Conversational agents for health needs, safety or lifestyle information and advice (Kocaballi et al., 2020a) ← Web app that provides cancer disease related information to patients (Papadakos et al., 2017) ← AI-generated diagnosis information for radiology patients (Zhang et al., 2021)
	Health coaching (11 articles)	← Virtual health coaches (Kennedy et al., 2012; Bevilacqua et al., 2020), smoking cessation (He et al., 2022), weight-loss (Stein and Brooks, 2017), self-management of depression (Inkster et al., 2018), and chronic disease self-management (Hernandez, 2019) ← Therapeutic chatbots for mental health (Lee et al., 2019; Valtolina and Hu, 2021) ← Digital self-compassion interventions using established therapeutic methods (Stenberg et al., 2015; Rodgers et al., 2018; Boggiss et al., 2022)
	Therapeutic interventions (8 articles)	← Dolls and robot therapies (Márquez Sánchez et al., 2020) ← Assistive robots for daily-care activities (Law et al., 2019) ← VR technologies for mental health support or development of patient’s empathetic awareness (Baghaei et al., 2019) ← Avatar-based VR therapy for empathetic understanding (van Rijn et al., 2017) ← Intelligent assistant for psychiatric counseling (Oh et al., 2017) ← Social cognition training for autism spectrum disorder (van Pelt et al., 2022) ← Immersive VR self-compassion training for self-criticism (Falconer et al., 2014) ← VR intervention for cancer patients incorporating relaxation and compassionate mind training (O’Gara et al., 2022)
(6) Attention to the effect and outcomes of the response	Healthcare quality assessment (6 articles)	← Automated healthcare quality assessment e.g., sentiment analysis of patient feedback from diverse groups of service users (Doing-Harris et al., 2017; Rahim et al., 2021) ← Automated analysis of patient and family feedback captured by interactive patient care technology in hospitals (Clavelle et al., 2019) ← Automated analysis of online health communities to inform policy for patient self-care (Panzarasa et al., 2020) ← Automated evaluation of psychotherapy services linked to training, supervision, and quality assurance (Flemotomos et al., 2022; Xiao et al., 2015).

#### 3.3.1. Empathetic awareness (15 articles)

In this literature compassion and AI technologies are most strongly associated with generating empathetic awareness in humans and robots. There is good evidence that immersive VR experiences that simulate patient experiences of illness can help healthcare professionals to understand what it is like to have a specific disease or health need (Brydon et al., 2021; Demarinis, 2022), which may translate into empathetic response or relational behaviors. For example, nursing students who virtually experienced the conditions of perioperative patients through VR blended learning showed increased levels of empathy, positive attitudes toward patient safety treatment, confidence in nursing care, and improved clinical skill performance (Kim and Chun, 2022). Multiple evaluation studies into the effects of immersive simulation for dementia suggest that an VR experience can simulate a range of aspects of dementia so that students can develop empathetic understanding (Ball et al., 2015; Slater et al., 2019; Hirt and Beer, 2020; Sung et al., 2022). However, research in the US indicates that both VR and physical delivery formats of a dementia tour can be effective, but university students on healthcare courses (*n* = 41) reported poorer attitudes about living with advanced dementia and feeling less prepared for caregiving in both approaches (Torrence et al., 2022). Interesting research in Romania (Groza et al., 2017) and Germany (Schmidt et al., 2022) into the use of age simulation suits shows that “instant ageing” can generate more negative expectations regarding older age and reenforced stereotypes. Tele-empathy is a promising emerging field where clinicians and carers can get a sense of what the patient is experiencing physically, such as tremors in Parkinson’s disease (Palanica et al., 2019). Research on serious games for medical education (The Delirium Experience) shows certain game features, being able to “switch perspective,” can enhance medical student empathy if they play the game from the patient or nurse perspective (Buijs-Spanjers et al., 2019; Ma et al., 2021). Experiments in the Netherlands on a serious game for care workers for people with disabilities (The world of EMPA) showed participation did not enhance empathy for disabled people but it did decrease personal distress in care workers (Sterkenburg and Vacaru, 2018). In robotics, experiments in Japan (Pepper robot) (Tanioka, 2019; Tanioka et al., 2019; Tanioka et al., 2021) identify the need to develop “listening” and “gaze” together with the fidelity of responses, to mimic empathetic awareness.

#### 3.3.2. Empathetic response and relational behavior (12 articles)

In theory, AI technologies cannot feel or express genuine empathy, hence the term empathy* has been suggested as a term to differentiate real empathy from artificial empathy (Montemayor et al., 2021). Nonetheless, empathy display and relational behavior are significant research themes in dialog systems development and robotics (Kennedy et al., 2012; Liu and Sundar, 2018; Pepito et al., 2020; Kerruish, 2021). Studies with patients have shown that most people prefer medical assistant chatbots that mimic empathy (Amini et al., 2013; Liu and Sundar, 2018; Daher et al., 2020), this is particularly true for users who are initially skeptical about machines possessing social cognitive capabilities. However, research in Korea (Yun et al., 2021) shows there is a discrepancy between expressed behavioral intentions toward medical AI and implicit attitudes (detected in brain scans) which shows people respond differently to the same conversation if it is delivered by a human doctor or medical AI. Other research has modeled an empathetic voice for healthcare robots, to show that people prefer robots that have an empathetic voice (James et al., 2021). A study of service robots for people with disabilities showed that they perceive robots as being able to stimulate and regulate emotions by mimicking cognitive and behavioral empathy, but unable to express affective and moral empathy, which they felt was essential for the feeling of “being cared for” (Kipnis et al., 2022). Analysis of human empathy toward a therapeutic zoomorphic robot (Paro) and a health care support robot (Care-O-Bot) draws attention to how the cultivation of user empathy toward robots influences patient sociality and relational interactions between human care providers (Kerruish, 2021).

#### 3.3.3. Communication skills (12 articles)

Artificial intelligence (AI) technologies are associated with compassion through helping to improve health professional’s verbal and non-verbal communication skills (Wu et al., 2017; Guetterman et al., 2019), for example breaking bad news to a virtual human program (Guetterman et al., 2017), and communicating with suicidal virtual patients (Yao et al., 2020). Students that engaged in a 90-min simulation with a standardized patient (SP) and a language translation app (LTA iTranslate Converse) rated the teaching unit as being excellent but wanted practical training with an SP plus a simulated human translator first on how to maintain empathy in patient-physician communication mediated by LTA (Herrmann-Werner et al., 2021). Online virtual worlds (VW) (such as Second Life, Altspace, Rec Room, Google Earth VR) are rapidly becoming part of everyday life for children and adults (in 2020 Roblox had 150 million active users), and VWs have been used to improve patient-centered communication skills and student teamworking (Mitchell et al., 2011; Wu et al., 2019). A scoping review of virtual environments (VE) for clinical communication skills (Sanders et al., 2021) suggests multiple uses for enhancing clinician’s communication and empathy skills, as well as utility for communication research purposes. Evidence on effective doctor-patient communication has been applied as principles to robot-patient communication (Broadbent et al., 2018) and empathy display/facial expression (Milcent et al., 2021; Kovalchuk et al., 2022), to increase human engagement and attention in research contexts (Johanson et al., 2019).

#### 3.3.4. Health coaching (11 articles)

There is a strong association between AI technologies (i.e., virtual coaches and health promoting chatbots) and compassion in health coaching to encourage and motivate positive health-related behavior change such as physical exercise (Kennedy et al., 2012; Bevilacqua et al., 2020), smoking cessation (He et al., 2022), weight-loss (Stein and Brooks, 2017), self-management of depression (Inkster et al., 2018), and chronic disease self-management (Hernandez, 2019). An interesting experiment with a self-compassion chatbot (Vincent) (Lee et al., 2019) revealed participation in self-compassion exercises enhanced self-compassion, particularly when participants were asked to care for the chatbot itself (versus the chatbot caring for them). In Italy a chatbot designed for older adults (Charlie) (Valtolina and Hu, 2021) can alert users to health commitments and medicines, connect remotely with doctors, family, entertain and assist elders using motivational strategies based on gamification, active notifications, and promotion of self-compassion and preventive mental healthcare. Virtual health coaches can improve self-compassion by incorporating established therapeutic methods to remodel thoughts, change behaviors and enhance relationships with self and others (Stenberg et al., 2015; Rodgers et al., 2018; Boggiss et al., 2022).

#### 3.3.5. Therapeutic interventions (8 articles)

The literature suggests an association between AI technologies and compassion occurs through therapeutic interventions. Interesting examples are dolls and robot therapies for Alzheimer’s Disease, autism spectrum disorder, stress, or depression which can evoke different verbal, motor, and emotional reactions in patients (Márquez Sánchez et al., 2020): assistive robots for daily-care activities, health-promoting behaviors, and companionship (Law et al., 2019); VR perspective-switching to treat young people with mental health problems by switching perspective (Baghaei et al., 2019); avatar-based VR therapy to develop empathetic understanding in a therapeutic community prison in the UK (van Rijn et al., 2017); and use of an intelligent assistant for psychiatric counseling (Oh et al., 2017). In one study social cognition training for adults with autism spectrum disorder (ASD) was perceived to be useful but lacking ecological validity (authenticity to real world triggers and situations) (van Pelt et al., 2022). Immersive VR therapy has exploited the known effects of identification with a virtual body to overcome self-criticism in healthy women (Falconer et al., 2014). Another study (The SafeSpace study) co-designed and tested a VR intervention for cancer patients that incorporates relaxation and compassionate mind training to enhance feelings of wellbeing (O’Gara et al., 2022).

#### 3.3.6. Moral development learning (8 articles)

Artificial intelligence (AI) technologies (VR applications) can support compassion through moral development learning in accordance with ethical standards (Wartman, 2019; Wartman and Combs, 2019). For example, by enhancing participant’s cultural competencies and anti-discriminatory communication practices (Roswell et al., 2020); promoting understanding of social determinants of health (social, physical, and economic conditions that impact upon health) (Gillespie et al., 2021; Brammer et al., 2022); and facilitating the safe investigation of simulated moral actions in aversive moral dilemmas (Francis et al., 2018). Interactive game-based VR immersions and VR simulations have been shown to heighten health professional’s awareness and cultural sensitivity to health equity issues (Amini et al., 2021; Hershberger et al., 2022).

#### 3.3.7. Clinical knowledge and clinical assessment (7 articles)

The literature suggests that AI technologies can support compassion by helping health professionals to understand and respond to human suffering. Specific examples include immersive VR training on psychological symptoms of dementia (Jones et al., 2021); VR training using vignettes for Parkinson’s disease (Hess et al., 2022), and VR training for testicular disease (Saab et al., 2022). However, benefits of student engagement and perceived learning associated with immersive learning may not translate into better exam scores or clinical skills (Jacobs and Maidwell-Smith, 2022) without sufficient preparation or teaching support (Saab et al., 2022). Another emerging field is digital anatomy, which uses digital replicas of historic specimens to foster understanding and empathy through discussion of ethics, bias, and social aspects of health and disease (Osis, 2021). Student’s understanding of pain can be assessed by using facially expressive robotic patient simulators (Moosaei et al., 2017). AI technologies are also being developed to support clinical assessment. Examples include trials in Japan to develop automated health and mood assessment systems (motion sensors and human emotion detection connected *via* the internet of things) to assess older adults in home settings (Yokoo et al., 2020); and technology development in Canada (automated video capture and spatial-temporal analysis) to accurately predict clinical scores of parkinsonism (Sabo et al., 2022).

#### 3.3.8. Healthcare quality assessment (6 articles)

In the literature AI technologies are associated with compassion through automated healthcare quality assessment. Specific examples are the use of natural language processing and patient’s online social media comments to capture service feedback information from diverse groups of service users (Doing-Harris et al., 2017; Rahim et al., 2021); automated analysis of patient and family feedback captured by interactive patient care technology in hospitals (Clavelle et al., 2019); a large-scale network study of online health communities to inform future policy interventions for patients’ self-care (Panzarasa et al., 2020). At the clinical level, automated evaluation of psychotherapy skills using speech and language technologies can augment experts’ capabilities in training, supervision, and quality assurance of services (Xiao et al., 2015; Flemotomos et al., 2022).

#### 3.3.9. Therapeutic bond and therapeutic alliance (5 articles)

Artificial intelligence (AI) technologies are associated with compassion through extending or enhancing human and digital therapeutic bond and therapeutic alignment (Lindner, 2021). For example, a study of a cognitive behavioral therapy conversational agent (Woebot) demonstrated therapeutic bond scores that are comparable to traditional therapy within 5 days of initial app use (Darcy et al., 2021). Automated VR exposure therapies can improve adherence and efficacy of self-guided treatments (Miloff et al., 2020) and address challenges of asynchronous feedback in traditional care (Brandt et al., 2018). Learning from persuasive/positive technology and human-app attachment can potentially help to foster a sense of empathy, build tasks and goals, and develop bonds and digital therapeutic alliance (Tong et al., 2022). Medical AI carebots can overcome barriers to care and adherence to treatment for people who experience stigma (the concept of beneficent dehumanization of care) (Palmer and Schwan, 2022).

#### 3.3.10. Providing health information and advice (3 articles)

Artificial intelligence (AI) technologies can support compassion by providing health information and advice but the evidence of effectiveness of specific technologies is underexplored. Commonly available conversational agents (e.g., voice commands on smartphones) are currently limited in their ability to pick up on conversational cues for health needs and effectively advise on health safety or lifestyle prompts (Kocaballi et al., 2020b). A web app can replicate cancer library functions but with limitations associated with explaining information and supportive care (Papadakos et al., 2017). Radiology patients perceived AI generated diagnosis information to be useful for confirming the doctor’s opinions and preparing for the consultation, but patients saw AI technology as having drawbacks of cyber-security, accuracy, and lack of empathy toward patients (Zhang et al., 2021).

#### 3.3.11. Gaps in knowledge

This section of the results presents themes relating to gaps in knowledge and underexplored potential of AI technologies as described in the literature.

#### 3.3.12. Educational effectiveness of AI-assisted learning (11 articles)

This theme in the literature reflects an undercurrent of uncertainty about the effectiveness of specific types of AI technologies in health professional education contexts (Jones et al., 2021; Sukhera and Poleksic, 2021) as well as the possible negligible benefit (Navarrete et al., 2021) or loss of benefits associated with replacing existing educational methods with technologies [such as the benefits of involving real patients in teaching as described by Abeyaratne et al. (2020)]. The issue is not that technologies cannot generate empathy in some groups of learners, but that empathy might not translate into longer-term prosocial caring behaviors in healthcare systems (Gillespie et al., 2021; Beverly et al., 2022). For example, VR dementia training may not benefit all learners and VR may differentially assist leaners of different ages and English-speaking backgrounds, suggesting that more research is needed to understand for which variables and for whom VR is a useful teaching tool (Jütten et al., 2018; Stargatt et al., 2021). VR provides a small snapshot of the vicissitudes of living with an illness or disability that might leave a false impression of what patients “like that” feel (Dean et al., 2020). It could be that other types of technologies, less standardized (more complex and diverse) virtual patients (Shorey et al., 2019), or digital anatomy could inform professional training and enhance student learning or empathy more effectively (Osis, 2021), but this is unknown. Learning technologies that have “point-of-view” functions may enable students to see issues from different perspectives (Levett-Jones et al., 2017) and diverse service users’ experiences (Riches et al., 2022) which could benefit caring relationships.

#### 3.3.13. Patient diversity and AI technologies (10 articles)

There are significant gaps in understanding about how patient diversity relates to AI technologies and compassion. These gaps relate to “high” and “low” users of technologies (Inkster et al., 2018); differences in acceptability of technologies e.g., service robots for healthcare (Giambattista et al., 2016), psychological evaluation (Rossi et al., 2020) or self-management technologies (Mirkovic et al., 2018); language and communication style preferences (Herrmann-Werner et al., 2021; Boggiss et al., 2022; Eagle et al., 2022). Race-concordance has emerged as an important factor in the design and use of virtual patients and virtual clinicians, but the implications for teaching and practice are unclear and underexplored (Halan et al., 2015; Krieger et al., 2021). For example, in one design study black men (*n* = 25) designed a Black male virtual clinician (VC) that was named Agent Leveraging Empathy for eXams (ALEX) and referred to as “brother-doctor”; participants wanted to interact with ALEX over their regular doctor (Wilson-Howard et al., 2021). While automated services could extend access to psychological support, research into digital therapeutic alliance is needed to ensure AI technologies work for diverse patient groups (Scholten et al., 2017; Grekin et al., 2019; Tong et al., 2022). The first therapeutic alliance instrument developed for use with embodied virtual therapists is the Virtual Therapist Alliance Scale (VTAS): preliminary assessments suggest that alliance toward a virtual therapist is a significant predictor of treatment outcome (Miloff et al., 2020). Patient diversity also needs to be considered in relation to equipping virtual agents with more human-centric prosocial rule breaking, which is a common beneficial feature of human ethical decision-making behavior that is difficult to mimic in AI technologies (Ramanayake et al., 2022); as well as to support patient’s “social convoy” (Portz et al., 2020) (i.e., family members, friends, neighbors, formal caregiving supports) to facilitate appropriate involvement and information sharing.

#### 3.3.14. Implementation of AI technologies in education and practice settings (8 articles)

It is currently unclear how the implementation of AI technologies might affect compassion in different contexts of healthcare (Verma et al., 2021), such as medical imaging (Bleiker et al., 2020) or intensive care (Price, 2013). Little is known about how AI technologies and compassion might relate to service efficiency or patient care (Kocaballi et al., 2020a); or public perceptions of AI capabilities (Chew and Achananuparp, 2022). Future research is needed to explore the role and implementation of VR for enhancing empathy in various real-world contexts, and the mediating role of individual differences in use of AI-driven interventions (Louie et al., 2018; Nisha et al., 2019). Implementation of AI technologies in healthcare systems requires development and implementation of new curricula and new approaches to teach students how to interact with AI technologies, learn within interactive learning environments, and manage Al systems (Srivastava and Waghmare, 2020).

#### 3.3.15. Safety and clinical effectiveness of AI technologies (4 articles)

The effectiveness of VR based “switching perspective” technologies (encouraging a self-compassionate lens) for early intervention for mental health issues is promising but research is needed to explore safety and privacy issues in real-world contexts (Baghaei et al., 2021). Further research into general conversational agents is needed to establish guidelines for designing safe and effective response structures for different prompt types (Kocaballi et al., 2020b). The potential capabilities and risks of active assistance technologies is underexplored and there is a need to consider informatics methods and algorithms more fully for safety and ethical reasons (Kennedy et al., 2012). It is unclear how to maintain the initial benefits and permanence of behavior change produced by short-term virtual health coaching interventions (Bevilacqua et al., 2020) and this needs further research to attain lasting clinical benefits.

### 3.4. Key areas for development

#### 3.4.1. Enriching education, learning, and clinical practice (10 articles)

Findings in the literature suggests there is great potential for AI technologies to enhance underexplored elements of compassion by enriching education, learning and clinical practice (Sukhera and Poleksic, 2021; Saab et al., 2022). There appears to be an “engagement factor” (Navarrete et al., 2021) associated with immersive VR environments which could be further explored for student engagement and empathy awareness as well as other elements of compassion, such as making a judgment about the suffering (the need to act). Understanding suffering could be enhanced by using immersive technologies in combination with new types of haptic technologies (technologies that create an experience of touch by applying forces, vibrations, or motions to the user) (Ling et al., 2020) or existing tele-empathy applications (Ho et al., 2017; Palanica et al., 2018). Learning from self-compassion apps about identification with a virtual body (Falconer et al., 2014) could be integrated into immersive VR interventions to enhance clinical knowledge and clinical assessment skills in order to better understand suffering associated with the body (Plotzky et al., 2021). It could be useful to take learning from co-designed virtual health coaching apps into educational applications (Atif et al., 2022). There is potential to use VR technologies with clinical simulations and virtual patients to enhance approaches to moral development learning and ethical clinical decision making (Francis et al., 2018). Future research could explore the notion of beneficent dehumanization of care (e.g., to overcome stigma, stereotyping, negative emotions, or regret) and the implications for professional training and education (Palmer and Schwan, 2022). There is a clear need for research and education on AI technologies in relation to global humanitarian health analysis and responses (Fernandez-Luque and Imran, 2018) which could include development of virtual health tours for different groups of health professionals to teach about health issues in different countries and regions.

#### 3.4.2. Extending healing spaces (9 articles)

Virtual and immersive spaces may have additional benefits for patients, health professionals, and students with respect to health and wellbeing outcomes that are not yet known (Gavarkovs, 2019), such as stress reduction (Michael et al., 2019). There is potential to integrate AI technologies to deliver combined physical health and wellbeing interventions for more effective mind-body interventions for patients and healthcare professionals (Rosa, 2014; Michael et al., 2019; Zheng et al., 2022). Such AI-assisted healing spaces could be devised to be individual (e.g., immersive VR) or shared virtual restorative spaces (e.g., making use of virtual worlds) drawing on known effective interventions for wellbeing. Co-therapy approaches, where community peers use avatars to share health information (Atif et al., 2022) have the potential to take clinics into communities, especially in resource-poor settings. Research on the internet of things (IoT) (Tiersen et al., 2021) opens new possibilities and challenges for seeing people’s homes as clinical spaces (Kelly et al., 2020; Bouabida et al., 2021).

#### 3.4.3. Enhancing healing relationships (7 articles)

According to the literature, AI technologies could support compassion by enhancing healing relationships. For example, by exploring and developing bonds between humans and technologies could boost engagement and efficacy of digital therapeutics (Darcy et al., 2021). It could be useful to explore further how therapeutic relationships are affected by virtual characters that exhibit certain perceived qualities such as gender (García et al., 2003) or ethnicity (Marcoux et al., 2021), to inform virtual health coach systems (Bevilacqua et al., 2020). Further research into traits and behaviors such as humor, self-disclosure, facial expressions, eye gaze, body posture, and gestures (Johanson et al., 2021) could inform effective human-robot interaction and human-human interactions in healthcare (Liu and Sundar, 2018). Cross-cultural research could inform ongoing development (in New Zealand) of an autonomous empathy system of a digital human to understand the challenges and opportunities for empathetic interactions (Loveys et al., 2022).

## 4. Discussion

### 4.1. Contribution of this review

The core contribution of this review is to demonstrate the association between AI technologies and compassion in healthcare and to elaborate on the nature and complexities of this association. Specifically, the review (1) shows the ways that AI technologies are currently being debated, developed, and used to enhance compassion in healthcare systems, so that these areas might be explored in more depth in the future (2) reconceptualizes compassion as a *human-AI system of intelligent caring* comprising six elements. These new understandings are theoretically informed, derived from an established scoping review methodology (Arksey and O’Malley, 2005; Peters et al., 2020) and a systematic process of data extraction and thematic analysis (Mays et al., 2001). A multidisciplinary team interrogated the themes and interpreted the findings for research and practice. Future development work using deliberative methods could test the validity of the findings with interdisciplinary cohorts of health professionals, educators, students, technologists, patients, and researchers, for example, to explore the themes that have been identified; and to debate priorities for future research and practice. The present review has developed and provided a set of search terms, and captured baseline data, which means the exercise could be repeated in a year or two to investigate any developments in this emerging topic area.

### 4.2. Limitations

As this scoping review only includes articles published in English it is biased toward westernized perspectives of healthcare and compassion. It does not consider alternative cultural understandings or ways of perceiving compassion, for example, the African philosophy of *ubuntu* or the Buddhist *maitrî* (aka mettâ). The literature and perspective firmly focuses on compassion for people, rather than alternative understandings of compassion for sentient beings, or the environment, which are increasingly relevant to health and healthcare services. The review did not examine whether specific AI technologies, or their use in particular interventions or contexts, are effective, usable, and adoptable. It did not use statistical tests, or percent values about adoption and/or use of different kinds of technological practices or tools and satisfaction/dissatisfaction about them or any other type of outcomes. The review does not draw on learning from other fields (e.g., AI in military ethics, automated vehicle ethics, computer generated imagery or the film industry, business hybrid systems of online/offline communication, medical crowdsourcing etc.). Identified key areas for potential are biased toward present use cases in healthcare, and biases toward applications in elderly care, dementia, and finding AI-driven solutions to an aging population. Issues relating to young people, minority patient groups, people who suffer health inequalities (Scambler, 2012) or barriers to healthcare (Powell et al., 2016), are likely to be underrepresented in the results.

### 4.3. Reconceptualizing compassion as a human-AI system of intelligent caring

It is challenging to think of compassion as a system rather than a feeling or experience, yet a systems perspective is where the meaning and value of the concept lies: offering possibilities to align and integrate motives and motivation for intelligent caring behavior in humans, AI technologies, and healthcare systems (Lansing, 2003; Levin, 2003). Compassion, in this reconceptualization, is not about managing professional virtues or mimicking emotions (Pedersen and Roelsgaard Obling, 2019) it is about combining human and AI capabilities in an integrated system of intelligent caring.

Reconceptualizing compassion as a human-AI system of intelligent caring connects thinking about compassion at the individual human level (i.e., human psychology and behavior), with compassion as a function of AI technologies (e.g., artificial empathy, artificial compassion, HCD and technology design practices), and compassion as an essential aspect of healthcare system effectiveness and human flourishing. This conceptualization allows compassion, as it is perceived and manifest in everyday healthcare practice to connect with highly technical discourses about the use of AI technologies in healthcare systems, and human-machine boundaries (De Togni et al., 2021). It offers clear elements to explore how together humans and AI technologies might become more intelligent and caring.

As noted in the findings on how AI technologies are being used to enhance compassion in healthcare (Section “2.9 How AI technologies enhance compassion”) a new sixth element of compassion emerged from the analysis of the literature (as shown in the final row of Table 4). That is, “(6) Attention to the effect and outcomes of the response.” This new element that has been identified, corresponds with previous compassion research which suggests that experiencing or witnessing helpful human interactions is an important mechanism for developing understandings about compassion (Walter et al., 2015). This additional sixth element appears to be necessary to complete a feedback cycle, so that the person, the AI technology, or the healthcare system, is aware suffering has been alleviated or not, thereby creating future motivation (Ford, 1992). This sixth element corresponds with the importance of learning over time e.g., through training or performance feedback, learning from examples of excellence as well as learning from failings in healthcare.

**FIGURE 2:**
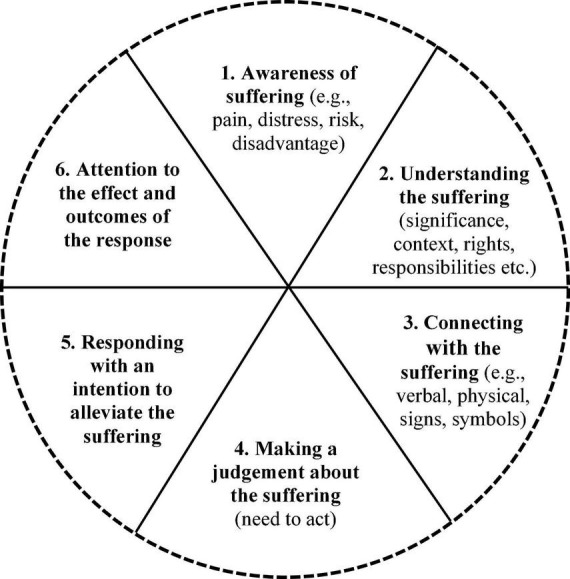
Reconceptualizing compassion.

Figure 2 draws together the elements developed from the original working definition of compassion (Section “1.4 Definitions and scope”) and informed by the review findings, to illustrate how compassion may be reconceptualized as a system of at least six interrelated component elements, which may or may not interconnect in any individual, organization, or system level to form a cyclical feedback system. Although the components are numbered 1–6, they may exist in different health systems, areas of practice, or health professional’s behaviors simultaneously. Alternatively, some or all elements may absent or underdeveloped. For example, a person may have very good empathetic awareness, but this may not translate into a decision to act or a response with the intention to alleviate suffering. At the macro level, it could be that a healthcare organization may generate responses with the intention to alleviate suffering, yet fail to connect with the suffering, meaning that patients are not consulted, are unaware, or do not feel involved in decisions about the type of response.

This reconceptualization has six component elements of compassion that are numbered 1–6 for clarity, but they are not necessarily sequential. Elements can be learnt and enhanced by individual humans and some AI technologies that have appropriate programming features (Mason, 2015, 2023). At a higher macro level, these elements of a compassion system can be developed by whole healthcare organizations, or across healthcare systems with strategic and supportive interventions. For example, healthcare professionals in training can learn to develop empathetic awareness but also to understand suffering in context, such as the provision of high-quality healthcare to address health inequalities and promote health equity. This reconceptualization of compassion, as a system, acknowledges the ethical challenges of artificial empathy, unease about virtual human’s mimicry, deception, and moral incongruence (Montemayor et al., 2021), and asserts a way forward through the authentic empathy debate. It does this by showing that it is possible for humans and AI to collectively promote collective good (Day et al., 2021). The nature of the system, through which this is made possible, is encapsulated by the six component elements of compassion. AI technologies can contribute to each, or all, of these different elements of compassion. Thus, this understanding offers a much more significant and useful contribution of AI technologies to compassionate patient care and healthcare systems compared to concerns about replacing human empathy with digital empathy.

We suggest that when all six elements of compassion are present, functioning well, and interconnected, compassion is an intelligent caring system. Compassion involves intelligence in the sense that it is a learning system that is responsive and adjusts to new information and feedback. This is because the sixth element provides feedback (to the individual, the organization, or the system) about whether compassion has occurred, and suffering has been alleviated. For example, to tell humans when to step back with AI technologies and the circumstances when a human empathetic response is what a person wants and needs to lessen their suffering (e.g., breaking bad news, end of life care, apologizing for failings in care) (Elkin, 2021).

The review shows that concerns in the literature regarding AI technologies center on the issue of whether AI technologies are fundamentally about the replication of humans (Section 3.2). The review highlights that there are a range of AI technologies in development that aim to replicate human bodies, voices, and mannerisms (e.g., affective computing, robotics, embodied virtual agents), or to imitate human relationships (digital bonds, digital therapeutic relationships etc.), or to reproduce human capabilities (e.g., job roles, skill sets, knowledge, abilities). Aiming to replicate humans and human relationships could be problematic in the longterm, not only for technical reasons or the authenticity and artificial empathy. From a sociological point of view, replication risks reformation of harmful or unfair social structures (e.g., power dynamics, status, capital, agency) (Parsons, 1982) in new forms of deceptive relationships, based on artificial emotions (Montemayor et al., 2021). However, the results on how AI technologies are being used (Section “3.3 Reconceptualizing compassion as a human-AI system of intelligent caring”) show a more positive and transformative ambitions here, in the form of innovative applications and studies of AI technologies that seek to “augment,” “enrich,” and “enhance” human lives, not to replace them.

Current applications in education and practice are providing engaging learning experiences; supporting human-human healing relationships; as well as providing some effective interventions for health and wellbeing such as therapeutic counseling. These applications are doing this in unique and original ways that are made possible through AI e.g., immersion, VR, perspective switch, avatars, simulation suits. Rather than evaluating the extent to which a given technology has successfully replicated human capacities or designing technologies according to understandings of the human body or mind, future research might instead seek to transform rather than replicate pre-existing human or societal systems with their biases, faults, and limitations. The potential of AI technologies is not so much the simulation of human intelligence and care-giving but rather an expansion of possibilities through which to realize these human capacities.

This review has identified some clear areas to explore new and novel approaches to human-AI intelligent caring. There are opportunities for innovation (and possible commercial opportunities) to build on and develop (1) better human-AI systems for detecting suffering (e.g., pain, distress, risk, and discrimination) to fine tune AI/human empathetic awareness; (2) use of human-AI intelligence to understand suffering in context; (3) better human-AI verbal and non-verbal communication systems to connect with suffering; (4) human-AI intelligence to inform decisions about the need to act, (5) more authentic and sustainable forms of human-AI empathetic response and interventions; (6) better human-AI intelligence about the effects and outcomes of responses and whether they have alleviated suffering or need to be modified.

In terms of motivation for a human-AI intelligent caring system, it is evident from this literature that AI technologies are helping humans to develop empathetic understanding of human experiences of living with debilitating conditions (Groza et al., 2017; Palanica et al., 2018; Schmidt et al., 2022). Next steps could be to build evidence about how AI technologies might support new ways of connecting (e.g., verbal, physical, signs and symbols) with various forms of suffering (e.g., pain, distress, risk, disadvantage); enable virtual/real proximity such as safe relating (Gilbert, 2021); explore therapeutic alignment (patient preferences for human and virtual providers); or address stigma (e.g., beneficent dehumanization of care). Motivation can be found in examples of AI technologies that are alleviating human suffering; specific use cases identified by this review could be considered humanitarian or miraculous in their effects, such as helping paralyzed people to walk (Sarkar et al., 2020). However, there are more ordinary applications that are nonetheless useful and can build compassion, such as providing the right information to patients at the right time to alleviate distress (Papadakos et al., 2017).

What these findings also highlight is that people are inspired to help each other to help themselves, through the new capabilities of AI technologies. This is evidenced by the development of numerous self-care and self-compassion technologies. There are also entrepreneurial motives (Shepherd and Majchrzak, 2022) which need to be considered in relation to a system of human-AI intelligent caring. Future research could explore the themes of healing spaces and healing relationships to boost self-compassion and self-care in patients and health professionals. There is a need to direct more attention to not only the theory of compassion, but how to use AI technologies to help close the compassion cycle: in other words, how AI technologies can be an important tool for informing and assuring healthcare quality at multiple levels, from individual practitioner, AI technologies, healthcare organizations, to whole healthcare systems–for seeing when responses have made a positive difference to people’s lives or provided motivation to continue to care.

This reconceptualization of compassion aligns with calls to develop compassion as a healthcare system goal and professional development priority (Gray and Cox, 2015; Swendiman et al., 2019). It helps to connect the subjective, experiential, and practical dimensions of compassion (e.g., getting people home safely, organizing transport, “going the extra mile”) with an understanding of how AI technologies might support societal forms of caring (e.g., protecting human rights, advancing health equality) through their individual design and combined effects (Day et al., 2021). A systems perspective of compassion proposes that not everyone or every AI application needs to be delivering empathetic responses in a healthcare system all the time (Bleiker et al., 2020). This is not to say that healthcare professionals should not aim to be “highly humanistic” in their practice (Swendiman et al., 2019), but rather that the system of compassion in healthcare extends beyond human interactions at the individual level. Therefore, compassion can relate not only to direct clinical treatments and patient care but to indirect actions such as the development of AI-driven organizational systems for patient feedback, the use of guidance for use of AI technologies, professional codes of practice for the use of AI technologies, and so on; to employ human health professionals and use AI technologies to best effect within an overall system of intelligent caring.

### 4.4. Implications

In a complex adaptive system such as healthcare, human-AI intelligent caring will need to be implemented, not as an ideology, but through strategic choices, incentives, regulation, professional education, and training, as well as through joined-up thinking about human-AI intelligent caring. *Research funders* internationally in different areas of health, education, and technology research can encourage research and development into the topic of AI technologies and compassion. Interdisciplinary empirical research is needed to explore issues about the educational effectiveness of AI-assisted learning; patient diversity and AI technologies; safety and clinical effectiveness of AI technologies. Theoretically informed research should take a longterm view of how AI technologies can enhance compassion by enriching education, learning and clinical practice; extending healing spaces; and enhancing healing relationships. *Educators* in computing, design sciences, health professional education, and other fields and disciplines of science and humanities, can inform themselves about the association between AI technologies and compassion and promote an understanding of compassion as a human-AI system of intelligent caring involving six elements (see Figure 2). Educators can make use of modern learning technologies to enhance learning engagement, student empathetic awareness, to learn about how to respond to different types of suffering (e.g., pain, distress, risk, and disadvantage), communication skills and teamworking. *Technologists and computer scientists* should be aware that compassion is important and beneficial to human health as well as the sustainability of healthcare systems. They can consider how, in some applications it could be useful to build in artificial empathy (Dial, 2018), or artificial compassion (Mason, 2015, 2023), while in other contexts AI technologies can contribute to specific elements of compassion within healthcare/social systems (e.g., supporting sensitivity to suffering). *Health professionals* can link into interprofessional virtual communities of practice (McLoughlin et al., 2018) to learn and share knowledge of how AI technologies might support compassion in healthcare and to develop the practice of human-AI intelligent caring.

## 5. Conclusion

This systematic scoping review of the literature shows there is an association between AI technologies and compassion in healthcare. Interest in this association has grown internationally over the last decade, with more articles debating the topic and reporting on developments each year. In a range of healthcare contexts, AI technologies are being used to develop or enhance empathetic awareness; empathetic response and relational behavior; communication skills; health coaching; therapeutic interventions; moral development learning; clinical knowledge and clinical assessment; healthcare quality assessment; therapeutic bond and therapeutic alliance; as well as to provide health information and advice. The findings inform a reconceptualization of compassion as a *human-AI system of intelligent caring* comprising six elements. Future research and development into the association between AI technologies and compassion could enrich education, learning, and clinical practice; extend healing spaces; and enhance healing relationships in new and novel ways, made possible by artificial intelligence.

## Author contributions

EM and FR initiated the review and collaborated with CM, TZ, KP, RS, and MR to plan and develop the review focus and approach. EM and TZ undertook the searches. EM wrote the draft. All authors contributed to discussions online (Sept 2021–Jan 2022) and thereafter contributed to conceptualization, thematic analysis, interpretation of the results, and approved the submitted version and commented on the final draft.

## Footnotes

1
https://www.deeplearning.ai/
