# Supplementary material for: Artificial intelligence technologies and compassion in healthcare: A systematic scoping review
Source: Front Psychol. 2023 Jan 17;13:971044. doi: 10.3389/fpsyg.2022.971044 (PMC9887144; doi:10.3389/fpsyg.2022.971044)
Supplement: Supplementary file 1 [file Data_Sheet_1.docx]

**Supplementary Appendix: Literature tables summarizing all of the included articles in the review**

Table notes:

Type of article

I- Systematic review

II- Research studies & study protocol

III- Review of literature/policy/practice

IV- Discussion chapters, commentary, report, perspective or viewpoint

V- Conference paper, workshop, symposia

VI- Design study, proof of concept, service improvement

Country: Affiliation of first author

| **Author/**  **Date ref** | **Reference** | **Type of article** (I-VI) | **Aim/context** (e.g., healthcare issue/setting etc.) | **Key Relevant Findings** (and or implications for practice, design, future research etc.) | **Country** |
| --- | --- | --- | --- | --- | --- |
| **Abdullah et at., 2021** | Abdullah, Y. I., Schuman, J. S., Shabsigh, R., Caplan, A., and Al-Aswad, L. A.  (2021). Ethics of artificial intelligence in medicine and ophthalmology. Asia Pac. J.  Ophthalmol. 10, 289–298. doi: 10.1097/APO.0000000000000397 | III- Literature review | Review of the contemporary literature of the bioethical issues of AI in medicine and ophthalmology, classify ethical issues in medical AI, and suggest possible standardizations of ethical frameworks for AI implementation | - Explores the bioethical implementation of artificial intelligence (AI) in medicine and in ophthalmology. AI, which was first introduced in the 1950s, is defined as "the machine simulation of human mental reasoning, decision making, and behaviour". The increased power of computing, expansion of storage capacity, and compilation of medical big data helped the AI implementation surge in medical practice and research. Ophthalmology is a leading medical specialty in applying AI in screening, diagnosis, and treatment. - Most sources that studied the use of AI in medicine explored the ethical aspects. Bioethical challenges of AI implementation in medicine were categorized into 6 main categories. These include machine training ethics, machine accuracy ethics, patient-related ethics, physician-related ethics, shared ethics, and roles of regulators. - There are multiple stakeholders in the ethical issues surrounding AI in medicine and ophthalmology. Attention to the various aspects of ethics related to AI is important especially with the expanding use of AI. Solutions of ethical problems are envisioned to be multifactorial. | United States |
| **Ajeesh and Rukmini, 2022** | Ajeesh, A. K., and Rukmini, S. (2022). Posthuman perception of artificial  intelligence in science fiction: an exploration of kazuo ishiguro’s klara and the sun.  AI Soc. 22:1533. doi: 10.1007/s00146-022-01533-9 | IV- Discussion | Study to examine the plausibility of the notion of AI as having emotional capabilities, drawing on science fiction writings | - Argues that human fascination with artificial intelligence (AI), robots and sentient machines has a long history, and references to such humanoids are present even in ancient myths and folklore. The advancements in digital and computational technology have turned this fascination into apprehension, with the machines often being depicted as a binary to the human. However, the recent domains of academic enquiry such as transhumanism and posthumanism have produced many a literature in the genre of science fiction (SF) that endeavours to alter this antagonistic notion of AI. - In his novel Klara and the Sun, Kazuo Ishiguro explores this notion of AI as a caring machine capable of nursing an ailing young girl back to health. Through this portrayal of AI as a sentient being capable of empathy and cognition, Ishiguro is attempting to usher in a new perception of AI: a posthuman perception that challenges the conventional notions of AI as a machine devoid of emotions. The novel further expands on the idea of self, soul and human consciousness and ponders on the question, what makes humans human, and if it is possible to imbibe these qualities onto an AI. - Attempts to chart the impact of SF in society and culture. The study reveals a positive shift in perception towards AI, and there seems to be much scope for interdisciplinary and transdisciplinary research. | India |
| **Alameddine et al., 2019** | Alameddine, M., Soueidan, H., Makki, M., Tamim, H., and Hitti, E. (2019). The  use of smart devices by care providers in emergency departments: cross-sectional  survey design. JMIR Mhealth Uhealth. 7:e13614. doi: 10.2196/13614 | II- Research study | Survey study into the use of smart devices (SDs) by health care providers in care settings | - A cross-sectional electronic survey exploring SD use was sent to all ED health care providers (N=236). The target population included core ED faculty members, attending physicians, residents, medical students, and the nursing care providers. A regression model developed in this study was used to find predictors of medical errors in the ED because of the use of SDs. - A total of 83 of 97 respondents (86%) used one or more medical applications on their SDs. 71 out of 87 respondents (82%) believed that using SDs in the ED improved the coordination among the care team, and 71 out of 90 (79%) respondents believed that it was beneficial to patient care. In addition, 37 out of 90 respondents (41%) acknowledged that they were distracted when using their SDs for nonwork purposes. 51 out of 93 respondents (55%) witnessed a colleague committing a near miss or an error owing to the SD-caused distractions. Regression analysis revealed that age (P=.04) and missing information owing to the use of SDs (P=.02) were major predictors of committing an error in the ED. Interestingly, more than 40% of the respondents were significantly addicted to using SDs and more than one-third felt the need to cut down their use. - The findings of this study suggest it is imperative to ensure the safety and wellbeing of patients, especially in high intensity, high volume departments like the ED. Irrespective of the positive role SDs play in the health care process, the negative effects of their use mandate proper regulation, in particular, an ethical mandate that takes into consideration the significant consequences that the use of SDs may have on care processes and outcomes. | Lebanon |
| **Ali et al., 2022** | Ali, S., Kleib, M., Paul, P., Petrovskaya, O., and Kennedy, M. (2022).  Compassionate nursing care and the use of digital health technologies: a scoping  review. Int. J. Nurs. Stud. 127:104161. doi: 10.1016/j.ijnurstu.2021.104161 | III- Literature review | A scoping review was conducted to identify what is known about compassionate care in relation to the use of digital health technologies within the nursing literature | - Twenty-eight articles were included in this review. Narrative results were organised into three themes: 1) evolving understanding of compassionate nursing care in relation to use of digital health technology, 2) compassionate nursing care in relation to the type of digital health technology, and 3) strategies and interventions to improve education and competence relevant to digital health and compassionate nursing care. - The use of technology influences how nurses do their work and interact with patients. As advances in digital health continue to evolve, future research should aim to expand understanding of compassion relevant to digital health by articulating its characteristics and associated competencies for nurses. | Canada |
| **Alrassi et al., 2021** | Alrassi, J., Katsufrakis, P. J., and Chandran, L. (2021). Technology can augment,  but not replace, critical human skills needed for patient care. Acad. Med. 96, 37–43.  doi: 10.1097/ACM.0000000000003733 | IV- Commentary | Artificial intelligence and the changing role of physicians | - The practice of medicine is changing rapidly as a consequence of electronic health record adoption, new technologies for patient care, disruptive innovations that breakdown professional hierarchies, and evolving societal norms. Collectively, these have resulted in the modification of the physician's role as the gatekeeper for health care, increased shift-based care, and amplified interprofessional team-based care. - Artificial intelligence, which has great potential, has already transformed some tasks, particularly those involving image interpretation. Ubiquitous access to information via the Internet by physicians and patients alike presents benefits as well as drawbacks: patients and providers have ready access to virtually all of human knowledge, but some websites are contaminated with misinformation and many people have difficulty differentiating between solid, evidence-based data and untruths. - The role of the future physician will shift as complexity in health care increases and as artificial intelligence and other technologies advance. These technological advances demand new skills of physicians; memory and knowledge accumulation will diminish in importance while information management skills will become more important. In parallel, medical educators must enhance their teaching and assessment of critical human skills (e.g., clear communication, empathy) in the delivery of patient care. - The authors emphasize the enduring role of critical human skills in safe and effective patient care even as medical practice is increasingly guided by artificial intelligence and related technology, and they suggest new and longitudinal ways of assessing essential noncognitive skills to meet the demands of the future. | United States |
| **Amini et al., 2013** | Amini, R., Lisetti, C. L., Yasavur, U., and Rishe, N. (2013). “On-demand  virtual health counselor for delivering behavior-change health interventions,” in  Proceeding of the 2013 IEEE international conference on healthcare informatics,  46–55. doi: 10.1109/ICHI.2013.13 | VI- Design study | Discusses a novel approach for the computer-delivery of Brief Motivational Interventions (BMIs) for health behaviour change | - Describes the basic elements of system architecture and focus on enabling a multimodal Embodied Conversational Agent (ECA) to deliver the health behaviour change interventions empathetically by adapting, in real-time, its verbal and non-verbal communication messages to those of its clients. The designed empathy model integrates a cognitive component and an affective component. - Discusses the evaluation experiment that was designed and conducted to evaluate the impact of empathy model on users' experience with the empathic character. Results indicate that, in comparison with the non-empathic counsellor, the empathic one is better accepted (e.g., more enjoyable, empathizing, engaging, and likable) and some users might be willing to disclose more private information (e.g., drinking habits) to the counsellor endowed with empathic abilities than the one without. | United States |
| **Amini et al., 2021** | Amini, H., Gregory, M. E., Abrams, M. A., Luna, J., Roland, M., Sova, L. N.,  et al. (2021). Feasibility and usability study of a pilot immersive virtual realitybased  empathy training for dental providers. J. Dent. Educ. 85, 856–865. doi:  10.1002/jdd.12566 | VI- Design study | Feasibility and usability study of a pilot immersive virtual reality-based empathy training for dental providers | - Social determinants of health (SDOH) significantly impact individuals' engagement with the healthcare system. To address SDOH-related oral health disparities, providers must be equipped with knowledge, skills, and attitudes (KSAs) to understand how SDOH affect patients and how to mitigate these effects. Traditional dental school curricula provide limited training on recognizing SDOH or developing empathy for those with SDOH-related access barriers. - This study describes the design and evaluation of a virtual reality (VR)-based simulation in dental training to increase post-training knowledge, skills and attitudes. - In the "MPATHI" (Making Professionals Able THrough Immersion) a scripted VR simulation participants take the role of an English-speaking caregiver with limited socioeconomic resources seeking dental care for a child in a Spanish-speaking country. The simulation is a combination of 360 degrees video recording and virtual scenes delivered via VR headsets. A pilot was conducted with 29 dental residents/faculty, utilizing a pre-post design to evaluate effectiveness in improving immediate and retention of KSAs toward care delivery for families facing barriers. - MPATHI led to increased mean scores for cognitive, affective, and skill-based learning immediately post-training. There was not a significant difference between skills measured immediately post-training and in the 1-month post-training survey. Participants reported high satisfaction with the content and methods used in this training. | United States |
| **Andersson et al., 2017** | Andersson, M., Axelsson, K., Fältholm, Y., and Lindberg, I. (2017). Technologies  in older people’s care. Nurs. Ethics 24, 125–137. doi: 10.1177/0969733015594665 | II- Research study | Study to interpret values related to care and technologies connected to the practice of good care for older people | - Explains that the tension between care-based and technology-based rationalities motivates studies concerning how technology can be used in the care sector to support the relational foundation of care. - This research study was part of a development project aimed at developing innovative work practices through information and communication technology. All staff (n = 18) working at two wards in a care facility for older people were asked to participate in interviews, and 12 accepted. - Four values were identified: 'presence', 'appreciation', 'competence' and 'trust'. Caregivers wanted to focus on care receivers as unique persons, a view that they thought was compromised by time-consuming and beeping electronic devices. Appraising from next-of-kin and been seen as someone who can contribute together with knowledge to handle different situations were other desires. The caregivers also desired positive feedback from next-of-kin, as they wanted to be seen as professionals who have the knowledge and skills to handle difficult situations. In addition, the caregivers wanted their employer to trust them, and they wanted to work in a calm environment. - Caregivers' desire for disturbance-free interactions, being valued for their skills and working in a trustful working environment were interpreted as their base for providing good care. The caregivers' arguments are based on caring rationality, and sometimes they felt the technological rationality interfered with their main mission, providing quality care. - Concludes that introducing new technology in caring should support the caring relationship. Although society's overall technology-based approach may have gained popularity as a problem solver, technology-based rationality may compromise a care-based rationality. A shift in attitudes towards care as a concept on all societal levels is needed. | Sweden |
| **Atif et al., 2022** | Atif, N., Nazir, H., Sultan, Z. H., Rauf, R., Waqas, A., Malik, A., et al.  (2022). Technology-assisted peer therapy: a new way of delivering evidence-based  psychological interventions. BMC Health Serv Res. 22:842. doi: 10.1186/s12913-  022-08233-6 | VI- Design study | Technology-assisted peer therapy for delivering evidence-based psychological interventions | - In low-income settings, ninety percent of individuals with clinical depression have no access to evidence-based psychological interventions. Reasons include lack of funds for specialist services, scarcity of trained mental health professionals, and the stigma attached to mental illness. In recent years there have been many studies demonstrating effective delivery of psychological interventions through a variety of non-specialists. While these interventions are cost-effective and less stigmatising, efforts to scale-up are hampered by issues of quality-control, and what has been described by implementation scientists as 'voltage-drop' and 'programme-drift.' - Using principles of Human Centred Design in a rural setting in Pakistan, we worked with potential users to co-design a Tablet or Smartphone-based App that can assist a lay-person deliver the Thinking Healthy Programme, a World Health Organisation-endorsed evidence-based intervention for perinatal depression. The active ingredients of this cognitive-therapy based intervention are delivered by a virtual 'avatar' therapist incorporated into the App which is operated by a 'peer' (a woman from the neighbourhood with no prior experience of healthcare delivery). Using automated cues from the App, the peer reinforces key therapeutic messages, helps with problem-solving and provides the non-specific but essential therapeutic elements of empathy and support. The peer and App therefore act as co-therapists in delivery of the intervention. The peer can deliver the intervention with good fidelity after brief automated in-built training. This approach has the potential to be applied to other areas of mental health and help bridge the treatment gap, especially in resource-poor settings. This paper describes the process of co-development with end-users and key features of the App. | Pakistan |
| **Baghaei et al., 2019** | Baghaei, N., Hach, S., Khaliq, I., Stemmet, L., Krishnan, J., Naslund, J., et al.  (2019). “Increasing self-compassion in young people through virtual reality,” in  Proceeding of the 2019 IEEE international symposium on mixed and augmented  reality adjunct (ISMAR-adjunct), (IEEE), doi: 10.1109/ISMAR-Adjunct.2019.  00042 | VI- Proof of concept study | Early intervention for mental health conditions in young people | - Discussion of feasibility of new technology for the early intervention of mental health and co-design. Virtual Reality scenarios with young people, which focuses on real world situations that impact the sample group most and assists them to view these experiences with a self-compassionate lens. This is achieved by being taught compassionate manners of responding to a scenario and by switching perspective. - Proposes development in different social settings and highlights key points for discussion pertaining to technology use, data safety, privacy, and considerations for addressing depressive symptoms using virtual reality. | United States |
| **Baghaei et al., 2021** | Baghaei, N., Ahmadi, A., Khaliq, I., and Liang, H. (2021). “Individualised virtual  reality for supporting depression: feedback from mental health professionals,” in  Proceeding of the 2021 IEEE international symposium on mixed and augmented  reality adjunct (ISMAR-adjunct), 63–67. doi: 10.1109/ISMAR-Adjunct54149.2021.  00022 | VI- Design study | AI individualised VR technology for mental health therapy | - Discusses iVR, a novel individualised VR experience for enhancing peoples' self-compassion, and in the long run, their mental health, which focuses on real world situations that impact the sample group most and assists them to view these experiences with a self-compassionate lens. This is achieved by being taught compassionate manners of responding to a scenario and by switching perspective. - Seven mental health professionals provided feedback on iVR. They felt that introducing elements of choice within iVR would increase their knowledge of clients. Participants raised issues about technology use, data safety, privacy, and considerations for addressing depressive symptoms. This information can inform large-scale efficacy testing, clinical use, and cost-effective delivery of intelligent individualised VR technology for mental health therapy in future. | New Zealand |
| **Ball et al., 2015** | Ball, S., Bluteau, P., Clouder, D. L., Adefila, A., and Graham, S. (2015).  “MyShoes: an immersive simulation of dementia,” in Proceedings of the  international conference on e-learning, Vol. 2015, (Academic Conferences  Limited.), 16–23. | VI- Design study | myShoes: An immersive simulation of dementia | - The myShoes project aims to promote health care professionals' affective empathy for people with dementia through exposure to an embodied experience. Simulated activities can trigger emotions, such as confusion and frustration caused by the inability to complete simple tasks, process sensory information or do certain tasks repeatedly. Coupling age related visual and auditory overlay filters with misdirection, misperception, object switching and sleight of hand, along with other techniques, can offer an experience not easily accomplished through standard role play. - The project involved the development, testing and piloting of a prototype simulation using an Oculus Rift virtual reality headset. Three learning technologists worked closely with a specialist team consisting of a dementia care expert, a clinical psychologist, a mental health nurse, a physiotherapist and an educational researcher to develop an authentic experience using popular game development tools, including Unity, Blender and 3D assets. - Findings suggest that the project has been successful in simulating a range of aspects of dementia with which students can identify and its applicability in and beyond pre-registration training is being explored. | United Kingdom |
| **Beverly et al., 2022** | Beverly, E., Rigot, B., Love, C., and Love, M. (2022). Perspectives of 360-degree  cinematic virtual reality: interview study among health care professionals. JMIR  Med. Educ. 8:e32657. doi: 10.2196/32657 | VI- Design study | Explores health care professionals' experiences with a cinematic VR (cine-VR) training program focused on role-playing and clinical encounters addressing social determinants of health, Appalachian culture, and diabetes | - Explains that the global market for medical education is projected to increase exponentially over the next 5 years. A mode of delivery expected to drive the growth of this market is virtual reality (VR). VR simulates real-world objects, events, locations, and interactions in 3D multimedia sensory environments. It has been used successfully in medical education for surgical training, learning anatomy, and advancing drug discovery. New VR research has been used to simulate role-playing and clinical encounters; however, most of this research has been conducted with health professions students and not current health care professionals. Thus, more research is needed to explore how health care professionals experience VR with role-playing and clinical encounters. - Cine-VR leverages 360-degree video with the narrative storytelling of cinema to create an engaging educational experience. Telephone interviews with 24 health care professionals who participated in the cine-VR training revealed five themes: immersed in the virtual world: seeing a 360-degree sphere allowed participants to immerse themselves in the virtual world; facilitated knowledge acquisition: all the participants accurately recalled the culture of Appalachia and listed the social determinants of health presented in the training; empathized with multiple perspectives: the cine-VR provided a glimpse into the real life of the main character, and participants described thinking about, feeling, and empathizing with the character's frustrations and disappointments; perceived ease of use of cine-VR: 96% (23/24) of the participants described the cine-VR as easy to use, and they liked the 360-degree movement, image resolution, and sound quality but noted limitations with the buttons on the headsets and risk for motion sickness; and perceived utility of cine-VR as a teaching tool: participants described cine-VR as an effective teaching tool because it activated visual and affective learning for them. - Participants emphasized the realism of the cine-VR training program. They attributed the utility of the cine-VR to visual learning in conjunction with the emotional connection to the VR characters. Furthermore, participants reported that the cine-VR increased their empathy for people. More research is needed to confirm an association between the level of immersion and empathy in cine-VR training for health care professionals. | United States |
| **Bevilacqua et al., 2020** | Bevilacqua, R., Casaccia, S., Cortellessa, G., Astell, A., Lattanzio, F., Corsonello,  A., et al. (2020). Coaching through technology: a systematic review into efficacy  and effectiveness for the ageing population. Int. J. Environ. Res. Public Health  2020:930. doi: 10.3390/ijerph17165930 | I- Systematic review | Review of the efficacy of personal health coaching systems for older adults using digital virtual agents | - Despite the evidence on the positive role of self-management, the adoption of health coaching strategies for older people is still limited. To address these gaps, recent efforts have been made in the ICT sector in order to develop systems for delivering coaching and overcoming barriers relating to scarcity of resources. - Although there was a low number of studies (9), there was evidence that technology-integrated interventions can deliver benefits for health over usual care. However, the review raises important questions about how to maintain benefits and permanence of behaviour change produced by short-term interventions. - These systems offer a potential tool to reduce costs, minimize therapist burden and training, and expand the range of clients who can benefit from them. It is desirable that in the future the number of studies will grow, considering other aspects such as the role of the virtual coaches' characteristics, social-presence, empathy, usability, and health literacy. | Italy |
| **Bjorklund, 2016** | Bjorklund, P. (2016). Gossamer threads: commentary on the impact of digital  technology on the developing brain and the capacity for empathy. ANS Adv. Nurs.  Sci. 39, 71–84. doi: 10.1097/ANS.0000000000000105 | IV- Commentary | Essay explores the impact of digital technology on brain development and other areas of human growth including the capacity for empathy | - Reflections on a teenaged daughter's immersion in technology, coupled with evidence for the mixed consequences of such, weave together the gossamer threads of disparate, sometimes conflicting information about the explosion of digital technology over the past few decades, the impossibility of multitasking, the mixed effects of digital technology on brain development, and what it all might mean for human development generally and the moral capacity for empathic response in particular. - Suggests these perspectives and issues are important for nursing-with its relational core, unique as well as shared bodies of moral and scientific knowledge, and interprofessional health care goals to maximize human growth and well-being across the life span in both health and illness. | United States |
| **Blease et al., 2019** | Blease, C. R., Kaptchuk, T. J., Bernstein, M. H., Mandl, K. D., Halamka, J. D.,  and DesRoches, C. M. (2019). Artificial intelligence and the future of primary care:  exploratory qualitative study of UK general practitioners’ views. J. Med. Int. Res.  21:e12802. doi: 10.2196/12802 | II- Qualitative study | Artificial intelligence and the future of primary care: exploratory qualitative study of UK general practitioners' views | - Web-based survey of 720 UK GPs' opinions about the likelihood of future technology to fully replace GPs in performing 6 key primary care tasks, and, if respondents considered replacement for a particular task likely, to estimate how soon the technological capacity might emerge. - Perceived limitations of future technologies included the beliefs that communication and empathy are exclusively human competencies; many GPs also considered clinical reasoning and the ability to provide value-based care as necessitating physicians' judgments. Perceived benefits of technology included expectations about improved efficiencies, in particular with respect to the reduction of administrative burdens on physicians. Social and ethical concerns encompassed multiple, divergent themes including the need to train more doctors to overcome workforce shortfalls and misgivings about the acceptability of future technology to patients. However, some GPs believed that the failure to adopt technological innovations could incur harms to both patients and physicians. | United Kingdom |
| **Blease et al., 2020** | Blease, C., Locher, C., Leon-Carlyle, M., and Doraiswamy, M. (2020).  Artificial intelligence and the future of psychiatry: qualitative findings from  a global physician survey. Digit Health. 27:2055207620968355. doi: 10.1177/  2055207620968355 | II- Research study | Web-based survey study aimed to explore psychiatrists' opinions about the potential impact innovations in artificial intelligence and machine learning on psychiatric practice | - Survey of 791 psychiatrists from 22 countries worldwide. The survey measured opinions about the likelihood future technology would fully replace physicians in performing ten key psychiatric tasks. - Comments were classified into four major categories in relation to the impact of future technology on: (1) patient-psychiatrist interactions; (2) the quality of patient medical care; (3) the profession of psychiatry; and (4) health systems. - Overwhelmingly, psychiatrists were skeptical that technology could replace human empathy. Many predicted that 'man and machine' would increasingly collaborate in undertaking clinical decisions, with mixed opinions about the benefits and harms of such an arrangement. Participants were optimistic that technology might improve efficiencies and access to care and reduce costs. Ethical and regulatory considerations received limited attention. | United States |
| **Bleiker et al., 2020** | Bleiker, J., Knapp, K., Morgan-Trimmer, S., and Hopkins, S. (2020). What  medical imaging professionals talk about when they talk about compassion. J. Med.  Imag. Radiat. Sci. 51, S44–S52. doi: 10.1016/j.jmir.2020.08.009 | II- Research study | Medical imaging professional’s views on the meaning and practice of compassion | - Compassion is a poorly understood concept in medical Imaging research, but an increase in its focus was recommended in the Francis Report (2013). Little research has been conducted in this area to date. Data were harvested from a Twitter journal club discussion between medical imaging professionals of the author's published literature review and one focus group of post-graduate radiographers. - Results show that compassion in MI is conceptualised according to three themes constructed from the data: 1) Perceptible elements of the procedure; 2) Underlying qualities, skills and abilities of radiographers; 3) Moral and ethical foundations. When medical imaging professionals talk about compassion they talk about its importance in professional practice, the challenges faced in giving compassionate care and the strategies they employ to cope with the emotional as well as physical demands they face. Contradictory organisational values and an over-emphasis on individuals' responsibility for providing compassionate care were also highlighted. Ethical professional practice need not necessarily include in every interaction an expression of compassion, or feelings in a medical imaging professional of caring about their patient. - The concept of compassion has depth, with surface appearances underpinned by moral values and behaviour-motivating drivers. These findings offer a clearer understanding of compassion that could inform radiographic practice and education. | United Kingdom |
| **Boggiss et al., 2022** | Boggiss, A., Consedine, N., Hopkins, S., Silvester, C., Jefferies, C., Hofman, P.,  et al. (2022). A self-compassion chatbot to improve the wellbeing of adolescents  with type 1 diabetes during the COVID-19 pandemic: what do adolescents and  their healthcare professionals want? JMIR Preprints 2022:40641. doi: 10.2196/  preprints.40641 | II- Qualitative study | Evaluation of the acceptability and clinical usability of a novel self-compassion chatbot (called ‘COMPASS’) among adolescents aged 12 to 16 years with type I diabetes and their diabetes healthcare professionals | - Qualitative Zoom interviews exploring views on a newly developed self-compassion chatbot were conducted with 19 adolescents (in 4 focus groups), and 11 diabetes healthcare professionals (in 2 focus groups and 6 individual interviews), - Findings offer early insight into what adolescents with T1D and their healthcare professionals see as advantages of a self-compassion chatbot and desired future additions, such as personalization (mentioned by all 19 adolescents), self-management support (mentioned by 13 of 19 adolescents), clinical utility (mentioned by all 11 healthcare professionals), and breadth and flexibility of tools (mentioned by 10 of 11 healthcare professionals). - The findings suggest that a self-compassion chatbot for adolescents with T1D is acceptable, relevant to common difficulties, and offers clinical utility during the COVID-19 pandemic. However, shared desired features amongst both groups, including problem-solving and integration with diabetes technology to support self-management, creating a safe peer-to-peer sense of community, and broadening the representation of different cultures, lived experience stories, and diabetes challenges, could further improve the potential of the chatbot. | New Zealand |
| **Bouabida et al., 2021** | Bouabida, K., Malas, K., Talbot, A., Desrosiers, M. È, Lavoie, F., Lebouché,  B., et al. (2021). Remote patient monitoring program for COVID-19 patients  following hospital discharge: a cross-sectional study. Front. Digit. Health 3:721044.  doi: 10.3389/fdgth.2021.721044 | II- Research study | Study was to evaluate the capacity and contribution of two different platforms used to remotely monitor patients with COVID-19 to maintain quality, safety, and patient engagement in care, as well as their acceptability, usefulness, and user-friendliness from the user's perspective | - Explains how the COVID-19 pandemic created an urgent need to act to reduce the spread of the virus and alleviate congestion from healthcare services, protect healthcare providers, and help them maintain satisfactory quality and safety of care. Remote COVID-19 monitoring platforms emerged as potential solutions. - The first platform is focused on telecare phone calls (Telecare-Covid) and the second is a telemonitoring app (CareSimple-Covid). - We performed a cross-sectional study. The data were collected through a phone survey from May to August 2020. Data were analyzed using descriptive statistics and t-test analysis. Participants' responses and comments on open-ended questions were analyzed using content analysis to identify certain issues and challenges and potential avenues for improving the platforms. - 51 patients participated in the study. Eighteen participants used the CareSimple-Covid platform and 33 participants used the Telecare-Covid platform. Overall, the satisfaction rate for quality and safety of care for the two platforms was 80%. Over 88% of the users on each platform considered the platforms' services to be engaging, useful, user-friendly, and appropriate to their needs. The survey identified a few significant differences in users' perceptions of each platform: empathy toward users and the quality and safety of the care received were rated significantly higher on the CareSimple-Covid platform than on the Telecare-Covid platform. Users appreciated four aspects of these telehealth approaches: (1) the ease of access to services and the availability of care team members; (2) the user-friendliness of the platforms; (3) the continuity of care provided, and (4) the wide range of services delivered. Users identified some technical limitations and raised certain issues, such as the importance of maintaining human contact, data security, and confidentiality. Improvement suggestions include promoting access to connected devices; enhancing communications between institutions, healthcare users, and the public on confidentiality and personal data protection standards; and integrating a participatory approach to telehealth platform development and deployment efforts. - This study provides preliminary evidence that the two remote monitoring platforms are well-received by users, with very few significant differences between them concerning users' experiences and views. This type of program could be considered for use in a post-pandemic era and for other post-hospitalization clienteles. To maximize efficiency, the areas for improvement and the issues identified should be addressed with a patient-centered approach. | Canada |
| **Brammer et al., 2022** | Brammer, S., Regan, S., Collins, C., and Gillespie, G. (2022). Developing  innovative virtual reality simulations to increase health care providers’  understanding of social determinants of health. J. Contin. Educ. Health Prof. 42,  60–65. doi: 10.1097/CEH.0000000000000400 | II- Research study of virtual reality simulations | Health care provider competency in identifying and addressing social determinants of health (SDH). Aimed to develop two virtual reality simulations (VRSs) as innovative methods to teach HCPs to identify and manage SDH. | - Argues that a curricular gap exists between promoting an understanding of social determinants of health (SDH) and teaching HCPs how to recognize and increase empathy to manage them. - The expert panel found the VRS easy to use, useful as an educational tool, while promoting empathy for patients. Overall, participants were satisfied with using the VRS as an educational experience. - Through VRS technology, this project addresses a curricular gap in HCP training on SDH. VRS can be a useful tool to increase HCPs' understanding of SDH and, potentially, their empathy for patients. | United States |
| **Brandt et al., 2018** | Brandt, C. J., Søgaard, G. I., Clemensen, J., Søndergaard, J., and Nielsen,  J. B. (2018). Determinants of successful ehealth coaching for consumer lifestyle  changes: qualitative interview study among health care professionals. J. Med. Int.  Res. 20:e237. doi: 10.2196/jmir.9791 | II- Qualitative research | Study of how health care professionals perceive eHealth coaching and to explore what influences successful long-term lifestyle change for patients undergoing hybrid eHealth coaching using a collaborative eHealth tool | - Participating health care professionals (n=10) found establishing and maintaining an empathic relationship essential and that asynchronous eHealth lifestyle coaching challenged this compared to face-to-face coaching. The primary reason was that unlike typical in-person encounters in health care, they did not receive immediate feedback from the patients. We identified four central themes relevant to the health care professionals in their asynchronous eHealth coaching: (1) establishing an empathic relationship, (2) reflection in asynchronous eHealth coaching, (3) identifying realistic goals based on personal barriers, and (4) staying connected in asynchronous coaching. - Establishing and maintaining an empathic relationship is probably the most crucial factor for successful subsequent eHealth coaching. It was of paramount importance to get to know the patient first, and the asynchronous interaction aspect presented challenges because of the delay in response times (both ways). It also presented opportunities for reflection before answering. - The health care professionals found they had to provide both relational communication and goal-oriented coaching when using eHealth solutions. Going forward, the quality of the health care professional-patient interaction will need attention if patients are to benefit from collaborative eHealth coaching fully. | Denmark |
| **Bridge and Bridge, 2019** | Bridge, P., and Bridge, R. (2019). Artificial Intelligence in radiotherapy: a  philosophical perspective. J. Med. Imag. Radiat. Sci. 50, S27–S31. doi: 10.1016/j.  jmir.2019.09.003 | IV- Commentary | Explores philosophical perspective on artificial intelligence technologies in radiography | - Suggests that the increasing uptake of machine learning solutions for segmentation and planning leaves no doubt that artificial intelligence (AI) will soon be providing input into a range of radiotherapy procedures. Although this promises to deliver increased speed and accuracy, the future role of AI in relation to radiotherapy should be thought through carefully. There is currently a gap between published developments and widespread adoption, which provides some space to prepare the workforce and to consider the implications on practice. It is rare to find philosophical input into a medical journal, but the advent of AI makes this perspective increasingly important. - Philosophical insight can help explore the potential impact of AI, in particular, on human creativity and oversight. Without this perspective, we run the risk of focusing solely on the immediate logistical impact on patients and departments. This commentary identifies three key aspects of radiotherapy that the authors feel would suffer most under AI control: creativity, innovation, and patient safety, which all demand uniquely human attributes. - The article provides insight from a philosophical perspective with regard to human consciousness, ethics, and empathy. Philosophically we should, perhaps, retain ethical concerns about the widening role of AI in radiotherapy beyond simple quantitative interpretation and image processing. As developments continue, we have time to determine how our roles will evolve and to establish a framework for ensuring appropriate human input into patient care. Most importantly, we must start to embed a philosophical approach to adoption of AI technology from the outset if we are to prepare ourselves for the challenge that lies ahead. | United Kingdom |
| **Broadbent et al., 2018** | Broadbent, E., Johanson, D., and Shah, J. (2018). “A new model to enhance  robot-patient communication: applying insights from the medical world,” in Social  robotics - 10th international conference, ICSR 2018, qingdao, china, eds S. G.  Shuzhi, J. J. Cabibihan, M. A. Salichs, E. Broadbent, H. He, and A. R. Wagner.  doi: 10.1007/978-3-030-05204-1_30 | III- Review | Socially assistive robots in healthcare applications | - Socially assistive robots need to be able to communicate effectively with patients in healthcare applications. This paper outlines research on doctor-patient communication and applies the principles to robot-patient communication. Effective communication skills for physicians include information sharing, relationship building, and shared decision making. Little research to date has systematically investigated the components of physician communication skills as applied to robots in healthcare domains. - Proposes a new model of robot-patient communication and puts forward a research agenda for advancing knowledge of how robots can communicate effectively with patients to influence health outcomes. | New Zealand |
| **Brydon et al. 2021** | Brydon, M., Kimber, J., Sponagle, M., MacLaine, J., Avery, J., Pyke, L., et al.  (2021). Virtual reality as a tool for eliciting empathetic behaviour in carers: an  integrative review. J. Med. Imag. Radiat. Sci. 52, 466–477. doi: 10.1016/j.jmir.2021.  04.005 | III- Integrative review | Virtual reality as a tool for eliciting empathetic behaviour in carers | - Seven studies, with 485 healthcare providers carers, were included in this review. Studies were heterogeneous in terms of interventions and tools for collecting outcome measures. All seven studies demonstrated VR could elicit empathetic behaviour in carers. Studies included one randomized controlled trial, three non-randomized controlled trials, two with quasi-experimental designs, and one non-experimental design. All studies had a moderate to high risk of bias. Suggests that VR may be an appropriate method for eliciting empathetic behaviours in professional carers. Future studies employing appropriately powered multicentre randomized controlled designs should seek to determine which VR experiences are the most effective in evoking empathetic behaviours. | Canada |
| **Buchanan et al., 2020** | Buchanan, C., Howitt, M. L.,Wilson, R., Booth, R. G., Risling, T., and Bamford,  M. (2020). Predicted influences of artificial intelligence on the domains of nursing:  scoping review. JMIR Nurs. 3:e23939. doi: 10.2196/23939 | III- Scoping review | Scoping review of how emerging trends in artificial intelligence-driven digital health technologies may influence the relationship between nurses and patients | - This review aims to summarize the extant literature on the emerging trends in health technologies powered by AI and their implications on the following domains of nursing: administration, clinical practice, policy, and research. This review summarizes the findings from 3 research questions, examining how these emerging trends might influence the roles and functions of nurses and compassionate nursing care over the next 10 years and beyond. - Emerging AI technologies discussed in the review included predictive analytics, smart homes, virtual health care assistants, and robots. The results indicated that AI has already begun to influence nursing roles, workflows, and the nurse-patient relationship. In general, robots are not viewed as replacements for nurses. There is a consensus that health technologies powered by AI may have the potential to enhance nursing practice. Consequently, nurses must proactively define how person-centered compassionate care will be preserved in the age of AI. - Argues that nurses have a shared responsibility to influence decisions related to the integration of AI into the health system and to ensure that this change is introduced in a way that is ethical and aligns with core nursing values such as compassionate care. Nurses must advocate for patient and nursing involvement in all aspects of the design, implementation, and evaluation of these technologies. | Canada |
| **Buijs-Spanjers et al., 2019** | Buijs-Spanjers, K. R., Hegge, H. H. M., Cnossen, F., Hoogendoorn, E., Jaarsma,  D. A. D. C., and de Rooij, S. E. (2019). Dark play of serious games: effectiveness  and features (G4HE2018). Games Health J. 8, 301–306. doi: 10.1089/g4h.2018.  0126 | VI- Design study | Exploring dark play in game-based learning | - Choosing inappropriate or unethical actions in games is referred to as dark play. For a serious game on delirium for medical students, this study aimed to investigate the potential differences between dark play and normal play on game effectiveness regarding abilities in advising care, learning motivation and engagement, and attitude toward delirious patients. Furthermore, the study aimed to explore the use of different game features between the two types of play on empathy, self-efficacy, and consequences of care. Two-arm randomized controlled trial including an exploratory qualitative approach with 157 medical students, who played the serious game "The Delirium Experience." Participants were randomly allocated to either the dark play or normal play group. Participants had to give three recommendations for taking care of delirious patients, and complete both the Delirium Attitude Scale, and Learning Motivation and Engagement Questionnaire to study game effectiveness. To explore game features, open questions were asked. Results: We did not find difference between the two types of play in game effectiveness. - "Patient's and nurse's perspective" seem to be an important game feature for being able to empathize with a patient in both groups. To support self-efficacy, "practice how to care" and "feedback in the game" were important in both study groups. "Being able to see the importance of good interaction with the patient" was reported important for self-efficacy in the dark play group, whereas this was "seeing the consequences of care" in the normal play group. - There seems to be no change to game effectiveness when providing players the opportunity to use dark play in a serious game. A realistic view of another person's perspective could be an important game feature to increase empathy. | Netherlands |
| **Chew and Achananuparp, 2022** | Chew, H. S. J., and Achananuparp, P. (2022). Perceptions and needs of artificial  intelligence in health care to increase adoption: scoping review. J. Med. Int. Res.  24:e32939. doi: 10.2196/32939 | III- Scoping review | Overview of the perceptions and needs of AI to increase its adoption in health care | - The perceptions and needs of various populations in the use of AI were identified for general, primary, and community health care; chronic diseases self-management and self-diagnosis; mental health; and diagnostic procedures. - The use of AI was perceived to be positive because of its availability, ease of use, and potential to improve efficiency and reduce the cost of health care service delivery. However, concerns were raised regarding the lack of trust in data privacy, patient safety, technological maturity, and the possibility of full automation. - Suggestions for improving the adoption of AI in health care were highlighted: enhancing personalization and customizability; enhancing empathy and personification of AI-enabled chatbots and avatars; enhancing user experience, design, and interconnectedness with other devices; and educating the public on AI capabilities. Several corresponding mitigation strategies were also identified by the review. - Points highlighted in this study could enhance the acceptability and adoption of AI in health care and facilitate an increase in the effectiveness and efficiency of health care service delivery to improve patient outcomes and satisfaction. | Singapore |
| **Clavelle et al., 2019** | Clavelle, J. T., Sweeney, C. D., Swartwout, E., Lefton, C., and Guney, S. (2019).  Leveraging technology to sustain extraordinary care: a qualitative analysis of  meaningful nurse recognition. J. Nurs. Administr. 49, 303–309. doi: 10.1097/NNA.  0000000000000757 | II- Research study | Using AI techniques to analyse nurse recognition comments | - Meaningful recognition of nurses submitted by patients and families using interactive patient care (IPC) technology was analysed using artificial intelligence (AI) to identify the themes and behaviours associated with extraordinary nursing. Meaningful recognition positively impacts nursing and organisational outcomes. The use of AI techniques such as natural language processing and machine learning to identify and describe behaviours impacting patient experiences is an emerging science. - Nurse recognition comments were collected from a convenience sample of 3 organisations via an IPC inpatient platform and analysed using the AI techniques of natural language processing, machine learning, sentiment analytics, and corollary dictionaries based on rules of linguistics. - The top theme of nursing recognition comments was courtesy and respect with the behaviours of empathy/compassion, helpfulness, kindness, attentiveness, and emotional comfort. The theme of skills/knowledge was the 2nd most common, with the behaviours of being professional, knowledgeable, keeping track, competence, dedication, and being thorough. - AI techniques for qualitative analysis of comments collected through IPC reveal nurse themes and behaviours most meaningful to patients and their family members. Nurses can advance the science of AI and guide its evolution so that nurse caring behaviours associated with establishing human connections that positively influence patient and family experience are accurately represented. | United States |
| **Combs and Combs, 2019** | Combs, C. D., and Combs, P. F. (2019). Emerging roles of virtual patients in the  age of AI. AMA J. Ethics 21, E153–E159. doi: 10.1001/amajethics.2019.153 | III- Literature review | Reviews literature on virtual patients (VPs) and their underlying virtual reality technology, examines VPs' potential through the example of psychiatric intake teaching, and identifies promises and perils posed by VP use in medical education | - Suggests that today's web-enabled and virtual approach to medical education is different from the 20th century's Flexner-dominated approach. Now, lectures get less emphasis, and more emphasis is placed on learning via early clinical exposure, standardized patients, and other simulations. - Identifies key questions: What is the danger of VPs providing incorrect feedback? Who is responsible when the feedback is flawed? What is the potential for the malicious use of VPs? Will VPs diminish in-person interactions among teachers and learners? What impact does the growing use of VPs have on teaching and learning? | United States |
| **Daher et al., 2020** | Daher, K., Casas, J., Abou Khaled, O., and Mugellini, E. (2020). “Empathic  chatbot response for medical assistance. assoc comp machinery,” in Proceedings  of the 20th ACM international conference on intelligent virtual agents (ACM IVA  2020), doi: 10.1145/3383652.3423864 | VI- Design study | Study to examine empathic chatbot response for medical assistance | - Design and building of two different medical assistant chatbots with the goal of providing a diagnosis for physical health problem to the user based on a short conversation. One chatbot was advice-only and asked only the necessary questions for the diagnosis without responding to the user's emotions. Another chatbot, capable of showing empathy, responded in a more supportive manner by analyzing the user's emotions and generating appropriate responses with a high empathic accuracy. Using the RoPE scale questionnaire for empathy perception in a human-robot interaction, the empathic chatbot was rated significantly better in showing empathy and was preferred by a majority of the preliminary study participants (N=12). | Switzerland |
| **Darcy et al., 2021** | Darcy, A., Daniels, J., Salinger, D.,Wicks, P., and Robinson, A. (2021). Evidence  of human-level bonds established with a digital conversational agent: crosssectional,  retrospective observational study. JMIR Format. Res. 5:e27868. doi: 10.  2196/27868 | II- Research study | Study to investigate whether users of a cognitive behavioural therapy (CBT)-based conversational agent would report therapeutic bond levels that are similar to those in literature about other CBT modalities, including face-to-face therapy, group CBT, and other digital interventions that do not use a conversational agent | - Asserts that there are far more patients in mental distress than there is time available for mental health professionals to support them. Although digital tools may help mitigate this issue, critics have suggested that technological solutions that lack human empathy will prevent a bond or therapeutic alliance from being formed, thereby narrowing these solutions' efficacy. - A cross-sectional, retrospective study design was used to analyse aggregate, deidentified data from adult users who self-referred to a CBT-based, fully automated conversational agent (Woebot) between November 2019 and August 2020. Working alliance was measured with the Working Alliance Inventory-Short Revised (WAI-SR), and depression symptom status was assessed by using the 2-item Patient Health Questionnaire (PHQ-2). All measures were administered by the conversational agent in the mobile app. WAI-SR scores were compared to those in scientific literature abstracted from recent reviews. - Data from 36,070 Woebot users were included in the analysis. Participants ranged in age from 18 to 78 years, and 57.48% (20,734/36,070) of participants reported that they were female. The mean PHQ-2 score was 3.03 (SD 1.79), and 54.67% (19,719/36,070) of users scored over the cutoff score of 3 for depression screening. Within 5 days of initial app use, the mean WAI-SR score was 3.36 (SD 0.8) and the mean bond subscale score was 3.8 (SD 1.0), which was comparable to those in recent studies from the literature on traditional, outpatient, individual CBT and group CBT (mean bond subscale scores of 4 and 3.8, respectively). PHQ-2 scores at baseline weakly correlated with bond scores (r=-0.04; P<.001); however, users with depression and those without depression had high bond scores of 3.45. - Concludes that although bonds are often presumed to be the exclusive domain of human therapeutic relationships, these findings challenge the notion that digital therapeutics are incapable of establishing a therapeutic bond with users. Future research might investigate the role of bonds as mediators of clinical outcomes, since boosting the engagement and efficacy of digital therapeutics could have major public health benefits. | United States |
| **Davenport and Kalakota, 2019** | Davenport, T., and Kalakota, R. (2019). The potential for artificial intelligence  in healthcare. Fut. Health. J. 6, 94–98. doi: 10.7861/futurehosp.6-2-94 | IV- Commentary | Explores the future potential of AI in healthcare | - Asserts that the complexity and rise of data in healthcare means that artificial intelligence (AI) will increasingly be applied within the field. Several types of AI are already being employed by payers and providers of care, and life sciences companies. The key categories of applications involve diagnosis and treatment recommendations, patient engagement and adherence, and administrative activities. Although there are many instances in which AI can perform healthcare tasks as well or better than humans, implementation factors will prevent large-scale automation of healthcare professional jobs for a considerable period. Ethical issues in the application of AI to healthcare are also discussed. | United States |
| **Day et al., 2021** | Day, J., Finkelstein, J. C, Field, B. A, Matthews, B., Kirby, J. N, and Doty, J. R  (2021). Compassion-focused technologies: reflections and future directions. Front.  Psychol. 12:603618. doi: 10.3389/fpsyg.2021.603618 | IV- Perspective | Discusses how compassion relates to the context of modern technology | - Describes compassion as a prosocial motivation that is critical to the development and survival of the human species. Cultivating compassion involves developing deep wisdom, insight, and understanding into the nature and causes of human suffering; and wisdom and commitment to take positive action to alleviate suffering. While advances in digital technology build on humankind's vast capacity to develop practical tools that promise to enrich our lives and improve our social connections, in reality the effects are often far from benign. - Explains the motives underlying the development of many contemporary digital platforms seem rooted in competitiveness and capitalism, while modern social media and online platforms are having a profound and pervasive impact on the mental health and wellbeing of humans around the globe. Nonetheless, digital technology holds considerable potential to promote compassionate insight, wisdom, and prosocial behaviour. - Reflects on the current state of technology within human society and examines the notion of compassionate technologies; discusses how contemporary paradigm shifts such as the inclusive design movement may be harnessed to build tools and platforms that promote collective good and increase prosocial behaviour; and highlight examples of initiatives that are harnessing modern technology to advance democracy, collective knowledge, and personal freedoms and agency. | Australia |
| **Dean et al., 2020** | Dean, S., Halpern, J., McAllister, M., and Lazenby, M. (2020). Nursing  education, virtual reality and empathy? Nurs. Open. 7, 2056–2059. doi: 10.1002/  nop2.551 | IV- Commentary | An empathic approach to patient-centred care using virtual reality simulations in nursing education | - This paper posits some questions, does it simply reinforce a 'type' of patient, neglecting caring for the patient as unique, is empathy what results or is it pity, does it result in a greater distance being created between the patient and the health care provider? Can we ever really know what it is like to walk in a patient's shoes when what we experience through virtual reality provides a small snapshot of the vicissitudes of living with an illness or disability. - The authors suggest that what matters most in simulations using virtual reality is how the student exits the experience and if they leave knowing just what patients 'like that' feel, or whether they leave with humility and curiosity. | Australia |
| **Demarinis, 2022** | Demarinis, S. (2022). Healthcare providers use virtual reality to elicit empathy.  Exp. J. Sci. Healing 18:1550. | III- Review | Healthcare providers use of virtual reality to elicit empathy | - Studies investigating VR experiences ranged from a single eight-minute session to sessions lasting 20-25 minutes in duration delivered on two separate days, both in immersive VR environments where participants assumed the role of a care recipient and non-immersive VR environments where the participants assumed the role of a care provider in a simulated care setting. The two types of studies helped gain an understanding of what it is like to have a specific disease or need and to practice interacting with virtual care recipients. | Canada |
| **Doing-Harris et al., 2017** | Doing-Harris, K., Mowery, D. L., Daniels, C., Chapman, W. W., and Conway,  M. (2017). Understanding patient satisfaction with received healthcare services: a  natural language processing approach. AMIA Ann. Symp. Proc. 2016, 524–533. | V- Symposia | Uses natural language processing of free-text patient comments to identify the most frequent reasons for patient satisfaction and dissatisfaction | - Explains how important information is encoded in free-text patient comments. Determines the most common topics in patient comments, design automatic topic classifiers, identify comments ' sentiment, and find new topics in negative comments. The annotation scheme consisted of 28 topics, with positive and negative sentiment. Within those 28 topics, the seven most frequent accounted for 63% of annotations. For automated topic classification, vocabulary-based and Naive Bayes ' classifiers were developed. For sentiment analysis, another Naive Bayes ' classifier was used. Topic modelling was used to search for unexpected topics within negative comments. - The seven most common topics were appointment access, appointment wait, empathy, explanation, friendliness, practice environment, and overall experience. The best F-measures from our classifier were 0.52(NB), 0.57(NB), 0.36(Vocab), 0.74(NB), 0.40(NB), and 0.44(Vocab), respectively. F- scores ranged from 0.16 to 0.74. The sentiment classification F-score was 0.84. Negative comment topic modelling revealed complaints about appointment access, appointment wait, and time spent with physician. | United States |
| **Doraiswamy et al., 2020** | Doraiswamy, P. M., Blease, C., and Bodner, K. (2020). Artificial intelligence and  the future of psychiatry: insights from a global physician survey. Artif Intell Med.  102:101753. doi: 10.1016/j.artmed.2019.101753 | II- Survey research | Study of the global psychiatrist community's opinion regarding the potential of future autonomous technology (AI/ML) to replace key tasks carried out in mental health practice | - Respondents were 791 psychiatrists from 22 countries representing North America, South America, Europe and Asia-Pacific. Documenting and updating medical records (75 %) and synthesizing information (54 %) were the two tasks where a majority predicted that AI/ML could fully replace human psychiatrists. Female- and US-based doctors were more uncertain that the benefits of AI would outweigh risks than male- and non-US doctors, respectively. Around one in 2 psychiatrists did however predict that their jobs would be substantially changed by AI/ML. | United States |
| **Eagle et al., 2022** | Eagle, T., Blau, C., Bales, S., Desai, N., Li, V., and Whittaker, S. (2022). “I  don’t know what you mean by i am anxious”: a new method for evaluating  conversational agent responses to standardized mental health inputs for anxiety  and depression. ACM Trans. Int. Intelli. Syst. 12, 1–23. doi: 10.1145/3488057 | VI- Design study | Evaluation of the effectiveness of conversational agents for advice about anxiety and depression | - Conversational agents (CAs) are increasingly ubiquitous and are now commonly used to access medical information. However, we lack systematic data about the quality of advice such agents provide. - Defines a new method to systematically evaluate mental health responses from conversational agents (Cas). Multi-utterance conversational probes derived from two widely used mental health diagnostic surveys, the PHQ-9 (Depression) and the GAD-7 (Anxiety). The study evaluates the responses of two text-based chatbots and four voice assistants to determine whether CAs provide relevant responses and treatments. Evaluations were conducted both by clinicians and immersively by trained raters, yielding consistent results across all raters. - Although advice and recommendations were generally low quality, they were better for Crisis probes and for probes concerning symptoms of Anxiety rather than Depression. Responses were slightly improved for text versus speech-based agents, and when CAs had access to extended dialogue context. Design implications include suggestions for improved responses through clarification sub-dialogues. Responses may also be improved by the incorporation of empathy although this needs to be combined with effective treatments or advice. | United States |
| **Esmaeilzadeh et al., 2021** | Esmaeilzadeh, P., Mirzaei, T., and Dharanikota, S. (2021). Patients’ perceptions  toward human-artificial intelligence interaction in health care: experimental study.  J. Med. Int. Res. 23:e25856. doi: 10.2196/25856 | II-Experimental study | Study to explore how patients perceive the benefits, risks, and use of AI clinical applications for their health care purposes and how their perceptions may be different if faced with three health care service encounter scenarios | - Experiment that crossed a type of health condition (ie, acute or chronic) with three different types of clinical encounters between patients and physicians (ie, AI clinical applications as substituting technology, AI clinical applications as augmenting technology, and no AI as a traditional in-person visit). An online survey was used to collect data from 634 individuals. - The interactions between the types of health care service encounters and health conditions significantly influenced individuals' perceptions of privacy concerns, trust issues, communication barriers, concerns about transparency in regulatory standards, liability risks, benefits, and intention to use across the six scenarios. There were no significant differences among scenarios regarding perceptions of performance risk and social biases. - The results imply that incompatibility with instrumental, technical, ethical, or regulatory values can be a reason for rejecting AI applications in health care. Thus, there are still various risks associated with implementing AI applications in diagnostics and treatment recommendations for patients with both acute and chronic illnesses. The concerns are also evident if the AI applications are used as a recommendation system under physician experience, wisdom, and control. | United States |
| **Falconer et al., 2014** | Falconer, C. J., Slater, M., Rovira, A., King, J. A., Gilbert, P., Antley, A.,  et al. (2014). Embodying compassion: a virtual reality paradigm for overcoming  excessive self-criticism. PLoS One 9:e111933. doi: 10.1371/journal.pone.0111933 | VI- Design study | A virtual reality paradigm for overcoming excessive self-criticism | - Explains that virtual reality has been successfully used to study and treat psychological disorders such as phobias and posttraumatic stress disorder but has rarely been applied to clinically-relevant emotions other than fear and anxiety. Self-criticism is a ubiquitous feature of psychopathology and can be treated by increasing levels of self-compassion. - Exploited the known effects of identification with a virtual body to arrange for healthy female volunteers high in self-criticism to experience self-compassion from an embodied first-person perspective within immersive virtual reality. Whereas observation and practice of compassionate responses reduced self-criticism, the additional experience of embodiment also increased self-compassion and feelings of being safe. The results suggest potential new uses for immersive virtual reality in a range of clinical conditions. | United Kingdom |
| **Fernandez-Luque & Imran, 2018** | Fernandez-Luque, L., and Imran, M. (2018). Humanitarian health computing  using artificial intelligence and social media: a narrative literature review. Int. J.  Med. Inform. 114, 136–142. doi: 10.1016/j.ijmedinf.2018.01.015 | III- Review | Review of cases of AI applications in humanitarian health crisis | - Successful case studies of AI applications in a humanitarian health crisis have been reported, such as for outbreak detection. A commonly shared concern in the reviewed literature is the technical challenge of analysing large amounts of data in real time. Data interoperability, which is essential to data sharing, is also a barrier with regard to the integration of online and traditional data sources. Human and organisational aspects that might be key factors for the adoption of AI and social media remain understudied. There is also a publication bias toward high-income countries. - The feasibility of using AI to extract valuable information during a humanitarian health crisis is proven in many cases. There is a lack of research on how to integrate the use of AI into the work-flow and large-scale deployments of humanitarian aid during a health crisis. | Qatar |
| **Flemotomos et al., 2022** | Flemotomos, N.,Martinez, V. R., Chen, Z., Singla, K., Ardulov, V., Peri, R., et al.  (2022). Automated evaluation of psychotherapy skills using speech and language  technologies. Behav. Res. Methods 54, 690–711. doi: 10.3758/s13428-021-01623-4 | VI- Design study | Automated evaluation of psychotherapy skills using speech and language technologies | - With the growing prevalence of psychological interventions, it is vital to have measures which rate the effectiveness of psychological care to assist in training, supervision, and quality assurance of services. Traditionally, quality assessment is addressed by human raters who evaluate recorded sessions along specific dimensions, often codified through constructs relevant to the approach and domain. This is, however, a cost-prohibitive and time-consuming method that leads to poor feasibility and limited use in real-world settings. This study developed an automated competency rating tool able to process the raw recorded audio of a session, analysing who spoke when, what they said, and how the health professional used language to provide therapy. Focusing on a use case of a specific type of psychotherapy called "motivational interviewing", the system gives comprehensive feedback to the therapist, including information about the dynamics of the session (e.g., therapist's vs. client's talking time), low-level psychological language descriptors (e.g., type of questions asked), as well as other high-level behavioral constructs (e.g., the extent to which the therapist understands the clients' perspective). - Widespread use of automated psychotherapy rating tools may augment experts' capabilities by providing an avenue for more effective training and skill improvement, eventually leading to more positive clinical outcomes. | United States |
| **Fleury-Perkins & Paris, 2019** | Fleury-Perkins, C., and Paris, M. (2019). L’intelligence artificielle, réflexion  philosophique [artificial intelligence, philosophical reflection]. Soins Revue  Reference Infirm. 64, 24–27. doi: 10.1016/j.soin.2019.05.002 | IV- Commentary | Philosophical reflection on artificial technologies | - Highlights that the use of artificial intelligence and robotics in health care means ethical principles need to be established. Artificial and human intelligence must be implemented in such a as way as to complement each other. From humanism to anthropotechnics, the definitions of human and humanism are not set in stone. A philosophical reflection can enable their definition to be shaped. | France |
| **Francis et al., 2018** | Francis, K. B., Gummerum, M., Ganis, G., Howard, I. S., and Terbeck, S. (2018).  Virtual morality in the helping professions: simulated action and resilience. Br. J.  Psychol. 109, 442–465. doi: 10.1111/bjop.12276 | II- Research study | Study using virtual technologies to explore occupation and moral decision-making in service professionals | - Highlights that recent advances in virtual technologies have allowed the investigation of simulated moral actions in aversive moral dilemmas. Previous studies have employed diverse populations to explore these actions, with little research considering the significance of occupation on moral decision-making. This - Study investigated simulated moral actions in virtual reality made by professionally trained paramedics and fire service incident commanders who are frequently faced with and must respond to moral dilemmas. - Specially trained individuals showed distinct empathic and related personality trait scores and that these declined with years of experience working in the profession. Supporting the theory that these professionals develop resilience in moral conflict, reduced emotional arousal was observed during virtual simulations of a distressing dilemma. Trained professionals demonstrated less regret following the execution of a moral action in virtual reality when compared to untrained control populations. Contrary to previous research, trained individuals made the same moral judgements and moral actions as untrained individuals, though showing less arousal and regret. In the face of increasing concerns regarding empathy decline in health care professionals, these findings suggest that the nature of this decline is complex and likely reflects the development of a necessary emotional resilience to distressing events. | United Kingdom |
| **Fritzsche et al., 2021** | Fritzsche, H., Barbazzeni, B., Mahmeen, M., Haider, S., and Friebe, M. (2021).  A structured pathway toward disruption: a novel healthtec innovation design  curriculum with entrepreneurship in mind. Front. Public Health 9:715768. doi:  10.3389/fpubh.2021.715768 | II- Research study | Explains the development and components of a novel health technologies innovation design curriculum | - Argues that the typical curriculum of training and educating future clinicians, biomedical engineers, health IT, and artificial intelligence experts lacks needed twenty first-century skills like problem-solving, stakeholder empathy, curiosity stimulation, entrepreneurship, and health economics, which are essential generators and are pre-requirements for creating intentional disruptive innovations. Moreover, the translation from research to a valuable and affordable product/process innovation is not formalized by the current teachings that focus on short-term rather than long-term developments, leading to inaccurate and incremental forecasting on the future of healthcare and longevity. - The Stanford Biodesign approach of unmet clinical need detection would be an excellent starting methodology for health-related innovation work, although unfortunately not widely taught yet. We have developed a novel lecture titled HealthTec Innovation Design (HTID) offered in an interdisciplinary setup to medical students and biomedical engineers. It teaches a future-oriented view and the application and effects of exponential trends. We implemented a novel approach using the Purpose Launchpad meta-methodology combined with other innovation generation tools to define, experiment, and validate existing project ideas. As part of the process of defining the novel curriculum, we used experimentation methods, like a global science fiction event to create a comic book with Future Health stories and an Innovation Think Tank Certification Program of a large medical technology company that is focused on identifying future health opportunities. - Concludes that these initiatives were impactful in developing an innovative design thinking approach. Participants' awareness and enthusiasm were raised, including their willingness to implement taught skills, values, and methods in their working projects. A new curriculum based on HTID is essential and needed to move the needle of healthcare activities from treating sickness to maintaining health. | Germany |
| **Gallos et al., 2022** | Gallos, P., Menychtas, A., Panagopoulos, C., Kaselimi, M., Temenos, A., Rallis,  I., et al. (2022). Using mHealth technologies to promote public health and wellbeing  in urban areas with blue-green solutions. Stud. Health Technol. Inform. 295,  566–569. doi: 10.3233/SHTI220791 | VI- Design study | Presents the specifications, the design and the development of a mobile application (mHealth) which collects health-related and location data of users visiting areas with Blue-Green Solutions | - Asserts that European and International cities face crucial global geopolitical, economic, environmental, and other changes. All these intensify threats to and inequalities in citizens' health. The implementation of Blue-Green Solutions in urban and rural areas have been broadly used to tackle the above challenges. The Mobile health (mHealth) technologies contribution in people's well-being has found to be significant. In addition, several mHealth applications have been used to support patients with mental health or cardiovascular diseases with very promising results. The patients' remote monitoring can be a valuable asset in chronic diseases management for patients suffering from diabetes, hypertension or arrhythmia, depression, asthma, allergies and others. - A mobile application has been developed to record the citizens' and patients' physical activity and vital signs using wearable devices. The proposed application can also monitor patients physical, physiological, and emotional status as well as motivate them to engage in social and self-caring activities. Additional features include the analysis of the patients' behaviour to improve self-management. The "HEART by BioAsssist" application could be used as a health and other data collection tool as well as an "intelligent assistant" to monitor and promote patient's physical activity. | Greece |
| **Gavarkovs, 2019** | Gavarkovs, A. G. (2019). Behavioral counseling training for primary care  providers: immersive virtual simulation as a training tool. Front. Public Health  7:116. doi: 10.3389/fpubh.2019.00116 | VI- Design study | Study of behavioural counselling training for primary care providers: immersive virtual simulation as a training tool | - Behavioral counselling represents an efficacious approach for improving health behaviours on a population level, and the primary care setting is an appropriate context in which to implement this approach. However, evidence suggests that the utilization of behavioural counselling techniques in primary care, including those informed by motivational interviewing, is sub-optimal. Insufficient training has been cited as a barrier to utilizing counselling in the primary care setting. Recent work has evaluated the effectiveness of virtual simulations that can provide access to "virtual" patients while retaining the scalability inherent to a digital medium. - However, these educational interventions have been limited to simulations delivered through a two-dimensional screen. More immersive simulations delivered through a head-mounted display can create a realistic practice environment that encompasses a learner's entire field of view, which may confer additional benefits with respect to training outcomes. The purpose of this short article is to briefly review the relevant literature across disciplines to conceptualize the potential effectiveness of this technology as a training tool for behavioural counselling. Immersive virtual simulations are designed to induce a psychological phenomenon referred to as presence, whereby a learner perceives themselves as existing within the virtual environment. As such, immersive virtual simulations can provide opportunities for practice, coaching, and feedback in an environment that closely approximates the clinical setting in which counselling will be delivered. Through its effects on presence, this technology may be particularly useful for developing empathy, which is an important component of counselling. | United States |
| **Giambattista et al., 2016** | Giambattista, A., Teixeira, L., Ayanoglu, H., Saraiva, M., and Duarte, E. (2016).  “Expression of emotions by a service robot: a pilot study,” in Design, user  experience, and usability: technological contexts. DUXU 2016, Vol. 9748, ed. A.  Marcus (Cham: Springer), doi: 10.1007/978-3-319-40406-6_31 | II- Pilot study | Expression of emotions by a service robot | - Examined the recognition of emotions being expressed by a service robot in a virtual environment (VE), by university students. The robot's facial expressions, body movements, and displacement were manipulated to express eight basic emotions. Results showed that participants had difficulties in recognizing the emotions (33% of success). Also, results suggested that the participants established empathy with the robot. Further work is needed to improve the emotional expression of this robot, which aims to interact with hospitalized children. | Italy |
| **Gillespie et al., 2021** | Gillespie, G. L., Farra, S., Regan, S. L., and Brammer, S. V. (2021). Impact  of immersive virtual reality simulations for changing knowledge, attitudes, and  behaviors. Nurse Educ. Today 105:105025. doi: 10.1016/j.nedt.2021.105025 | II- Research study | Impact of immersive virtual reality simulations for changing knowledge, attitudes, and behaviours | - Study with 206 healthcare workers who completed a virtual reality simulation identified themes relating to (1) Acknowledgement of Social Determinants of Health, (2) An Improved Provider Experience for Patients, (3) Patient as a Person with Complex Needs, and (4) The Learning Experience. - Findings suggest virtual reality has strong merits for impacting affective domain of learning demonstrated by increased empathy. Virtual reality along with increased empathy also helps improve attitudes and behaviours for the betterment of patients. | United States |
| **Grekin et al., 2019** | Grekin, E. R., Beatty, J. R., and Ondersma, S. J. (2019). Mobile health  interventions: exploring the use of common relationship factors. JMIR mHealth  7:e11245. doi: 10.2196/11245 | IV- Viewpoint | Mobile health interventions: exploring the use of common relationship factors | - The use of mobile health (mHealth) interventions has risen dramatically over the past two decades. It is important to consider mHealth intervention research within the broader therapy outcome literature. Among other key findings, this broader literature suggests that common relationship factors such as empathy, positive regard, and genuineness may play a critical role in therapy effectiveness. These findings raise intriguing questions for mobile interventions. For example, can mobile interventions incorporate aspects of common factors to augment their efficacy? Will the absence of relationship-based common factors make mobile interventions less effective? This viewpoint paper addresses these questions as well as related issues such as how to operationalize relationship qualities in the context of a mobile intervention and whether common relationship factors apply to computers or computerized narrators. The paper concludes by outlining a future research agenda guided by theory and empirical studies. | United States |
| **Groza et al., 2017** | Groza, H. L., Sebesi, S. B., and Mandru, D. S. (2017). “Age simulation suits for  training, research and development,” in Proceeding of the international conference  on advancements of medicine and health care through technology; 12th - 15th  october 2016, cluj-napoca, romania, (Cham: Springer), doi: 10.1007/978-3-319-  52875-5_17 | VI- Design study | Examples of simulation technologies for old age | - Simulation of old age can be a strong tool in providing information to researchers and developers for products that address to elderly people, and also for the disabled. Suggests that medical personal who are working with people that need care could be also users for an age simulator, with the purpose of understanding the needs of the ones they take care for. The benefits would be to provide better services and empathy. In this paper technical solutions are proposed for development of age simulation suits, in order to identify different ways of integrating the physical functions. | Romania |
| **Guetterman et al, 2019** | Guetterman, T. C., Sakakibara, R., Baireddy, S., Kron, F. W., Scerbo, M. W.,  Cleary, J. F., et al. (2019). Medical students’ experiences and outcomes using a  virtual human simulation to improve communication skills: mixed methods study.  J. Med. Int. Res. 21:e15459. doi: 10.2196/15459 | II- Mixed methods study | Medical students' experiences and outcomes using a virtual human simulation to improve communication Skills | - Outcomes were significantly improved for learners in the intervention group and qualitative analysis revealed 3 major positive themes for the MPathic-VR group learners: gaining useful communication skills, learning awareness of nonverbal skills in addition to verbal skills, and feeling motivated to learn more about communication. Finally, the results of the mixed methods analysis indicated that most of the variation between high, middle, and lower performers was noted about nonverbal behaviors. Medium and high OSCE scorers most often commented on the importance of nonverbal communication. Themes of motivation to learn about communication were only present in middle and high scorers. | United States |
| **Guetterman et al., 2017** | Guetterman, T. C., Kron, F. W., Campbell, T. C., Scerbo, M. W., Zelenski, A. B.,  Cleary, J. F., et al. (2017). Initial construct validity evidence of a virtual human  application for competency assessment in breaking bad news to a cancer patient.  Adv. Med. Educ. Pract. 25, 505–512. doi: 10.2147/AMEP.S138380 | VI- Design study | Study to evaluate the validity of a virtual human application, MPathic-VR, for assessing performance-based competence in breaking bad news (BBN) to a VH patient. | - Despite interest in using virtual humans (VHs) for assessing health care communication, evidence of validity is limited. - Results indicate that the VH program was sensitive to differences in assessing performance-based competence in breaking bad news (BBN) to a cancer patient. Improved pre-post scores demonstrate acquisition of skills in BBN to a VH patient. Pre-test sensitization did not appear to influence post-test assessment. These results provide initial construct validity evidence that the VH program is effective for assessing BBN performance-based communication competence. | United States |
| **Halan et al., 2015** | Halan, S., Sia, I., Crary, M., and Lok, B. (2015). “Exploring the effects of  healthcare students creating virtual patients for empathy training,” in Intelligent  virtual agents. IVA 2015. lecture notes in computer science, eds W. Brinkman, J.  Broekens, and D. Heylen (Cham: Springer), 9238. doi: 10.1007/978-3-319-21996-  7_24 | VI- Design study | Exploring the effects of healthcare students creating virtual patients for empathy training | - Intelligent virtual agents have been successfully used for interpersonal skills training of healthcare students by enabling simulated interactions between healthcare students and virtual patient agents. However, during these interactions, students do not get the opportunity to take the perspective of the patient. Taking the perspective of the patient is essential for healthcare students to learn critical interpersonal skills like empathy. - Study of healthcare students create virtual patient agents of a particular race to provide them the opportunity to take the perspective of patients from that race, leading to increased empathy during subsequent interactions with patients of that race. We conducted a semester-long user study with 24 healthcare students to explore the effects of having them create virtual patient agents. - Results indicate that healthcare students who created and interviewed virtual patients of the same race were significantly more empathetic than students who created virtual patients with a race discordant to the one they interacted with. | United States |
| **Harris, 2021** | Harris, J. (2021). Editorial commentary: personalized hip arthroscopy outcome  prediction using machine learning-the future is here. Arthroscopy 37, 1498–1502.  doi: 10.1016/j.arthro.2021.02.032 | IV- Editorial commentary | Personalized hip arthroscopy outcome prediction using machine learning | - Argues that efficient integration of machine learning into hip arthroscopy practice can reduce physicians' "busywork" of data collection and analysis. This can only improve the value of the patient experience, because surgeons have more time for shared decision making, with empathy, compassion, and humanity counterintuitively returning to medicine. | United States |
| **Hayakawa et al, 2022** | Hayakawa, J., Barrows, J., See, S., and Schomberg, J. (2022). Effects of classical  music virtual reality on pediatric healthcare worker compassion fatigue. J. Nurs.  Administr. 52, 280–285. doi: 10.1097/NNA.0000000000001148 | VI- Design study | Study to explore the impact of classical music virtual reality (VR) on burnout, secondary traumatic stress, anxiety, and capacity for developing caring relationships with patients among healthcare workers (HCWs) | - Explains that COVID-19 accentuated the importance of promoting the well-being of frontline workers. Efforts to address the mental health needs of HCWs are likely to positively impact patient outcomes. - Healthcare workers (n=71) completed 3 sessions of VR. A Wilcoxon rank-sum test was used to compare premeasure versus postmeasure on the Professional Quality of Life (ProQOL), State Trait Anxiety Inventory, and Caring Ability Inventory. Analysis of variance was performed to identify associations between the intervention and differences in scores for each ProQOL domain. - There was a significant reduction in burnout after the experience, compared with baseline. | United States |
| **He et al., 2022** | He, L., Basar, E., Wiers, R. W., Antheunis, M. L., and Krahmer, E. (2022). Can  chatbots help to motivate smoking cessation? A study on the effectiveness of  motivational interviewing on engagement and therapeutic alliance. BMC Public  Health 22:726. doi: 10.1186/s12889-022-13115-x | VI- Design study | Explores the possibility of using a motivational interviewing style chatbot to enhance engagement, therapeutic alliance, and perceived empathy in the context of smoking cessation | - Web-based experiment in which smokers (n = 153) were randomly assigned to either the motivational interviewing (MI)-style chatbot condition (n = 78) or the neutral chatbot condition (n = 75) and interacted with the chatbot in two sessions. Typical intake questions in smoking cessation interventions were administered by the chatbot, such as smoking history, nicotine dependence level, and intention to quit. In the feedback session, the chatbot provided personalized normative feedback and discussed with participants potential reasons to quit. Engagement with the chatbot, therapeutic alliance, and perceived empathy were the primary outcomes and were assessed after both sessions. Secondary outcomes were motivation to quit and perceived communication competence and were assessed after the two sessions. - No significant effects of the experimental manipulation (MI-style or neutral chatbot) were found on engagement, therapeutic alliance, or perceived empathy. A significant increase in therapeutic alliance over two sessions emerged in both conditions, with participants reporting significantly increased motivation to quit. The chatbot was perceived as highly competent, and communication competence was positively associated with engagement, therapeutic alliance, and perceived empathy. - The results of this preregistered study suggest that talking with a chatbot about smoking cessation can help to motivate smokers to quit and that the effect of conversation has the potential to build up over time. No extra motivating effect of the MI-style chatbot was found. These findings highlight the promise of using chatbots to motivate smoking cessation. | Netherlands |
| **Hernandez, 2019** | Hernandez, J. (2019). Network diffusion and technology acceptance of a nurse  chatbot for chronic disease self-management support: a theoretical perspective.  J. Med. Invest. JMI 66, 24–30. doi: 10.2152/jmi.66.24 | IV- Perspective | Network diffusion and technology acceptance of a nurse chatbot for chronic disease self-management support | - Explores the 'Nurse Chatbot' for chronic care on the benefits of increasing patient/client access to healthcare information and maximizing the potential of artificial intelligence/AI to bridge the 'demand-supply gap' of human healthcare providers. Argues that closing this gap through the establishment of a 'Nurse Chatbot' will be innovative, favourably scalable and customizable within a decentralized health network, and potentially sustainable in the new digital economy. - Following are the assumptions : 1) "caring" communicated textually is highly 'transactive' in chronic disease self-management support/CDSMS for goal agreements between agents and for overcoming the system noise in the form of cross-entropy, perplexity, and information wastage ; 2) 'Nurse Chatbot' is the interlocutor in nursing care and the nursing agency by superpositioning and entanglement ; and 3) possible effects of chatbot-user transactions are information flows, management of health, and patient satisfaction. - This article also looks into 'Nurse Chatbot' development for CDSMS, simulation of its diffusion capacity egocentrically, technology acceptance model/TAM to inquire the engagement of users, and possible approaches to ethical and safety issues. | Philippines |
| **Herrmann-Werner et al., 2021** | Herrmann-Werner, A., Loda, T., Zipfel, S., Holderried, M., Holderried, F., and  Erschens, R. (2021). Evaluation of a language translation app in an undergraduate  medical communication course: proof-of-concept and usability study. JMIR  mHealth 9:e31559. doi: 10.2196/31559 | II- Research study | A proof-of-concept pilot study was designed to evaluate the use of a speech-to-speech LTA in a specific simulated physician-patient situation, particularly its perceived usability, helpfulness, and meaningfulness, and to assess the teaching unit overall. | - Explains that language barriers in medical encounters pose risks for interactions with patients, their care, and their outcomes. Because human translators, the gold standard for mitigating language barriers, can be cost- and time-intensive, mechanical alternatives such as language translation apps (LTA) have gained in popularity. However, adequate training for physicians in using LTAs remains elusive. - Students engaged in a 90-min simulation with a standardized patient (SP) and the LTA iTranslate Converse. Thereafter, they rated the LTA with six items-helpful, intuitive, informative, accurate, recommendable, and applicable-on a 7-point Likert scale ranging from 1 (don't agree at all) to 7 (completely agree) and could provide free-text responses for four items: general impression of the LTA, the LTA's benefits, the LTA's risks, and suggestions for improvement. Students also assessed the teaching unit on a 6-point scale from 1 (excellent) to 6 (insufficient). Data were evaluated quantitatively with mean (SD) values and qualitatively in thematic content analysis. - Of 111 students in the course, 76 (68.5%) participated (59.2% women, age 20.7 years, SD 3.3 years). Values for the LTA's being helpful (mean 3.45, SD 1.79), recommendable (mean 3.33, SD 1.65) and applicable (mean 3.57, SD 1.85) were centered around the average of 3.5. The items intuitive (mean 4.57, SD 1.74) and informative (mean 4.53, SD 1.95) were above average. The only below-average item concerned its accuracy (mean 2.38, SD 1.36). Students rated the teaching unit as being excellent (mean 1.2, SD 0.54) but wanted practical training with an SP plus a simulated human translator first. Free-text responses revealed several concerns about translation errors that could jeopardize diagnostic decisions. Students feared that patient-physician communication mediated by the LTA could decrease empathy and raised concerns regarding data protection and technical reliability. Nevertheless, they appreciated the LTA's cost-effectiveness and usefulness as the best option when the gold standard is unavailable. They also reported wanting more medical-specific vocabulary and images to convey all information necessary for medical communication. - This study revealed the feasibility of using a speech-to-speech LTA in an undergraduate medical course. Although human translators remain the gold standard, LTAs could be valuable alternatives. Students appreciated the simulated teaching and recognized the LTA's potential benefits and risks for use in real-world clinical settings. To optimize patients' and health care professionals' experiences with LTAs, future investigations should examine specific design options for training interventions and consider the legal aspects of human-machine interaction in health care settings. | Germany |
| **Hershberger et al., 2022** | Hershberger, P. J., Pei, Y., Crawford, T. N., Neeley, S. M., Wischgoll, T., Patel,  D. B., et al. (2022). An interactive game with virtual reality immersion to improve  cultural sensitivity in health care. Health Equity. 6, 189–197. doi: 10.1089/heq.  2021.0128 | VI- Design of a virtual reality immersion training simulation | This training simulation aimed to utilize components of evidence-based prejudice habit breaking interventions, such as learning more about an individual's life experience to help minimize filling in gaps with stereotyped assumptions. | - Post-simulation data indicated increased feelings of compassion toward the patient and decreased expectations about how difficult future encounters with the patient would be. With respect to attribution, after the simulation participants were less inclined to view the patient as primarily responsible for their situation, suggesting less impact of the fundamental attribution error. - The findings suggest that although training simulations cannot fully replicate or replace the advantages that come with real-world experience, they can heighten awareness in the increase of increasing the cultural sensitivity of clinicians in health care professions for improving health equity. | United States |
| **Hess et al., 2022** | Hess, S. P., Levin, M., Akram, F., Woo, K., Andersen, L., Trenkle, K., et al. (2022).  The impact and feasibility of a brief, virtual, educational intervention for home  healthcare professionals on Parkinson’s disease and related disorders: pilot study  of I SEE PD home. BMC Med. Educ. 22:506. doi: 10.1186/s12909-022-03430-7 | VI- Design study | The impact and feasibility of a brief, virtual, educational intervention for home healthcare professionals on Parkinson's Disease and related disorders (pilot study of I SEE PD Home) | - Home health nurses, occupational therapists, physical therapists and physical therapy assistants, and speech-language pathologists participated in a daylong, virtual symposium on advanced PD/PRD, combining focused lectures, discipline-specific breakout sessions, immersive virtual reality vignettes, and interactive panels with both patients and families, and movement disorders and home healthcare experts. - Common themes regarding symposium-motivated practice change included: interdisciplinary collaboration; greater involvement and weighting of the patient and caregiver voice in care plans; attention to visit scheduling in relation to patient function; recognition and practical management of the causes of sudden change in PD/PRD, including infections and orthostatic hypotension. - A virtual, multimodal, brief educational pilot intervention improved PD/PRD-specific knowledge and confidence among home healthcare nurses and allied health professionals. Future studies are necessary to test the short- and long-term effects of this intervention more broadly and to investigate the impact of this education on patient and caregiver outcomes. | United States |
| **Hirt and Beer, 2020** | Hirt, J., and Beer, T. (2020). Use and impact of virtual reality simulation in  dementia care education: a scoping review. Nurse Educ. Today. 84:104207. doi:  10.1016/j.nedt.2019.104207 | III- Scoping review | Use and impact of virtual reality simulation in dementia care education | - The review process resulted in the inclusion of six studies published between 2012 and 2017. Two of them are ongoing studies. Three studies had a one group pre-post-test design and in one study a post-test only design was applied. The samples consisted of caregivers of people with dementia as well as students and varied in size between seven and 126. Eight different outcomes were measured, e.g. empathy, competence, and stress. Interventions resulted in improvements of caregivers' and students' empathy and competences among other outcomes. - No studies with controlled design and group comparisons are available yet. There are some indications that virtual reality might be an effective intervention to train caregivers of persons with dementia. Little is known about the use and impact of virtual reality in dementia-related education. Since studies are rare and do not address effectiveness, the findings of this review can substantially contribute to guide further research on this topic. | Switzerland |
| **Hopkins et al., 2021** | Hopkins, C. M., Miller, H. N., Brooks, T. L., Mo-Hunter, L., Steinberg,  D. M., and Bennett, G. G. (2021). Designing ruby: protocol for a 2-arm, brief,  digital randomized controlled trial for internalized weight bias. JMIR Res. Protoc.  10:e31307. doi: 10.2196/31307 | II- Research study (protocol) | Digital randomized controlled trial for internalized weight bias | - Weight bias internalization, also known as weight self-stigma, is a serious health concern for individuals with higher body weight. Elevated weight bias internalization has been associated with low self-compassion, yet few investigations have explored self-compassion as a potential mechanism for reducing internalized weight bias. - Ruby is a 2-arm randomized controlled trial that was designed to test the efficacy of a 4-week digital self-compassion intervention to reduce internalized weight bias compared with a wait-list control. The intervention content will include psychoeducation and daily mindfulness practices with a focus on self-compassion and body concerns. Ruby will be the first digital standalone, self-compassion-based intervention designed to reduce internalized weight bias. - Owing to its standalone digital delivery, Ruby may be a highly scalable treatment for internalized weight bias that can be delivered on its own or combined with other treatments. It is designed to be accessible to many, as participants can access the digital intervention at times of the day that are the most convenient in their schedule and are not burdened by in-person time commitments, which can be a barrier for participants with competing demands on their time and resources. | United States |
| **Hou et al., 2020** | Hou, I. C., Lan, M. F., Shen, S. H., Tsai, P. Y., Chang, K. J., Tai, H. C., et al. (2020).  The development of a mobile health app for breast cancer self-management  support in taiwan: design thinking approach. JMIR mHealth uHealth 8:e15780.  doi: 10.2196/15780 | VI- Design study | Study to investigate the information needs of Taiwanese women with breast cancer to inform the development of a self-management support mHealth app | - Highlights that evidence has shown that breast cancer self-management support from mobile health (mHealth) apps can improve the quality of life of survivors. Although many breast cancer self-management support apps exist, few papers have documented the procedure for the development of a user-friendly app from the patient's perspective. - A 5-step design thinking approach, comprising empathy, define, ideate, prototype, and test steps, was used in the focus groups and individual interviews conducted to collect information on the requirements and expectations of Taiwanese women with breast cancer with respect to the app. A thematic analysis was used to identify information needs. - A total of 8 major themes including treatment, physical activity, diet, emotional support, health records, social resources, experience sharing, and expert consultation were identified. Minor themes included the desire to use the app under professional supervision and a trustworthy app manager to ensure the credibility of information. - The strengths of the design thinking approach were user-centered design and cultural sensitivity. The results retrieved from each step contributed to the development of the app and reduction of the gap between end users and developers. An mHealth app that addresses these 8 main themes can facilitate disease self-management for Taiwanese women with breast cancer. | Taiwan |
| **Inkster et al., 2018** | Inkster, B., Sarda, S., and Subramanian, V. (2018). An empathy-driven,  conversational artificial intelligence agent (wysa) for digital mental wellbeing:  real-world data evaluation mixed-methods study. JMIR mHealth uHealth  6:e12106. doi: 10.2196/12106 | VI- Design study | Presents a preliminary real-world data evaluation of the effectiveness and engagement levels of an AI-enabled, empathetic, text-based conversational mobile mental well-being app (Wysa) on users with self-reported symptoms of depression | - Explains that a World Health Organisation 2017 report stated that major depression affects almost 5% of the human population. Major depression is associated with impaired psychosocial functioning and reduced quality of life. Challenges such as shortage of mental health personnel, long waiting times, perceived stigma, and lower government spends pose barriers to the alleviation of mental health problems. Face-to-face psychotherapy alone provides only point-in-time support and cannot scale quickly enough to address this growing global public health challenge. Artificial intelligence (AI)-enabled, empathetic, and evidence-driven conversational mobile app technologies could play an active role in filling this gap by increasing adoption and enabling reach. Although such a technology can help manage these barriers, they should never replace time with a health care professional for more severe mental health problems. However, app technologies could act as a supplementary or intermediate support system. Mobile mental well-being apps need to uphold privacy and foster both short- and long-term positive outcomes. - The average mood improvement (ie, difference in pre- and post-self-reported depression scores) between the groups (ie, high vs low users; n=108 and n=21, respectively) revealed that the high users group had significantly higher average improvement (mean 5.84 [SD 6.66]) compared with the low users group (mean 3.52 [SD 6.15]); Mann-Whitney P=.03 and with a moderate effect size of 0.63. Moreover, 67.7% of user-provided feedback responses found the app experience helpful and encouraging. - The real-world data evaluation findings on the effectiveness and engagement levels of Wysa app on users with self-reported symptoms of depression show promise. However, further work is required to validate these initial findings in much larger samples and across longer periods. | United Kingdom |
| **Irfan, 2021** | Irfan, F. (2021). Artificial intelligence: help or hindrance for family physicians?  Pak. J. Med. Sci. 37, 288–291. doi: 10.12669/pjms.37.1.3351 | IV- Commentary | Artificial intelligence and family physicians | - Argues that artificial Intelligence can be a solution in the future as a physician's new assistant; AI-physician combinations can act like models of 'peaceful co-existence'. While it has the potential to mould many dimensions of patient care and can augment quality improvement, it cannot replace a family physician's diagnostic intelligence, empathy and relationships. Physicians need to strike a balance between these combinations for better health outcomes without increasing patients' frustration. | Pakistan |
| **Jacobs and Maidwell-Smith, 2022** | Jacobs, C., and Maidwell-Smith, A. (2022). Learning from 360-degree film in  healthcare simulation: a mixed methods pilot. J. Visual Commun. Med. 2022:7059.  doi: 10.1080/17453054.2022.2097059 | II- Research study | Study to compare learning from a clinical encounter viewed in a virtual reality 360-degree headset to that of a traditional monitor by quantifying the user experience and testing what was learnt | - Technology that delivers an immersive experience in education offers a viable alternative to in-person teaching. Furthermore, experiential learning is described as a key concept in simulation practice, and this is explored using transcripts of participants' experiences with 360-degree video. - No statistical difference between median exam scores between groups (p = 0.25), and there was no correlation found between total immersion and motivational scores with exam performance (Rho = -0.14 p = 0.18, Rho = 0.08 p = 0.31). However, those viewing 360 media reported significantly higher immersion, motivation, and empathy scores (p < 0.05). Domains based upon Kolb's learning cycle generated themes including engagement, communication, and self-efficacy. - Concludes that 360 video creates an immersive experience with an associated high-value motivational position; however, this could not be translated to an increase in exam scores. There are benefits to perceived learning and emotional content with 360 videos, although, pedagogical theory needs further understanding if educators are to embed new immersive technology in curriculums. | United Kingdom |
| **James et al., 2021** | James, J., Balamurali, B. T., Watson, C. I., et al. (2021). Empathetic speech  synthesis and testing for healthcare robots. Int. J. Soc. Rob. 13, 2119–2137. doi:  10.1007/s12369-020-00691-4 | VI- Design study | Study to find out if social robots with empathetic voice are acceptable for users in healthcare applications | - One of the major factors that affect the acceptance of robots in human-robot Interaction applications is the type of voice with which they interact with humans. The robot's voice can be used to express empathy, which is an affective response of the robot to the human user. - A pilot study using an empathetic voice spoken by a voice actor was conducted. Only prosody in speech is used to express empathy here, without any visual cues. Also, the emotions needed for an empathetic voice are identified. - It was found that the emotions needed are not only the stronger primary emotions, but also the nuanced secondary emotions. These emotions are then synthesised using prosody modelling. A second study, replicating the pilot test is conducted using the synthesised voices to investigate if empathy is perceived from the synthetic voice as well. - This paper reports the modelling and synthesises of an empathetic voice, and experimentally shows that people prefer empathetic voice for healthcare robots. | New Zealand |
| **Jie et al, 2020** | Jie, L. J., Jamin, G., Smit, K., Beurskens, A., and Braun, S. (2020). Design of the  user interface for “stappy”, a sensor-feedback system to facilitate walking in people  after stroke: a user-centred approach. Disabil Rehabil Assist Technol. 15, 959–967.  doi: 10.1080/17483107.2019.1629654 | VI- Design study | Design of a user interface for ‘Stappy’ a sensor feedback system to facilitate walking in people after stroke | - Sensor-feedback systems can be used to support people after stroke during independent practice of gait. The main aim of the study was to describe the user-centred approach to (re)design the user interface of the sensor feedback system "Stappy" for people after stroke and share the deliverables and key observations from this process. - The user-centred approach was structured around four phases (the discovery, definition, development and delivery phase) which were fundamental to the design process. Fifteen participants with cognitive and/or physical limitations participated (10 women, 2/3 older than 65). Prototypes were evaluated in multiple test rounds, consisting of 2-7 individual test sessions. - Seven deliverables were created: a list of design requirements, a personae, a user flow, a low-, medium- and high-fidelity prototype and the character "Stappy". The first six deliverables were necessary tools to design the user interface, whereas the character was a solution resulting from this design process. Key observations related to "readability and contrast of visual information", "understanding and remembering information", "physical limitations" were confirmed by and "empathy" was additionally derived from the design process. - The study offers a structured methodology resulting in deliverables and key observations, which can be used to (re)design meaningful user interfaces for people after stroke. Additionally, the study provides a technique that may promote "empathy" through the creation of the character Stappy. The description may provide guidance for health care professionals, researchers or designers in future user interface design projects in which existing products are redesigned for people after stroke. | Netherlands |
| **Jiménez-Rodríguez et al., 2021** | Jiménez-Rodríguez, D., Pérez-Heredia, M., Molero Jurado, M. D. M., Pérez-  Fuentes, M. D. C., and Arrogante, O. (2021). Improving humanization skills  through simulation-based computers using simulated nursing video consultations.  Healthcare 10:37. doi: 10.3390/healthcare10010037 | VI- Design study | Improving humanization skills through simulation-based computers using simulated nursing video consultations | - Evaluates the effects of virtual simulation-based training on developing and cultivating humanization competencies in undergraduate nursing students. A quasi-experimental study was conducted with 60 undergraduate nursing students. A validated questionnaire was used to evaluate the acquisition of humanization competencies (self-efficacy, sociability, affection, emotional understanding, and optimism). The development of humanization competencies in this group composed of undergraduate nursing students was evaluated using virtual simulation-based training, comparing the levels obtained in these competencies at baseline (pre-test) and after the virtual simulation experience (post-test). - After the virtual simulation sessions, students improved their levels in humanization total score and the emotional understanding and self-efficacy competencies, obtaining large effects sizes in all of them (rB = 0.508, rB = 0.713, and rB = 0.505 respectively). This virtual simulation modality enables training in the humanization of care with the collaboration of standardized patients in the form of simulated nursing video consultations and the performance of high-fidelity simulation sessions that comply with the requirements of best practices. Therefore, this methodology could be considered as another choice for virtual simulation. Additionally, this virtual modality could be a way to humanize virtual simulation. | Spain |
| **Joda et al., 2020** | Joda, T., Bornstein, M. M., Jung, R. E., Ferrari, M., Waltimo, T., and Zitzmann,  N. U. (2020). Recent trends and future direction of dental research in the digital  era. Int. J. Environ. Res. Public Health. 17:1987. doi: 10.3390/ijerph17061987 | IV- Opinion piece | Digital transformation of dental medicine | - Identifies estimated top five trends and innovations of this new digital era, with potential to decisively influence the direction of dental research: (1) rapid prototyping (RP), (2) augmented and virtual reality (AR/VR), (3) artificial intelligence (AI) and machine learning (ML), (4) personalized (dental) medicine, and (5) tele-healthcare. - Suggests that digital dentistry requires managing expectations pragmatically and ensuring transparency for all stakeholders: patients, healthcare providers, university and research institutions, the medtech industry, insurance, public media, and state policy. It should not be claimed or implied that digital smart data technologies will replace humans providing dental expertise and the capacity for patient empathy. The dental team that controls digital applications remains the key and will continue to play the central role in treating patients. In this context, the latest trend word is created: augmented intelligence, e.g., the meaningful combination of digital applications paired with human qualities and abilities in order to achieve improved dental and oral healthcare, ensuring quality of life. | Switzerland |
| **Johanson et al., 2019** | Johanson, D. L., Ahn, H. S., MacDonald, B. A., Ahn, B. K., Lim, J., Hwang,  E., et al. (2019). The effect of robot attentional behaviors on user perceptions  and behaviors in a simulated health care interaction: randomized controlled trial.  J. Med. Int. Res. 21:e13667. doi: 10.2196/13667 | II- Randomized controlled trial in educational setting | Study to examine the effect of robot attentional behaviours on user perceptions and behaviours in a simulated health care interaction | - A total of 181 participants were recruited from the University of Auckland. Participants who interacted with the robot in the forward lean and self-disclosure conditions found the robot to be significantly more stimulating than those who interacted with the robot in the voice pitch or neutral conditions (P=.03). Participants in the forward lean, self-disclosure, and neutral conditions found the robot to be significantly more interesting than those in the voice pitch condition (P<.001). Participants in the forward lean and self-disclosure conditions spent significantly more time looking at the robot than participants in the neutral condition (P<.001). Significantly, more participants in the self-disclosure condition laughed during the interaction (P=.01), whereas significantly more participants in the forward lean condition leant toward the robot during the interaction (P<.001). - The use of self-disclosure and forward lean by a health care robot can increase human engagement and attentional behaviours. Voice pitch changes did not increase attention or engagement. The small effects with regard to participant perceptions are potentially because of the limitations in self-report measures or a lack of comparison for most participants who had never interacted with a robot before. Further research could explore the use of self-disclosure and forward lean using a within-subjects design and in real health care settings. | New Zealand |
| **Johanson et al., 2021** | Johanson, D. L., Ahn, H. S., and Broadbent, E. (2021). Improving interactions  with healthcare robots: a review of communication behaviours in social and  healthcare contexts. Int. J. Soc. Robot. 13, 1835–1850. doi: 10.1007/s12369-020-  00719-9 | III- Review | This literature review aimed to inform healthcare robotics research by highlighting communication behaviours that are important within the context of healthcare. The review focussed on relevant research in human clinical interactions, followed by a review of similar factors in social robotics research. | - A growing shortfall exists between the number of older individuals who require healthcare support and the number of qualified healthcare professionals who can provide this. Robots offer the potential to provide healthcare support to patients both at home and in healthcare settings. However, in order for robots to be successfully implemented in these environments, they need to behave in ways that are appropriate and acceptable to human users. - Suggests one way to identify appropriate social behaviours for healthcare robots is to model their behaviour on interactions between healthcare professionals and patients. Three databases were searched for terms relating to healthcare professional communication behaviours associated with patient outcomes. - Results identified key communication behaviours that can convey clinical empathy, including humour, self-disclosure, facial expressions, eye gaze, body posture, and gestures. A further search was conducted to identify research examining these key behaviours within the context of social and healthcare robotics. Research into these factors in human-robot interaction in healthcare is limited to date, and these findings can inform future research. | New Zealand |
| **Johnston, 2018** | Johnston, S. C. (2018). Anticipating and training the physician of the future: the  importance of caring in an age of artificial intelligence. Acad. Med. J. Assoc. Am.  Med. Coll. 93, 1105–1106. doi: 10.1097/ACM.0000000000002175 | IV- Perspective | Explores AI and future of medicine and medical practice | - Suggests that artificial intelligence and other forms of information technology are only just beginning to change the practice of medicine. The pace of change is expected to accelerate as tools improve and as demands for analysing a rapidly growing body of knowledge and array of data increase. - Suggests that the medical students of today will practice in a world where information technology is sophisticated and omnipresent. In this world, the tasks of memorization and analysis will be less important to them as practicing physicians. On the other hand, the nonanalytical, humanistic aspects of medicine-most importantly, the art of caring-will remain a critical function of the physician, and facility with improving systems of care will be required. Communication, empathy, shared decision making, leadership, team building, and creativity are all skills that will continue to gain importance for physicians. Argues that these skills should be further prioritized in medical school curricula to produce an even more effective physician for the future. | United States |
| **Jones et al., 2021** | Jones, C., Jones, D., and Moro, C. (2021). Use of virtual and augmented realitybased  interventions in health education to improve dementia knowledge and  attitudes: an integrative review. BMJ Open. 11:e053616. doi: 10.1136/bmjopen-  2021-053616 | III- Integrative review | Use of virtual and augmented reality-based interventions in health education to improve dementia knowledge and attitudes | - Immersive technologies such as virtual (VR) and augmented reality (AR) can potentially help health professionals and trainees understand psychological symptoms and responsive behaviours associated with dementia within a safe and supportive learning environment. - Immersive virtual learning potentially enhances knowledge, attitudes, empathy and sensitivity of health professionals and trainees. While promising, there remains a lack of conclusive and robust evidence to fully recommend the introduction and inclusion of immersive virtual learning in dementia education and training. Additional rigorously designed research studies with larger sample sizes are needed to confirm the benefits on attitudes, empathy, sensitivity and knowledge. | Australia |
| **Jones-Schenk, 2016** | Jones-Schenk, J. (2016). Getting to the root of disparities: social cognition and  the affective domain. J. Contin. Educ. Nurs. 47, 443–445. doi: 10.3928/00220124-  20160920-04 | IV- Perspective | Virtual reality technology and research on implicit bias to address healthcare quality | - Suggests that bias, prejudice, cultural insensitivity, and eroding levels of empathy all affect the health and well-being of patients and families and manifest or accelerate social disparities of health. For caregivers, educational offerings and activities targeting the affective domain can positively influence the development of greater empathy and improved social cognition. As difficult as it is to develop effective teaching methods for this domain, new strides in virtual reality technology and new research on implicit bias can provide the professional development educator with options in designing educational offerings that can help. | United States |
| **Jütten et al., 2018** | Jütten, L. H., Mark, R. E., and Sitskoorn, M. M. (2018). Can the mixed virtual  reality simulator into d’mentia enhance empathy and understanding and decrease  burden in informal dementia caregivers? Dement Geriatr Cogn. Dis. Extra. 8,  453–466. doi: 10.1159/000494660 | VI- Design study | Study of whether mixed virtual reality simulator D'mentia enhances empathy and understanding and decrease burden in informal dementia caregivers | - Caregivers indicated that the Into D'mentia intervention improved their understanding of dementia, that they had learned to be more patient, to take things more slowly, and to focus on positive aspects of caregiving. However, no significant change was found on the variables assessed via the questionnaires. Future research can consider enriching this intervention with other aspects such as more educational material, more simulations, and group sessions, tailored to the individual caregiver and his/her situation, and examine whether these new interventions yield change on questionnaires. - More personalized interventions for dementia caregivers could help caregivers to better understand the persons with dementia they care for and to ultimately enhance the well-being of both caregivers and persons with dementia. | Netherlands |
| **Kemp et al., 2020** | Kemp, J., Zhang, T., Inglis, F., Wiljer, D., Sockalingam, S., Crawford, A., et al.  (2020). Delivery of compassionate mental health care in a digital technology driven age: scoping review. J. Med. Int. Res. 22:e16263. doi: 10.2196/16263 | III- Literature review | How digital technologies are being used in the delivery of compassionate mental health care, and the facilitators of and barriers to digital technology use among patients and health professionals | - Telemedicine was the most widely used technology by mental health professionals. Digital technologies were described as facilitating compassionate care and were classified using a conceptual model to identify each digital intersection with compassionate care. - Facilitators of and barriers to providing compassionate care through digital technology were increased safety for providers, health care professional perceptions and abilities, and the use of picture-in-picture feedback to evaluate social cues. - Mental health professionals and organisations alike should be mindful that compassionate human-centered care is maintained in the delivery of digital health care. | Canada |
| **Kennedy et al., 2012** | Kennedy, C. M., Powell, J., Payne, T. H., Ainsworth, J., Boyd, A., and Buchan,  I. (2012). Active assistance technology for health-related behavior change: an  interdisciplinary review. J. Med. Int. Res. 14:e80. doi: 10.2196/jmir.1893 | III- Literature review | Literature review to determine the extent to which the active technological capabilities of dynamic and adaptive information processing are being applied in behaviour change interventions and to identify their role in these interventions. | - Information technology can help individuals to change their health behaviours. This is due to its potential for dynamic and unbiased information processing enabling users to monitor their own progress and be informed about risks and opportunities specific to evolving contexts and motivations. - Key categories of active technology such as semantic information processing, pattern recognition, and adaptation. Literature search using keywords derived from the categories and included studies that indicated a significant role for an active technology in health-related behaviour change. Data extraction looked specifically for the following technology roles: (1) dynamic adaptive tailoring of messages depending on context, (2) interactive education, (3) support for client self-monitoring of behaviour change progress, and (4) novel ways in which interventions are grounded in behaviour change theories using active technology. - Results show that significant research was focused on dialog systems, embodied conversational agents, and activity recognition. The most covered health topic was physical activity. The majority of the studies were early-stage research. Only 6 were randomized controlled trials, of which 4 were positive for behaviour change and 5 were positive for acceptability. Empathy and relational behaviour were significant research themes in dialog systems for behaviour change. - Argues that the potential capabilities and risks of active assistance technologies are not being fully explored in most current behaviour change research. Designers of health behaviour interventions need to consider the relevant informatics methods and algorithms more fully. | United Kingdom |
| **Kerasidou, 2020** | Kerasidou, A. (2020). Artificial intelligence and the ongoing need for empathy,  compassion and trust in healthcare. Bull World Health Organ. 98, 245–250. doi:  10.2471/BLT.19.237198 | IV- Commentary | Artificial intelligence in efficient, empathetic and trustworthy health care | - This article considers the vision of efficient, empathetic and trustworthy health care put forward by the proponents of artificial intelligence. The author suggest that artificial intelligence has the potential to fundamentally alter the way in which empathy, compassion and trust are currently regarded and practised in health care. Moving forward, it is important to re-evaluate whether and how these values could be incorporated and practised within a health-care system where artificial intelligence is increasingly used. Most importantly, society needs to re-examine what kind of health care it ought to promote. | United Kingdom |
| **Kerr and Klonoff, 2019** | Kerr, D., and Klonoff, D. C. (2019). Digital diabetes data and artificial  intelligence: a time for humility not hubris. J. Diab. Sci. Technol. 13, 123–127.  doi: 10.1177/1932296818796508 | IV- Commentary | Digital diabetes data and artificial intelligence | - Argues that in the future artificial intelligence (AI) will have the potential to improve outcomes diabetes care. With the creation of new sensors for physiological monitoring sensors and the introduction of smart insulin pens, novel data relationships based on personal phenotypic and genotypic information will lead to selections of tailored, effective therapies that will transform health care. - Highlights that decision-making processes based exclusively on quantitative metrics that ignore qualitative factors could create a quantitative fallacy. Difficult to quantify inputs into AI-based therapeutic decision-making processes include empathy, compassion, experience, and unconscious bias. Failure to consider these "softer" variables could lead to important errors. In other words, that which is not quantified about human health and behaviour is still part of the calculus for determining therapeutic interventions. | United States |
| **Kerruish, 2021** | Kerruish, E. (2021). Assembling human empathy towards care robots: the  human labor of robot sociality. Emot. Space Soc. 41:100840. doi: 10.1016/j.emospa.  2021.100840 | VI- Design study | Human empathy towards care robots | - Conceptualizes human to robot empathy as empathetic arrangements configured in caring spaces. Analyzing empathy towards care robots as arrangements comprising robots, spaces, discourses, bodies and institutions enables recognition of the way empathy is about self-other relationships while eschewing an understanding of empathy in terms of a reciprocal relationship between human and robot. - Situating the therapeutic, zoomorphic robot, Paro and the health care support robot, Care-O-Bot as part of empathetic arrangements draws attention to how the cultivation of empathy towards robots governs and regulates patient sociality. In particular, it shows that these robots do not function as substitutes for human carers but instead are dependent on human labour if they are to deliver therapy ethically and effectively. They rely on the affective labour of the patient and the labour of carers and others in the arrangement. | Australia |
| **Kim and Chun, 2022** | Kim, H., and Chun, J. (2022). Effects of a patient experience–based virtual reality  blended learning program on nursing students. CIN Comput. Inform. Nurs. 40,  438–446. doi: 10.1097/CIN.0000000000000817 | VI- Design study | Effects of a patient experience-based virtual reality blended learning program on nursing students | - Evaluated the performance of the patient experience virtual reality blended learning program developed for nursing students. The program enables nursing students to not only experience being perioperative patients themselves but also experience their conditions in places other than hospitals, which are generally used as training locations. - Results indicate that nursing students who virtually experienced the conditions of perioperative patients through virtual reality blended learning showed increased levels of empathy, positive attitudes toward patient safety treatment, confidence in nursing care, and clinical skill performance. The developed program in this study blended various teaching methods with a virtual reality platform to help nursing students with practical and effective perioperative training increase their levels of empathy by simulating the experiences and perspectives of perioperative patients. | Republic of Korea |
| **Kipnis et al., 2022** | Kipnis, E., McLeay, F., Grimes, A., de Saille, S., and Potter, S. (2022). Service  robots in long-term care: a consumer-centric view. J. Serv. Res. 2022:10849. doi:  10.1177/10946705221110849 | VI- Design study | Service robots in long-term care from a consumer perspective A Consumer-Centric View | - Service robots with advanced intelligence capabilities can potentially transform servicescapes. However, limited attention has been given to how consumers experiencing vulnerabilities, particularly those with disabilities, envisage the characteristics of robots' prospective integration into emotionally intense servicescapes, such as long-term care (LTC). - Exploratory studies with consumers with disabilities involving Community Philosophy, LEGO( (R) ) Serious Play( (R) ), and Design Thinking methods. Addressing a lack of consumer-centric research, we offer a three-fold contribution by 1) developing a conceptualization of consumer-conceived value of robots in LTC, which are envisaged as a supporting resource offering consumers opportunities to realize value; 2) empirically evidencing pathogenic vulnerabilities as a potential value-destruction factor to underscore the importance of integrating service robots research with a service inclusion paradigm; and 3) providing a theoretical extension and clarification of prior characterizations of robots' empathetic and emotion-related AI capabilities. - Consumers with disabilities conceive robots able to stimulate and regulate emotions by mimicking cognitive and behavioral empathy, but unable to express affective and moral empathy, which is central to care experience. Suggests that while providing support for care practices, for the foreseeable future, service robots will not, in themselves, actualize the experience of "being cared for." | United Kingdom |
| **Kocaballi et al., 2020a** | Kocaballi, B., Ijaz, K., Laranjo, L., Quiroz, J., Rezazadegan, D., Ly Tong, H., et al.  (2020a). Envisioning an artificial intelligence documentation assistant for future  primary care consultations: a co-design study with general practitioners. J. Am.  Med. Inform. Assoc. 27, 1695–1704. doi: 10.1093/jamia/ocaa131 | VI- Design study | Study to understand the potential roles of a future artificial intelligence (AI) documentation assistant in primary care consultations and to identify implications for doctors, patients, healthcare system, and technology design from the perspective of general practitioners | - Three co-design workshops with 16 general practitioners identified issues about: professional autonomy, human-AI collaboration, and new models of care. Major implications identified within these themes included (1) concerns with medico-legal aspects arising from constant recording and accessibility of full consultation records, (2) future consultations taking place out of the exam rooms in a distributed system involving empowered patients, (3) human conversation and empathy remaining the core tasks of doctors in any future AI-enabled consultations, and (4) questioning the current focus of AI initiatives on improved efficiency as opposed to patient care. - Suggests that AI documentation assistants will likely to be integral to the future primary care consultations. However, these technologies will still need to be supervised by a human until strong evidence for reliable autonomous performance is available. Therefore, different human-AI collaboration models will need to be designed and evaluated to ensure patient safety, quality of care, doctor safety, and doctor autonomy. | Australia |
| **Kocaballi et al., 2020b** | Kocaballi, B., Quiroz, J. C., Rezazadegan, D., Berkovsky, S., Magrabi, F., Coiera,  E., et al. (2020b). Responses of conversational agents to health and lifestyle prompts: investigation of appropriateness and presentation structures. J. Med. Int. Res. 22:e15823. doi: 10.2196/15823 | II- Research study | Study to analyse how commonly available, general-purpose conversational agents on smartphones and smart speakers respond to health and lifestyle prompts (questions and open-ended statements) | - Conversational agents (CAs) are systems that mimic human conversations using text or spoken language. Their widely used examples include voice-activated systems such as Apple Siri, Google Assistant, Amazon Alexa, and Microsoft Cortana. The use of CAs in health care has been on the rise, but concerns about their potential safety risks often remain understudied. - The 8 studied CAs provided in total 240 responses to 30 prompts. They collectively responded appropriately to 41% (46/112) of the safety-critical and 39% (37/96) of the lifestyle prompts. The ratio of appropriate responses deteriorated when safety-critical prompts were rephrased or when the agent used a voice-only interface. The appropriate responses included mostly directive content and empathy statements for the safety-critical prompts and a mix of informative and directive content for the lifestyle prompts. - These results suggest that the commonly available, general-purpose CAs on smartphones and smart speakers with unconstrained natural language interfaces are limited in their ability to advise on both the safety-critical health prompts and lifestyle prompts. The study also identified some response structures the CAs employed to present their appropriate responses. Further investigation is needed to establish guidelines for designing suitable response structures for different prompt types. | Australia |
| **Konstantinidis et al., 2022** | Konstantinidis, S., Leonardini, L., Stura, C., et al. (2022). “Digital soft skills  of healthcare workforce – identification, prioritization and digital training,” in  Mobility for smart cities and regional development - challenges for higher education.  ICL 2021, eds M. E. Auer, H. Hortsch, O. Michler, and T. Köhler (Cham: Springer),  doi: 10.1007/978-3-030-93907-6_117 | II- Interview study | Digital soft skills of the healthcare workforce | - Identifies and prioritizes digital soft skills for healthcare workforce based on 32 healthcare professionals, training providers and technology providers. From the analysis of the data collected by the interviews, it is clear that digital skills/competences can only be effectively put in practice in the health and care sectors if essential soft skills are enhanced: Communication, Open Mindedness, Positive Attitude, Critical Thinking and Empathy. This work also discusses potential training through role playing both as a face-to-face or as part of an immersive learning experience. | Germany |
| **Kovalchuk et al., 2022** | Kovalchuk, Y., Budini, E., Cook, R. M., and Walsh, A. (2022). Investigating the relationship between facial mimicry and empathy. Behav. Sci. 12:250. doi: 10.3390/bs12080250 | II- Research Study | Uses statistical and machine learning methods to explore the relationship between empathetic ability and facial mimicry (response to faces portraying different emotions) | - Facial expressions play a key role in interpersonal communication when it comes to negotiating our emotions and intentions, as well as interpreting those of others. Research has shown that we can connect to other people better when we exhibit signs of empathy and facial mimicry. However, the relationship between empathy and facial mimicry is still debated. Among the factors contributing to the difference in results across existing studies is the use of different instruments for measuring both empathy and facial mimicry, as well as often ignoring the differences across various demographic groups. - This study first looks at the differences in the empathetic abilities of people across different demographic groups based on gender, ethnicity and age. The empathetic ability is measured based on the Empathy Quotient, capturing a balanced representation of both emotional and cognitive empathy. Using statistical and machine learning methods, this study then investigates the correlation between the empathetic ability and facial mimicry of subjects in response to images portraying different emotions displayed on a computer screen. Unlike the existing studies measuring facial mimicry using electromyography, this study employs a technology detecting facial expressions based on video capture and deep learning. This choice was made in the context of increased online communication during and after the COVID-19 pandemic. - The results of this study confirm the previously reported difference in the empathetic ability between females and males. However, no significant difference in empathetic ability was found across different age and ethnic groups. Furthermore, no strong correlation was found between empathy and facial reactions to faces portraying different emotions shown on a computer screen. Overall, the results of this study can be used to inform the design of online communication technologies and tools for training empathy team leaders, educators, social and healthcare providers. | United Kingdom |
| **Krieger et al., 2021** | Krieger, J. L., Neil, J. M., Duke, K. A., Zalake, M. S., Tavassoli, F., Vilaro, M. J.,  et al. (2021). A pilot study examining the efficacy of delivering colorectal cancer  screening messages via virtual health assistants. Am. J. Prev. Med. 61, 251–255.  doi: 10.1016/j.amepre.2021.01.014 | VI- Design study | The purpose of this pilot study is to determine the efficacy of the Agent Leveraging Empathy for eXams virtual healthcare assistant intervention to increase patient intentions to talk to their doctor about colorectal cancer screening | - Patients are more likely to complete colorectal cancer screening when recommended by a race-concordant healthcare provider. Leveraging virtual healthcare assistants to deliver tailored screening interventions may promote adherence to colorectal cancer screening guidelines among diverse patient populations. - Examines the influence of animation and race concordance on intentions to complete colorectal cancer screening. Animated virtual healthcare assistants were efficacious compared with the static virtual healthcare assistant and attention control conditions. The influence of race concordance between source and participant was inconsistent across conditions. This warrants additional investigation in future studies given the potential for virtual healthcare assistant-assisted interventions to promote colorectal cancer screening within guidelines. | United States |
| **Law et al., 2019** | Law, M., Sutherland, C., Ahn, H. S., MacDonald, B. A., Peri, K., Johanson,  D. L., et al. (2019). Developing assistive robots for people with mild cognitive  impairment and mild dementia: a qualitative study with older adults and experts  in aged care. BMJ Open. 9:e031937. doi: 10.1136/bmjopen-2019-031937 | II- Qualitative research | Development of assistive robots for people with mild cognitive impairment and mild dementia | - This research is part of an international project to design and test a home-based healthcare robot to help older adults with mild cognitive impairment (MCI) or early dementia. The aim was to investigate the perceived usefulness of different daily-care activities for the robot, developed from previous research on needs. - Qualitative descriptive analysis using semi structured interviews. Two studies were conducted. In the first study, participants watched videos of a prototype robot performing daily-care activities; in the second study, participants interacted with the robot itself. - The themes that emerged included aspects of the robot's interactions, potential benefits, the appearance, actions and humanness of the robot, ways to improve its functionality and technical issues. Overall, the activities were perceived as useful, especially the reminders and safety checks, with possible benefits of companionship, reassurance and reduced caregiver burden. Suggestions included personalising the robot to each individual, simplifying the language and adding more activities. Technical issues still need to be fixed. - This study adds to knowledge about healthcare robots for people with MCI by developing and testing a new robot with daily-care activities including safety checks. The robot was seen to be potentially useful but needs to be tested with people with MCI. | New Zealand |
| **Lee et al., 2019** | Lee, M., Ackermans, S., van As, N., Chang, H., Lucas, E., and IJsselsteijn, W.  (2019). “Caring for vincent: a chatbot for self-compassion,” in Proceeding of the  CHI conference on human factors in computing systems proceedings (CHI 2019).  doi: 10.1145/3290605.3300932 | II- Qualitative study | Conversational agents (chatbots) in mental health care | - The digitization of mental health care holds promises of affordable and ubiquitously available treatment, e.g., with conversational agents (chatbots). While technology can guide people to care for themselves, this study examined how people can care for another being as a way to care for themselves. The study created a self-compassion chatbot (Vincent) and compared between caregiving and care-receiving conditions. Care-giving Vincent asked participants to partake in self-compassion exercises. Care-receiving Vincent shared its foibles, e.g., embarrassingly arriving late at an IP address, and sought out advice. - While self-compassion increased for both conditions, only those with care-receiving Vincent significantly improved. - Qualitative data on how participants interacted with Vincent shows that when a person cares for a chatbot, the person's self-compassion can be enhanced, which could be incorporated into future design to strengthen mental health with chatbots. | Netherlands |
| **Lee et al., 2021** | Lee, E. E., Torous, J., De Choudhury, M., Depp, C. A., Graham, S. A.,  Kim, H. C., et al. (2021). Artificial intelligence for mental health care: clinical  applications, barriers, facilitators, and artificial wisdom. Biol. Psychiatry Cogn.  Neurosci. Neuroimag. 6, 856–864. doi: 10.1016/j.bpsc.2021.02.001 | V- Conference | Artificial Intelligence for mental health care: clinical applications, barriers, facilitators, and artificial wisdom | - Given the high morbidity and mortality in people with psychiatric disorders, coupled with a worsening shortage of mental health care providers, there is an urgent need for AI to help identify high-risk individuals and provide interventions to prevent and treat mental illnesses. While published research on AI in neuropsychiatry is rather limited, there is a growing number of successful examples of AI's use with electronic health records, brain imaging, sensor-based monitoring systems, and social media platforms to predict, classify, or subgroup mental illnesses as well as problems such as suicidality. - This article is the product of a study group held at the American College of Neuropsychopharmacology conference in 2019. It provides an overview of AI approaches in mental health care, seeking to help with clinical diagnosis, prognosis, and treatment, as well as clinical and technological challenges, focusing on multiple illustrative publications. Although AI could help redefine mental illnesses more objectively, identify them at a prodromal stage, personalize treatments, and empower patients in their own care, it must address issues of bias, privacy, transparency, and other ethical concerns. These aspirations reflect human wisdom, which is more strongly associated than intelligence with individual and societal well-being. Thus, the future AI or artificial wisdom could provide technology that enables more compassionate and ethically sound care to diverse groups of people. | United States |
| **Levett-Jones et al., 2017** | Levett-Jones, T., Lapkin, S., Govind, N., Pich, J., Hoffman, K., Jeong, S. Y.,  et al. (2017). Measuring the impact of a ‘point of view’ disability simulation on  nursing students’ empathy using the comprehensive state empathy scale. Nurse  Educ. Today 59, 75–81. doi: 10.1016/j.nedt.2017.09.007 | VI- Design study | Immersive point-of-view simulation on nursing students' empathy towards people with an Acquired Brain Injury | - Participant (n=390) nursing students undertook the simulation in pairs and were randomly allocated to the role of either a person with Acquired Brain Injury or a rehabilitation nurse. The simulated 'patients' wore a hemiparesis suit that replicated the experience of dysphasia, hemianopia and hemiparesis. - On average, participants reported significantly higher mean empathy scores post simulation (3.75, SD=0.66) compared to pre simulation (3.38 SD=0.61); t (398)=10.33, p<0.001. However, this increase was higher for participants who assumed the role of a 'rehabilitation nurse' (mean=3.86, SD=0.62) than for those who took on the 'patient' role (mean=3.64, SD=0.68), p<0.001. - The results from this study attest to the potential of point-of-view simulations to positively impact nursing students' empathy towards people with a disability. Research with other vulnerable patient groups, student cohorts and in other contexts would be beneficial in taking this work forward. | Australia |
| **Lindner, 2021** | Lindner, P. (2021). Better, virtually: the past, present, and future of virtual reality  cognitive behavior therapy. J. Cogn. Ther. 14, 23–46. doi: 10.1007/s41811-020-  00090-7 | III- Narrative review | Review of the past, present, and future of virtual reality cognitive behaviour therapy | - Virtual reality (VR) is an immersive technology capable of creating a powerful, perceptual illusion of being present in a virtual environment. VR technology has been used in cognitive behaviour therapy since the 1990s and accumulated an impressive evidence base, yet with the recent release of consumer VR platforms came a true paradigm shift in the capabilities and scalability of VR for mental health. - This narrative review summarizes the past, present, and future of the field, including milestone studies and discussions on the clinical potential of alternative embodiment, gamification, avatar therapists, virtual gatherings, immersive storytelling, and more. Although the future is hard to predict, clinical VR has and will continue to be inherently intertwined with what are now rapid developments in technology, presenting both challenges and exciting opportunities to do what is not possible in the real world. | Sweden |
| **Ling et al., 2020** | Ling, J., Hong, J. C., Hayashi, Y., Yasuda, K., Kitaji, Y., Harashima, H., et al.  (2020). A haptic-based perception-empathy biofeedback system with vibration  transition: verifying the attention amount. Ann. Int. Conf. IEEE Eng. Med. Biol.  Soc. 2020, 3779–3782. doi: 10.1109/EMBC44109.2020.9176213 | VI- Design study | Explains biofeedback system to support patients with paralysis of the foot by feedback patters | - A perception-empathy biofeedback (PEBF) system is proposed that supplements the foot pressure status of a paralyzed foot with a wearable vibrotactile biofeedback (BF) vest to the back. Improvements in the ankle dorsiflexion and push-off movement in the swing phase and pre-swing phase, respectively, can be expected after using the proposed system. The 3 week pilot clinical tests suggest that significant improvement is only observed for the push-off movement. It is assumed that the attention required to recognize the BF was beyond the ability of the patients. In this paper, a dual task (40 s walking and performing mental arithmetic at the same time) was conducted with the following conditions: no vibrations and providing BF to the lower back and the entire back. According to the results, the ankle joint angle of the paralyzed side at push-off under the entire back condition is statistically significant (p = 0.0780); however, there are no significant changes under the lower back condition (p = 0.4998). Moreover, the ankle joint angle of the paralyzed side at the initial contact is statistically significant with respect to the lower back condition (p = 0.0233) and shows a significant trend for the entire back condition (p = 0.0730). The results suggest that the limited attention capacity of hemiplegic patients fails to improve both dorsiflexion and push-off movements; moreover, ankle motion can be promoted if attention is concentrated on recognizing focalized vibratory feedback patterns. | Japan |
| **Locsin, 2017** | Locsin, R. C. (2017). The co-existence of technology and caring in the theory of  technological competency as caring in nursing. J. Med. Invest. JMI 64, 160–164.  doi: 10.2152/jmi.64.160 | IV- Commentary | The co-existence of technology and caring in the theory of technological competency as caring in nursing | - Argues that the coexistence of technology and caring is best exemplified in nursing. The theory of Technological Competency as Caring in Nursing illuminates this coexistence as the essence of technology in health care premised on machine technologies as a generic concept of objects or things that are mechanical, organic, and electronic. With its timely development these technologies are continually imbued with artificial general intelligence. As such, the ultimate expression of machine technologies in nursing turns out to be autonomous robots (ARs) with future potentials of functions comparable to human persons. - Suggests that while theory-based nursing practice is essential to nursing care practice, quality human care, particularly with technologies assuming indispensable practice process mechanisms is critical. Some practice-based questions informing ARs and human person engagements in nursing care practice include, "Will ARs which are imbued with artificial intelligence replace nurses in their practice?" "What contributions to quality human health care will autonomous and artificially intelligent robots provide?" While these questions may reflect far-reaching ramifications of technologies in health care, it must also be acknowledged that these technologies are fundamental to the delivery of quality human health care now, and in the future. | Japan |
| **Loftus et al. 2020** | Loftus, T. J., Filiberto, A. C., Balch, J., Ayzengart, A. L., Tighe, P. J., Rashidi, P.,  et al. (2020). Intelligent, autonomous machines in surgery. J. Surg. Res. 253, 92–99.  doi: 10.1016/j.jss.2020.03.046 | IV- Commentary | Intelligent autonomous machines in surgery | - Surgeons perform two primary tasks: operating and engaging patients and caregivers in shared decision-making. Human dexterity and decision-making are biologically limited. Intelligent, autonomous machines have the potential to augment or replace surgeons. Argues that rather than regarding this possibility with denial, ire, or indifference, surgeons should understand and steer these technologies. - Closer examination of surgical innovations and lessons learned from the automotive industry can inform this process. Innovations in minimally invasive surgery and surgical decision-making follow classic S-shaped curves with three phases: (1) introduction of a new technology, (2) achievement of a performance advantage relative to existing standards, and (3) arrival at a performance plateau, followed by replacement with an innovation featuring greater machine autonomy and less human influence. - Argues that there is currently no level I evidence demonstrating improved patient outcomes using intelligent, autonomous machines for performing operations or surgical decision-making tasks. History suggests that if such evidence emerges and if the machines are cost effective, then they will augment or replace humans, initially for simple, common, rote tasks under close human supervision and later for complex tasks with minimal human supervision. This process poses ethical challenges in assigning liability for errors, matching decisions to patient values, and displacing human workers, but may allow surgeons to spend less time gathering and analyzing data and more time interacting with patients and tending to urgent, critical-and potentially more valuable-aspects of patient care. Surgeons should steer these technologies toward optimal patient care and net social benefit using the uniquely human traits of creativity, altruism, and moral deliberation. | United States |
| **Louie et al., 2018** | Louie, A. K., Coverdale, J. H., Balon, R., Beresin, E. V., Brenner, A. M., Guerrero,  A., et al. (2018). Enhancing empathy: a role for virtual reality? Acad. Psychiatry  J. Am. Assoc. Direct. Psychiatric Res. Train. Assoc. Acad. Psychiatry 42, 747–752.  doi: 10.1007/s40596-018-0995-2 | IV- Commentary | Evidence on the application and use of virtual reality technologies | - A nascent literature suggests that using virtual reality to enhance empathy may become a practical consideration, especially if mechanisms such as immersion and body transfer are effectively leveraged. Yet, many questions remain, and more critical study is needed. Major challenges include demonstrating the transferability of enhanced empathy outside the laboratory into real-world contexts and documenting the sustainability of empathy enhancement with a significant effect size. Future research might include blending virtual reality with debriefing protocols, determining the mediating role of individual differences among volunteers, testing for a ceiling effect, assessing adherence to use of virtual reality, measuring adverse effects, and more. If proven effective and safe, digital technologies like virtual reality may become a highly automated, cost-effective, and widely disseminated means of enhancing empathy skills. | United States |
| **Loveys et al., 2022** | Loveys, K., Sagar, M., Billinghurst, M., Saffaryazdi, N., and Broadbent, E.  (2022). “Exploring empathy with digital humans, 2022,” in Proceeding of the IEEE  conference on virtual reality and 3D user interfaces abstracts and workshops (VRW),  233–237. doi: 10.1109/VRW55335.2022.00055 | V- Conference paper | Exploring empathy with digital humans | - Digital humans are autonomously-animated virtual people whose social interactions are driven by artificial intelligence. They are increasingly being deployed in applications such as healthcare, customer service, and education, and they may have a place in the metaverse. For digital humans to have effective social relationships with users, it is important that they are capable of empathetic interactions. - This research aims to evaluate and build upon the autonomous empathy system of a digital human through five experimental studies. Psychological and physiological data will be collected, and the effects will be compared in an Augmented Reality environment and cross-culturally. This paper presents the research agenda and discusses considerations and challenges for empathetic interactions with digital humans. | New Zealand |
| **Lui and Sundar, 2020** | Lui, B., and Sundar, S. S. (2020). Should machines express sympathy and  empathy? Experiments with a health advice chatbot. Cyberpsychol Behav. Soc.  Netw. 21, 625–636. doi: 10.1089/cyber.2018.0110 | II- Research study | Explores whether machines (chatbots) should express sympathy or empathy | - Two experiments with a chatbot providing online medical information advice about a sensitive personal issue. In Study 1, participants (N=158) simply read a dialogue between a chatbot and a human user. In Study 2, participants (N=88) interacted with a real chatbot. The study tested the effect of three types of empathic expression sympathy, cognitive empathy, and affective empathy on individuals' perceptions of the service and the chatbot. - Results indicate that expression of sympathy and empathy is favoured over unemotional provision of advice, in support of the Computers are Social Actors (CASA) paradigm. This is particularly true for users who are initially sceptical about machines possessing social cognitive capabilities. | United States |
| **Ma et al., 2021** | Ma, Z., Huang, K. T., and Yao, L. (2021). Feasibility of a computer role-playing  game to promote empathy in nursing students: the role of immersiveness and  perspective. Cyberpsychol. Behav. Soc. Net. 24, 750–755. doi: 10.1089/cyber.2020.  0371 | VI- Design study | Virtual reality and computer role-playing games in nursing education | - Empathy is considered a cornerstone of high-quality health care and a required element of nursing education. Although computer role-playing games (CRPGs) are a promising tool to promote clinical empathy, little is known about how and why it is effective at improving empathy. The goal of the current study is to investigate the feasibility and effectiveness of a CRPG on nursing students' empathy with a focus on immersiveness and perspective. - Results from a 2 × 2 (virtual reality [VR] vs. non-VR × patient's family's perspective vs. health care provider's perspective) between-subjects experiment (N = 69) showed that playing the game in VR (vs. non-VR) led to greater spatial presence and empathy. Moreover, playing from the health care provider's (vs. patient's family's) perspective elicited greater empathy. A moderated mediation effect was found, suggesting that users' attention allocation significantly mediated the effect of immersiveness on empathy in the patient's family's perspective condition. These findings show the feasibility of using a role-playing game for nursing education. Theoretical and practical implications involving empathy training are discussed, along with suggestions for further research. | United States |
| **Majid et al., 2021** | Majid, S., Reeves, S., Figueredo, G., Brown, S., Lang, A., Moore, M., et al. (2021).  The extent of user involvement in the design of self-tracking technology for bipolar  disorder: literature review. JMIR Mental Health 8:e27991. doi: 10.2196/27991 | III- Literature review | Study to examine the current ways in which users are involved in the design and evaluation of self-monitoring apps for BD, investigating 3 specific questions: are users involved in the design and evaluation of technology? If so, how does this happen? And what are the best practice ingredients regarding the design of mental health technology? | - Explains that the number of self-monitoring apps for bipolar disorder (BD) is increasing. The involvement of users in human-computer interaction (HCI) research has a long history and is becoming a core concern for designers working in this space. The application of models of involvement, such as user-centered design, is becoming standardized to optimize the reach, adoption, and sustained use of this type of technology. - Out of the 11 novel smartphone apps included in this review, 4 (36%) self-monitoring apps were classified as having no mention of user involvement in design, 1 (9%) self-monitoring app was classified as having low user involvement, 4 (36%) self-monitoring apps were classified as having medium user involvement, and 2 (18%) self-monitoring apps were classified as having high user involvement. Despite the presence of extant approaches for the involvement of the user in the process of design and evaluation, there is large variability in whether the user is involved, how they are involved, and to what extent there is a reported emphasis on the voice of the user, which is the ultimate aim of such design approaches. - Asserts that users should be involved early in the design process, and this should not just be limited to the design itself, but also to associated research ensuring end-to-end involvement. Communities in health care-based design and HCI design need to work together to increase awareness of the different methods available and to encourage the use and mixing of the methods as well as establish better mechanisms to reach the target user group. | United Kingdom |
| **Marcoux et al., 2021** | Marcoux, A., Tessier, M. H., Grondin, F., Reduron, L., and Jackson, P. L. (2021).  Basic, clinical and social perspectives on the use of virtual characters in mental  health. Sante Ment Que. 46, 35–70. doi: 10.7202/1081509ar | VI- Design study | Explores the potential of virtual characters in mental healthcare practices and societal challenges regarding their use | - Basic studies highlight several characteristics of the virtual characters that seem to influence patient-clinician interactions. These characteristics can be classified into two categories: perceptual (e.g. realism) and social features (i.e. attribution of social categories such as gender). To this day, many interventions and/ or assessments using virtual characters have shown various levels of efficiency in mental health, and certain elements of a therapeutic relationship (e.g. alliance and empathy) may even be triggered during an interaction with a virtual character. To develop and increase the use of virtual characters, numerous socioeconomic and ethical issues must be examined. Although the accessibility and the availability of virtual characters are an undeniable advantage for their use in mental healthcare, some inequities about their application remain. In addition, the accumulation of biometric data (e.g. heart rate) could provide valuable information to clinicians and could help develop autonomous virtual characters, which raises concerns over issues of security and privacy. This paper proposes some recommendations to avoid such undesirable outcomes. | Canada |
| **Márquez Sánchez et al., 2020** | Márquez Sánchez, S., Mora-Simon, S., Herrera-Santos, J., Roncero, A. O., and  Corchado, J. M. (2020). Intelligent dolls and robots for the treatment of elderly  people with dementia. ADCAIJ Adv. Distrib. Comput. Artifi. Int. J. Regular Issue 9,  99–11. doi: 10.14201/ADCAIJ20209199112 | III-Review | Review of doll and robot therapy | - Dolls and robots are effective and beneficial non-pharmacological therapies applied in different clinical settings. Doll therapy (DT), principally based on Bowlby's attachment theory, uses an empathy or lifelike baby doll to awaken caring behaviours in patients. Robot therapies (RT) involve care robots that have a friendly attitude and appearance. They evoke different verbal, motor and emotional reactions in patients. Both DT and RT are person-centred therapies that provide patients with a realistic experience with the aim of improving their wellbeing. These therapies can be used in people suffering from different neurological, psychological and mental health disorders, such as Alzheimer's Disease, autism spectrum disorder, stress or depression. The characteristics of both therapies, their benefits and the possibilities for innovation in the therapeutic field are presented. | Spain |
| **McCarthy et al., 2020** | McCarthy, S., O’Raghallaigh, P., Woodworth, S., Lim, Y. Y., Kenny, L. C.,  and Adam, F. (2020). Embedding the pillars of quality in health information  technology solutions using “integrated patient journey mapping” (IPJM): case  study. JMIR Hum. Factors. 7:e17416. doi: 10.2196/17416 | II- Research study | In-depth case study of a design tool called Integrated Patient Journey Mapping (IPJM) that was developed to assist multidisciplinary teams in designing effective health information technology solutions and to promote empathy and the emergence of shared commitment and understanding among multidisciplinary teams | - Health information technology (HIT) and associated data analytics offer significant opportunities for tackling some of the more complex challenges currently facing the health care sector. However, to deliver robust health care service improvements, it is essential that HIT solutions be designed by parallelly considering the 3 core pillars of health care quality: clinical effectiveness, patient safety, and patient experience. This requires multidisciplinary teams to design interventions that both adhere to medical protocols and achieve the tripartite goals of effectiveness, safety, and experience. - A case study of the use of the integrated patient journey mapping (IPJM) tool during Learning to Evaluate Blood Pressure at Home (LEANBH), a connected health project that developed an HIT solution for the perinatal health context. Data were collected from over 700 hours of participant observations and 10 semi structured interviews. - IPJM offered a constructive tool for multidisciplinary teams to work together in designing an HIT solution, through mapping the physical and emotional journey of patients for both the current service and the proposed connected health service. This allowed team members to consider the goals, tasks, constraints, and actors involved in the delivery of this journey and to capture requirements for the digital touchpoints of the connected health service. | Republic of Ireland |
| **Michael et al., 2019** | Michael, S. H., Villarreal, P. M., Ferguson, M. F., Wiler, J. L., Zane, R. D.,  and Flarity, K. (2019). Virtual reality-based resilience programs: feasibility and  implementation for inpatient oncology nurses. Clin. J. Oncol. Nurs. 23, 664–667.  doi: 10.1188/19.CJON.664-667 | VI- Design evaluation study | Virtual reality technology intervention for oncology nurses to reduce stress and support wellbeing | - Virtual reality (VR) is an emerging technology that has been applied in health-care education and training and is being explored as an intervention to reduce stress and support wellness for healthcare providers. This article reviews recommendations from an implementation project about a VR intervention for oncology nurses. | United States |
| **Milcent et al., 2021** | Milcent, A., Kadri, A., and Richir, S. (2021). Using facial expressiveness of a  virtual agent to induce empathy in users. Int. J. Hum. Comput. Int. 38, 240–252.  doi: 10.1080/10447318.2021.1938387 | VI- Design study | Using facial expressiveness of a virtual agent to induce empathy in users | - Healthcare simulators are learning environments that offer many training opportunities. The integration of expressive virtual patients in these simulators encourages the exchanges and provokes emotional reactions in the learner, which promotes memorization and learning. - Explores whether the facial expressiveness of a virtual agent is a factor to be considered in order to improve the user experience. - Results show a high empathy score when users train with simulators. Depending on the context, there is a significant difference in perspective-taking in favour of the users who interact with an expressive virtual agent to a virtual agent without facial expressiveness. | France |
| **Miloff et al., 2020** | Miloff, A., Carlbring, P., Hamilton, W., Andersson, G., Reuterskiöld, L., and  Lindner, P. (2020). Measuring alliance toward embodied virtual therapists in the  era of automated treatments with the virtual therapist alliance scale (VTAS):  development and psychometric evaluation. J. Med. Int. Res. 22:e16660. doi: 10.  2196/16660 | VI- Design study | Measuring alliance toward embodied virtual therapists with the Virtual Therapist Alliance Scale (VTAS) | - Automated virtual reality exposure therapies (VRETs) are self-help treatments conducted by oneself and supported by a virtual therapist embodied visually and/or with audio feedback. This simulates many of the nonspecific relational elements and common factors present in face-to-face therapy and may be a means of improving adherence to and efficacy of self-guided treatments. However, little is known about alliance toward the virtual therapist, despite alliance being an important predictor of treatment outcome. - Aimed to evaluate the first alliance instrument developed for use with embodied virtual therapists in an automated treatment format-the Virtual Therapist Alliance Scale (VTAS)-by (1) assessing its psychometric properties, (2) verifying the dimensionality of the scale, and (3) determining the predictive ability of the scale with treatment outcome. - A psychometric evaluation and exploratory factor analysis of the VTAS was conducted using data from two samples of spider-fearful patients treated with VRET and the help of an embodied, voice-based virtual therapist (n=70). Multiple regression models and bivariate correlations were used to assess the VTAS relationship with treatment outcome, according to self-reported fear and convergence with presence and user-friendliness process measures. - The VTAS showed a sound two-factor solution composed of a primary factor covering task, goal, and copresence; adequate internal consistency; and good convergent validity, including moderate correlation (r=.310, P=.01) with outcomes over follow-up. - These preliminary results suggest that alliance toward a virtual therapist is a significant predictor of treatment outcome, favors the importance of a task-goal over bond-factor, and should be explored in studies with larger sample sizes and in additional forms of embodiment. | Sweden |
| **Mirkovic et al., 2018** | Mirkovic, J., Jessen, S., Kristjansdottir, O. B., Krogseth, T., Koricho, A. T.,  and Ruland, C. M. (2018). Developing technology to mobilize personal strengths  in people with chronic illness: positive codesign approach. JMIR Format. Res.  2:e10774. doi: 10.2196/10774 | II- Research study | Reports on a codesigning workshop that was organised with the aim to explore user requirements and ideas for how technology can be used to help people with chronic illness activate their personal strengths in managing their everyday challenges | - Emerging research from psychology and the bio-behavioural sciences recognizes the importance of supporting patients to mobilize their personal strengths to live well with chronic illness. Positive technology and positive computing could be used as underlying design approaches to guide design and development of new technology-based interventions for this user group that support mobilizing their personal strengths. - Thirty-five participants from diverse backgrounds (patients, health care providers, designers, software developers, and researchers) participated. The workshop combined principles of (1) participatory and service design to enable meaningful participation and collaboration of different stakeholders and (2) an appreciative inquiry methodology to shift participants' attention to positive traits, values, and aspects that are meaningful and life-giving and stimulate participants' creativity, engagement, and collaboration. Utilizing these principles, participants were engaged in group activities to develop ideas for strengths-supportive tools. Each group consisted of 3-8 participants with different backgrounds. All group work was analysed using thematic analyses. - Reports that participants were highly engaged in all activities and reported a wide variety of requirements and ideas, including more than 150 personal strength examples, more than 100 everyday challenges that could be addressed by using personal strengths, and a wide range of functionality requirements (eg, social support, strength awareness and reflection, and coping strategies). 6 concepts for strength-supportive tools were created. These included the following: a mobile app to support a person to store, reflect on, and mobilize one's strengths (Strengths treasure chest app); "empathy glasses" enabling a person to see a situation from another person's perspective (Empathy Simulator); and a mobile app allowing a person to receive supportive messages from close people in a safe user-controlled environment (Cheering squad app). Suggested design elements for making the tools engaging included: metaphors (eg, trees, treasure island), visualization techniques (eg, dashboards, color coding), and multimedia (eg, graphics). Maintaining a positive focus throughout the tool was an important requirement, especially for feedback and framing of content. - Concludes that combining participatory, service design, and appreciative inquiry methods were highly useful to engage participants in creating innovative ideas. Building on peoples' core values and positive experiences empowered the participants to expand their horizons from addressing problems and symptoms, which is a very common approach in health care today, to focusing on their capacities and that which is possible, despite their chronic illness. The ideas and user requirements, combined with insights from relevant theories (eg, positive technology, self-management) and evidence from the related literature, are critical to guide the development of future more personalized and strengths-focused self-management tools. | Norway |
| **Mitchell et al., 2011** | Mitchell, S., Heyden, R., Heyden, N., Schroy, P., Andrew, S., Sadikova, E., et al.  (2011). A pilot study of motivational interviewing training in a virtual world.  J. Med. Int. Res. 13:e77. doi: 10.2196/jmir.1825 | VI- Design study | Study to explore the feasibility, acceptability, and effectiveness of a virtual-world platform for delivering motivational interviewing (MI) training designed for physicians and pilot test instructional designs using SL for MI training | - Motivational interviewing (MI) is an evidence-based, patient-centered counselling strategy proven to support patients seeking health behaviour change. Yet the time and travel commitment for MI training is often a barrier to the adoption of MI by health care professionals. - Virtual worlds such as Second Life (SL) are rapidly becoming part of the educational technology landscape and offer not only the potential to improve access to MI training but also to deepen the MI training experience through the use of immersive online environments. Despite SL's potential for medical education applications, little work is published studying its use for this purpose and still less is known of educational outcomes for physician training in MI using a virtual-world platform. - Design and pilot test of an MI training program in the SL virtual world with13 primary care physicians in a two-session, interactive program in SL on the use of MI for counselling patients about colorectal cancer screening. - The SL learning environment was highly rated, with 77% (n = 10) of the doctors reporting SL to be an effective educational medium. Learners' confidence and clinical practice patterns for colorectal cancer screening improved after training. The results suggest that virtual worlds offer the potential for a new medical education pedagogy that will enhance learning outcomes for patient-centered communication skills training. | United States |
| **Montayre, 2018** | Montayre, J. (2018). Nursing the future: braving possibilities, challenges and  dilemmas. Nurs. Praxis New Zealand 34, 5–6. doi: 10.36951/NgPxNZ.2018.001 | IV- Editorial | Perspective of future nursing in the age of AI technologies | - Discusses futuristic compassionate nursing care that will be delivered effectively through technology including the increasing popularity of virtual clinics, and possibly eventually nurse-led virtual clinics globally. Assessing clients through mobile screen technology. While virtual clinics are becoming more common as part of the health care landscape, nurses have yet to be comprehensively upskilled and prepared on how to perform an accurate 'virtual assessment'. - Argues that because virtual proximity depends highly on technology, it requires nurses as well as clients to have acquired knowledge to operate and respond to this platform. The essential navigational skills towards technology may pose challenges and dilemmas for nurses to achieve a technology-driven humane and compassionate connection. Discusses how other initiatives include bio-printed 3D tissues and organs, augmented reality, wearable monitoring devices and drones delivering vaccines are likely to change nursing. | New Zealand |
| **Montemayor et al., 2021** | Montemayor, C., Halpern, J., and Fairweather, A. (2021). In principle obstacles  for empathic AI: why we can’t replace human empathy in healthcare. AI Soc. [Epub  ahead of print]. doi: 10.1007/s00146-021-01230-z | IV- Commentary | What are the limits of the use of artificial intelligence (AI) in the relational aspects of medical and nursing care? | - Argues that there are also in principle obstacles to the application of AI in clinical medicine and care where empathy is important, and that these problems cannot be solved with any of the technical and theoretical approaches that shape the current application of AI in specific areas of clinical medicine in which care for patients is fundamental. This is important, because it generates specific risks that may be overlooked otherwise, and it justifies the necessity of human monitoring and emotional intervention in clinical medicine. Consequently, difficult issues concerning moral and legal responsibility may ensue if these in principle problems are ignored. | United States |
| **Moosaei et al., 2017** | Moosaei, M., Das, S. K., Popa, D. O., and Riek, L. D. (2017). “Using facially  expressive robots to calibrate clinical pain perception,” in Proceeding of the 2017  12th ACM/IEEE international conference on human-robot interaction, HRI, 32–41.  doi: 10.1145/2909824.3020216 | V- Conference paper | Explores people’s ability to interpret expressions of pain on a humanoid robot and virtual avatar | - Introduced a novel application of social robotics in healthcare: high fidelity, facially expressive, robotic patient simulators (RPSs), and explore their usage within a clinical experimental context. Current commercially-available RPSs, the most commonly used humanoid robots worldwide, are substantially limited in their usability and fidelity due to the fact that they lack one of the most important clinical interaction and diagnostic tools: an expressive face. - Using autonomous facial synthesis techniques, the study synthesized pain both on a humanoid robot and comparable virtual avatar. Experiment with 51 clinicians and 51 laypersons (n = 102), to explore differences in pain perception across the two groups, and also to explore the effects of embodiment (robot or avatar) on pain perception. - Results suggest that clinicians have lower overall accuracy in detecting synthesized pain in comparison to lay participants. All participants are overall less accurate detecting pain from a humanoid robot in comparison to a comparable virtual avatar, lending support to other recent findings in the HRI community. This research ultimately reveals new insights into the use of RPSs as a training tool for calibrating clinicians' pain detection skills. | United States |
| **Nadin, 2020** | Nadin, M. (2020). Aiming AI at a moving target: health (or disease). AI Soc.  2020, 1–9. doi: 10.1007/s00146-020-00943-x | IV- Commentary | Discussion of the future of AI in medicine | - Argues that spectacular achievements facilitated through applied deep learning methodology (based on neural networks), the "Everything is possible" view dominates the present "boom and bust" curve of AI performance. The optimistic view collides head on with the "It is not possible"- assertions often originating in a skewed understanding of both AI and medicine. The meaning of the conflicting views can be assessed only by addressing the nature of medicine. Specifically: Which part of medicine, if any, can and should be entrusted to AI-now or at some moment in the future? AI or not, medicine should incorporate the anticipation perspective in providing care. | United States |
| **Navarrete et al., 2021** | Navarrete, J., Martínez-Sanchis, M., Bellosta-Batalla, M., Baños, R., Cebolla, A.,  and Herrero, R. (2021). Compassionate embodied virtual experience increases the  adherence to meditation practice. Appl. Sci. 11:1276. doi: 10.3390/app11031276 | II- Research study | Study to evaluate the effectiveness of an embodied-VR system in generating a compassionate response and increasing the quality and adherence to meditation practice | - Health professionals or healthcare students (n=41) were randomly assigned to a regular audio guided meditation or to a meditation supported by an embodied-VR system, "The machine to be another". In both conditions, there was an initial in-person session and two weeks of meditation practice at home. An implicit measure was used to measure prosocial behaviour, and self-report questionnaires were administered to assess compassion related constructs, quality of meditation, and frequency of meditation. - Results revealed that participants from the embodied-VR condition meditated for double the amount of time at home than participants who only listened to the usual guided meditation. However, there were no significant differences in the overall quality of at-home meditation. This study shows that embodied-VR systems are useful for increasing adherence to meditation practice. | Spain |
| **Nisha et al., 2019** | Nisha, N., Iqbal, M., and Rifat, A. (2019). The changing paradigm of health and  mobile phones: an innovation in the health care system. J. Glob. Inf. Manag. 27,  19–46. | III- Review | Examines underlying factors that can influence future use intentions of m-Health services | - Mobile health services (m-Health) act as an effective, accessible and affordable means of providing healthcare knowledge to users directly from providers. Despite such benefits of m-Health services, rapid adoption is not yet occurring, particularly in emerging markets. The main barrier is mostly the cynical behaviour of users regarding this medium of healthcare services. - Conceptual model of the study identifies service qualities like reliability, privacy, responsiveness, empathy and information quality along with facilitating conditions, trust, effort expectancy and performance expectancy as significant constructs that influences users' overall perceptions of m-Health services, along with moderating effects of age and gender. | Bangladesh |
| **O'Gara et al., 2022** | O’Gara, G., Murray, L., Georgopoulou, S., Anstiss, T., Macquarrie, A.,  Wheatstone, P., et al. (2022). Safe space: what is the feasibility and acceptability  of a codesigned virtual reality intervention, incorporating compassionate mind  training, to support people undergoing cancer treatment in a clinical setting? BMJ  Open. 12:e047626. doi: 10.1136/bmjopen-2020-047626 | VI- Design study | Feasibility and acceptability of a codesigned virtual reality intervention (SafeSpace) incorporating compassionate mind training, to support people undergoing cancer treatment in a clinical setting | - The SafeSpace study codesigned and tested a virtual reality (VR) intervention, incorporating relaxation and compassionate mind training to determine acceptability/feasibility in an oncology setting and evaluate impact on physical/psychological well-being and quality of life. - Patient participants found the intervention acceptable and highlighted areas for development. The intervention is acceptable and feasible and has shown positive effects on mental well-being/stress in the oncology setting. | United Kingdom |
| **Oh et al., 2017** | Oh, K. J., Lee, D., Ko, B., Hyeon, J., and Choi, H. J. (2017). Empathy bot:  conversational service for psychiatric counseling with chat assistant. Stud. Health  Technol. Inform. 245:1235. | VI- Design study | Empathy Bot: conversational service for psychiatric counselling with chat assistant | - Proposes an intelligent assistant for psychiatric counselling that understands dialogues using high-level features of natural language understanding, and multi-modal emotion recognition. A response generation model using machine leaning provides suitable responses for clinical psychiatric counselling. | Korea |
| **Osis, 2021** | Osis, F. (2021). “Inform the head, give dexterity to the hand, familiarise the  heart”: seeing and using digitised eighteenth-century specimens in a modern  medical curriculum. Adv. Exp. Med. Biol. 1317, 163–179. doi: 10.1007/978-3-030-  61125-5_9 | IV- Chapter | Digitising anatomical and pathological specimens found in medical museums for education and learning | - Using digital replicas of historic specimens to teach anatomy also opens up a unique opportunity to educate students in the medical humanities in a fully integrated way. Understanding the full story of the specimens they use allows students to place themselves, their dissection subjects, and healthcare as a whole in a historical context. As well as fostering empathy in the dissection lab, the stories behind the specimens can be used to introduce key humanities topics, including ethics, institutional bias, and social aspects of health and disease. It is essential that this potential is explored now while digital anatomy is still a relatively young field, and therefore collaborations between anatomists and medical humanities practitioners can be built and included from the ground up. | United Kingdom |
| **Ostherr, 2022** | Ostherr, K. (2022). Artificial intelligence and medical humanities. J. Med. Hum.  43, 211–232. doi: 10.1007/s10912-020-09636-4 | IV- Commentary | The role of medical humanities in research into artificial intelligence technologies | - This article explains four key areas of concern relating to AI and the role that medical/health humanities research can play in addressing them: definition and regulation of "medical" versus "health" data and apps; social determinants of health; narrative medicine; and technological mediation of care. Issues include data privacy and trust, flawed datasets and algorithmic bias, racial discrimination, and the rhetoric of humanism and disability. | United States |
| **Palanica et al., 2019** | Palanica, A., Flaschner, P., Thommandram, A., Li, M., and Fossat, Y. (2019).  Physicians’ perceptions of chatbots in health care: cross-sectional web-based  survey. J. Med. Int. Res. 21:12887. doi: 10.2196/12887 | II- Research study | Study of technology-mediated symptom transference for transmitting an individual patient’s actual experience, rather than a simulation, to the user-a process termed “tele-empathy” | - An investigational digital tele-empathy device for use toward patients with Parkinson’s disease (PD), known as SymPulse™. The device plays back muscle tremors using an armband, giving the wearer a replication of the involuntary muscle activity that a patient with PD feels. The purpose of the current study was to determine whether the SymPulse™ device could enhance feelings of empathy in test participants (wearing the device) versus control participants (not wearing the device). - A sample of 45 participants (22 test; 23 control) reported their level of empathy via self-report questionnaires. Results revealed significantly higher empathy scale scores for test compared to control participants, demonstrating the effectiveness of the SymPulse™ for use in tele-empathy. The use of such technology for eliciting tele-empathy may have practical and clinical implications for providing effective training to health-care providers. | Canada |
| **Palmer and Schwan, 2022** | Palmer, A., and Schwan, D. (2022). Beneficent dehumanization: employing  artificial intelligence and carebots to mitigate shame-induced barriers to medical  care. Bioethics. 36, 187–193. doi: 10.1111/bioe.12986 | IV- Discussion | Employing artificial intelligence and carebots to mitigate shame-induced barriers to medical care | - Argues that medical ethicists have long expressed concerns that AI/carebot technologies remove the human element from medicine, resulting in dehumanization and depersonalized care. However, where shame presents a barrier to medical care, it is sometimes ethically permissible and even desirable to deploy AI/carebots because (i) dehumanization in medicine is not always morally wrong, and (ii) dehumanization can sometimes better promote and protect important medical values. Shame is often a consequence of the human-to-human element of medical care and can prevent patients from seeking treatment and from disclosing important information to their healthcare provider. Conditions and treatments that are shame-inducing offer opportunities for introducing AI/carebots in a manner that removes the human element of medicine but does so ethically for ‘beneficent dehumanization’. - Outlines numerous examples of shame-inducing interactions and how they are overcome by implementing existing and expected developments of AI/carebot technology that remove the human element from care. | United States |
| **Panzarasa et al, 2020** | Panzarasa, P., Griffiths, C. J., Sastry, N., and De Simoni, A. (2020). Social medical  capital: how patients and caregivers can benefit from online social interactions.  J. Med. Int. Res. 22:e16337. doi: 10.2196/16337 | IV- Discussion | Explores how patients and caregivers can benefit from online social interactions and develops the notion of social medical capital | - The rapid growth of online health communities and the increasing availability of relational data from social media provide invaluable opportunities for using network science and big data analytics to better understand how patients and caregivers can benefit from online conversations. Offers a new network-based theory of social medical capital that will open up new avenues for conducting large-scale network studies of online health communities and devising effective policy interventions aimed at improving patients' self-care and health. | United Kingdom |
| **Papadakos et al., 2017** | Papadakos, J., Trang, A., Cyr, A. B., Abdelmutti, N., Giuliani, M. E., Snow, M.,  et al. (2017). Deconstructing cancer patient information seeking in a consumer  health library toward developing a virtual information consult for cancer patients  and their caregivers: a qualitative, instrumental case study. JMIR Cancer 3:e6.  doi: 10.2196/cancer.6933 | II- Qualitative research | Study of information seeking behaviours of cancer patients and caregivers and whether these information needs can be met by a virtual information consultant (web app) | - It is possible to replicate library functions in a Web app with a few exceptions that cannot be replicated online. These elements include access to journal articles or other content behind paywalls and the librarian's ability to encourage further discussion through empathy and active listening. Discussion with the librarian could serve to refine and predict needs through observing information seekers and to provide immediate connection to spiritual care and psychosocial support for patrons in distress. | Canada |
| **Patel et al., 2020** | Patel, D., Hawkins, J., Chehab, L. Z., Martin-Tuite, P., Feler, J., Tan, A., et al.  (2020). Developing virtual reality trauma training experiences using 360-degree  video: tutorial. J. Med. Int. Res. 22:e22420. doi: 10.2196/22420 | VI- Design study | Developing virtual reality trauma training experiences using 360-degree video | - Virtual reality (VR), and in particular, the use of 360-degree video and audio (cineVR), is the next-generation advancement in medical simulation that has novel applications to augment clinical skill practice, empathy building, and team training. - Describes methods to design and develop a cineVR medical education curriculum for trauma care training using real patient care scenarios at an urban, safety-net hospital and Level 1 trauma center. Details the process of finding a cineVR production partner; choosing the camera perspectives; maintaining patient, provider, and staff privacy; ensuring data security; executing the cineVR production process; and building the curriculum. | United States |
| **Pedersen et al., 2018** | Pedersen, I., Reid, S., and Aspevig, K. (2018). Developing social robots for  aging populations: a literature review of recent academic sources. Soc. Compass.  12:e12585. doi: 10.1111/soc4.12585 | III- Literature review | Developing social robots for aging populations | - The perception of aging populations is a major factor driving the social robot development movement. A growing body of research reflects the expanding interest in social robots. This paper synthesizes research on the development of social robots. Themes: (a) robots as an aid in treatment; (b) robots as social assistants and home companions; and (c) robots as custodial caregivers that are viewed in terms of ethical implications. This paper outlines the issues surrounding social, commitment, assistive, and companion robots for use in medical treatment, mental health therapy, physiotherapy, care facilities, and private homes. It describes some of the ethical concerns raised by researchers and media, including questions of control, privacy, consent, and the issue of simulated versus human compassion in caregiving. - Argues that a rhetoric of urgency concerning aging populations drives the development of robots, which frames citizens who will benefit from robots in reductive ways. Suggests that the contribution of humanities and social science research, including age studies and critical gerontology, should be better integrated with discourses of social robot development, largely from technical fields. | Canada |
| **Pepito et al., 2020** | Pepito, J. A., Ito, H., Betriana, F., Tanioka, T., and Locsin, R. C. (2020).  Intelligent humanoid robots expressing artificial humanlike empathy in nursing  situations. Nurs Philos. 21:e12318. doi: 10.1111/nup.12318 | IV- Discussion | Intelligent humanoid robots expressing artificial humanlike empathy in nursing situations | - Intelligent humanoid robots (IHRs) are becoming likely to be integrated into nursing practice. However, a proper integration of IHRs requires a detailed description and explanation of their essential capabilities, particularly regarding their competencies in replicating and portraying emotive functions such as empathy. - Existing humanoid robots can exhibit rudimentary forms of empathy; as these machines slowly become commonplace in healthcare settings, they will be expected to express empathy as a natural function, rather than merely to portray artificial empathy as a replication of human empathy. - Considers the impact of artificial empathy in nursing and, secondly, to describe the influence of Affective Developmental Robotics (ADR) in anticipation of the empathic behaviour presented by artificial humanoid robots. The ADR has demonstrated that it can be one means by which humanoid nurse robots can achieve expressions of more relatable artificial empathy. This will be one of the vital models for intelligent humanoid robots currently in nurse robot development for the healthcare industry. A discussion of IHRs demonstrating artificial empathy is critical to nursing practice today, particularly in healthcare settings dense with technology. | Philippines |
| **Plotzky et al., 2021** | Plotzky, C., Lindwedel, U., Sorber, M., Loessl, B., König, P., Kunze, C., et al.  (2021). Virtual reality simulations in nurse education: a systematic mapping  review. Nurse Educ. Today 101:104868. doi: 10.1016/j.nedt.2021.104868 | III- Scoping review | Review of literature on educational virtual reality nursing simulations and to analyse approaches from didactic and technical perspectives | - There is a large variety in the use and definition of VR simulation for educational purposes. Simulations were classified into four main educational objectives: procedural skills training to improve technical knowledge and proficiency; emergency response training that focusses on confidence; soft skills training that teaches empathy; and finally, psychomotor skills training. Various approaches and simulation designs were implemented to achieve these educational outcomes. A few of them were highly innovative in providing an immersive experience to learn complex tasks, e.g. auscultation, or foster empathy by mimicking life with dementia. - Despite an increase in the use of state-of-the-art VR nursing simulations, there is still a paucity of studies on immersive HMD based VR scenarios. | Germany |
| **Portz et al., 2020** | Portz, J. D., Ford, K. L., Doyon, K., Bekelman, D. B., Boxer, R. S., Kutner,  J. S., et al. (2020). Using grounded theory to inform the human-centered design  of digital health in geriatric palliative care. J. Pain Symptom Manage. 60, 1181–  1192.e1. doi: 10.1016/j.jpainsymman.2020.06.027 | II- Research study | A qualitative study examining patient, social convoy, and health care provider perspectives on digital health for palliative care to inform the design of future digital solutions for older adults with serious illness and their social convoy | - Digital health offers innovative mechanisms to engage in palliative care, yet digital systems are typically designed for individual users, rather than integrating the patient's caregiving "social convoy'' (i.e., family members, friends, neighbours, formal caregiving supports) to maximize benefit. As older adults with serious illness increasingly rely on the support of others, there is a need to foster effective integration of the social convoy in digitally supported palliative care. - Thematic results aligned with the human-centered design framework, which is a participatory approach to the design process that incorporates multiple user stakeholders to develop health solutions. The human-centered design process and corresponding theme included the following: 1) Empathy: Patient, Caregiver, and Provider Experience reports participants' experience with managing serious illness, caregiving, social support, and technology use. 2) Define: Reactions to Evidence-Based Care Concepts and Barriers illustrates participants' perspectives on the domains of palliative care ranging from symptom management to psychosocial-spiritual care. 3) Ideation: Desired Features reports participant recommendations for designing digital health tools for palliative care domains. | United States |
| **Powell, 2019** | Powell, J. (2019). Trust me, I’m a chatbot: how artificial intelligence in health  care fails the turing test. J. Med. Int. Res. 21:e16222. doi: 10.2196/16222 | IV- Opinion piece | Discussion of application of the Turing test to user-facing artificial intelligence systems in health care | - Medical decisions require value judgements, and the doctor-patient relationship requires empathy and understanding to arrive at a shared decision, often handling large areas of uncertainty and balancing competing risks. - Artificial intelligence therefore needs to supplement rather than replace medical professionals and identifying the complementary positioning of artificial intelligence in medical consultation is a key challenge for the future. | United Kingdom |
| **Price, 2013** | Price, A. (2013). Caring and technology in an intensive care unit: an  ethnographic study. Nurs. Crit. Care 18, 278–288. | II-Ethnographic study | Caring and technology in an intensive care unit | - An overarching theme of the crafting process was developed with sub themes of vigilance, focus of attention, being present and expectations with the ultimate goal of achieving the best interests for the individual patient. - Further research to detail more specifically how these areas are measured within critical care may be useful. - Identifies aspects that help and hinder caring in the technical setting to inform training. | United Kingdom |
| **Pulman et al. 2013** | Pulman, A., Taylor, J., Galvin, K., and Masding, M. (2013). Ideas and  enhancements related to mobile applications to support type 1 diabetes. JMIR  Mhealth Uhealth. 1:e12. doi: 10.2196/mhealth.2567 | II- Qualitative research | To develop insight into young people with type 1 diabetes and their current use of Web and mobile technology and its potential impact on health-related quality of life (HRQOL) | - Outlines the non-prototyped suggestions from 9 young people interviewees and argues that young people with type 1 diabetes have a key role to play in the design and implementation of new technology to support them and improve HRQOL. It is vital to include and reflect on their suggestions as they have a radically different view of technology than either their parents or practitioners. Argues designers need to consider the relationship to technology that young people with type 1 diabetes have, and then reflect on how this might make a difference to them and when it might not be a suitable mechanism to use. | United Kingdom |
| **Rahim et al., 2021** | Rahim, A. I., Ibrahim, M. I., Musa, K. I., Chua, S., and Yaacob, N. (2021).  Assessing patient-perceived hospital service quality and sentiment in malaysian  public hospitals using machine learning and facebook reviews. Int. J. Environ. Res.  Public Health 2021:18. | II- Research study | Study to investigate the determinants of positive sentiment expressed in hospital Facebook reviews | - Reports on development of machine learning to build a sentiment analyser and service quality (SERVQUAL) classifier that automatically classifies the sentiment and SERVQUAL dimensions. - Argues that Facebook reviews powered by machine learning algorithms provide valuable, real-time data that may be missed by traditional hospital quality assessments. Additionally, online patient reviews offer an untapped indication of quality that may benefit all healthcare stakeholders. | Malaysia |
| **Raja et a., 2021** | Raja, M., Bjerkan, J., Kymre, I. G., Galvin, K. T., and Uhrenfeldt, L. (2021).  Telehealth and digital developments in society that persons 75 years and older  in European countries have been part of: a scoping review. BMC Health Serv. Res.  21:1157. doi: 10.1186/s12913-021-07154-0 | III- Scoping review | Scoping review to map relevant evidence about telehealth and digital developments in society involving citizens aged 75 and over in European countries. | - Explains that demographic changes are leading to an ageing population in Europe. People are becoming more dependent on digital technologies and health ministries invest increasingly in digitalisation. Societal digital demands impact older people and learning to use new telehealth systems and digital devices are seen as a means of securing their needs. - This review focuses on older people’s experiences and the main barriers to, and facilitators of, societal digital demands. Reviews 13 sources which met the inclusion criteria (9 original study articles, 2 theses, 1 letter about a product and 1 project report). Few of the studies identified have investigated European citizens 75 years and older separately. The studies included varied in their design, location and focus. Older people have experienced both telehealth and digital devices making life easier and the opposite. The outstanding facilitator found was that technology should be easy to use, and difficulty in remembering the instructions was seen as an important barrier. Interestingly, both social support and lack of social support were found as facilitators of using new devices. - Telehealth may give a sense of security but learning to use a new device often takes extra effort. Older people were more open to new devices if the possible advantages of the new technology outweighed the effort that would be involved in adopting a new strategy. As technology develops rapidly, and life expectancy in Europe is anticipated to rise continually, there is a need for new and additional research among older European citizens. Future research should cover the technical solutions most relevant to older people today, social support and participants` access to the devices. | Norway |
| **Raman and McClelland, 2019** | Raman, R., and McClelland, L. E. (2019). Bringing compassion into information  systems research: a research agenda and call to action. J. Inform. Technol. 34, 2–21. | IV- Discussion | Discussion of the compassion and financial gain as the basis for information and communication technologies | - Asserts that compassion-driven approaches are the sustainable way for information and communication technologies to contribute to economic value. Suggests that future information systems research should emphasize the joint goals of compassion and financial gains from information and communication technologies. - Presents a broad agenda for future information systems research based on the valuing of both compassion and financial gains. Discusses how certain core assumptions underlying traditional information systems research-so far, driven primarily by economic value as outcome-would need to change in order to support this new agenda emphasizing compassion and economic value as complementary and synergistic outcomes. | United States |
| **Ramanayake et al., 2022** | Ramanayake, R.,Wicke, P., and Nallur, V. (2022). Immune moral models? Prosocial  rule breaking as a moral enhancement approach for ethical AI. AI Soc. 2022,  1–13. doi: 10.1007/s00146-022-01478-z | II- Research study | Examines rule breaking in humans and ethical implications for the design of AI technologies | - Envisages a future where Artificial Intelligence (AI) based agents make many decisions on behalf of humans. From healthcare decision-making to social media censoring, these agents face problems, and make decisions with ethical and societal implications. Ethical behaviour is a critical characteristic that we would like in a human-centric AI. A common observation in human-centric industries, like the service industry and healthcare, is that their professionals tend to break rules, if necessary, for pro-social reasons. This behaviour among humans is defined as pro-social rule breaking. To make AI agents more human-centric, we argue that there is a need for a mechanism that helps AI agents identify when to break rules set by their designers. To understand when AI agents need to break rules, we examine the conditions under which humans break rules for pro-social reasons. - Presents a study that introduces a 'vaccination strategy dilemma' to human participants and analyses their response. In this dilemma, one needs to decide whether they would distribute COVID-19 vaccines only to members of a high-risk group (follow the enforced rule) or, in selected cases, administer the vaccine to a few social influencers (break the rule), which might yield an overall greater benefit to society. - The results of the empirical study suggest a relationship between stakeholder utilities and pro-social rule breaking (PSRB), which neither deontological nor utilitarian ethics completely explain. Finally, the paper discusses the design characteristics of an ethical agent capable of PSRB and the future research directions on PSRB in the AI realm. The findings can inform the design of future AI agents, and their decision-making behaviour. | Ireland |
| **Riches et al., 2022** | Riches, S., Iannelli, H., Reynolds, L., Fisher, H. L., Cross, S., and Attoe, C. (2022).  Virtual reality-based training for mental health staff: a novel approach to increase  empathy, compassion, and subjective understanding of service user experience.  Adv. Simulat. 7:19. doi: 10.1186/s41077-022-00217-0 | IV- Commentary | Explores the use and benefits of virtual reality-based training for mental health staff, which could have important consequences in terms of improved staff empathy and reductions in harmful restrictive practices | - Explains that mental health service users report that staff empathy is key to developing positive therapeutic relationships but promoting empathy in staff training is challenging. Staff may struggle to maintain their compassion, particularly in challenging settings, and have limited clinical confidence when treating conditions of which they lack subjective understanding. Novel interventions are required to address these needs. - Virtual reality-based simulation training has been shown to be an effective training modality for healthcare professionals; it has the potential to deliver crucial empathy-building learning for frontline mental health staff due to its capacity to increase staff understanding of service users' experiences. Virtual reality and simulation technology take interactivity and experiential learning to a level beyond which we have seen in teaching and training before. Subjective understanding is elicited because this is a technology for enhanced experiential learning, which in turn fosters greater empathy and compassion. Increased empathy in the workforce is likely to yield significant benefits for service users. Greater empathy in nursing is linked with reduced restrictive practices and reduced conflict between staff and service users. Restrictive practices, including restraint and seclusion, are widely used in mental health settings within the UK, and are an aspect of mental health nursing that is at odds with the therapeutic role of nursing. - Despite these innovative developments, there are challenges ahead. Many nurses feel that complete eradication of restrictive practices is impossible and that barriers include a limitation of resources, communication, management, and lack of education. There is a need to make simulation training economically viable so that it can be upscaled and widely available. Therefore, greater investment and resources are needed to bring this innovative training to the wider workforce to support staff and to realise the benefits for service users. | United Kingdom |
| **Riva et al., 2016** | Riva, G., Villani, D., Cipresso, P., Repetto, C., Triberti, S., Di Lernia, D., et al.  (2016). Positive and transformative technologies for active ageing. Stud. Health  Technol. Inform. 220, 308–315. | IV- Commentary | Discusses the possible role of positive and transformative technologies for healthy living and active ageing by presenting different practical applications of this approach recently developed by this team | - Due to advances in treatment and people's living longer, chronic diseases are becoming more common among our population. This is a leading contributor to the increasing burden on our current healthcare system. To reduce this burden and sufficiently meet the needs of this growing segment of the population, healthcare organisations must encourage the elderly to take a more active role in caring for their own health and well-being. - "Positive Technology" focuses on the use of technology for improving the quality of our personal experience, and it suggests specific strategies for modifying/improving each of the different dimensions involved - Emotional Quality (affect regulation); Engagement/Actualization (presence and flow); Connectedness (collective intentions and networked flow) - and for generating motivation and engagement in the process. - "Transformative Technology" are technologically-mediated experiences that support positive, enduring transformation of the self-world. The transformative content is delivered through a set of experiential affordances, which are stimuli designed to elicit emotional and cognitive involvement in the designed experience: (i) emotional affordances; (ii) epistemic affordances. | Italy |
| **Rodgers et al., 2018** | Rodgers, R. F., Donovan, E., Cousineau, T., Yates, K., McGowan, K., Cook, E.,  et al. (2018). BodiMojo: efficacy of a mobile-based intervention in improving body  image and self-compassion among adolescents. J. Youth Adolesc. 47, 1363–1372.  doi: 10.1007/s10964-017-0804-3 | II- Research study | Randomized controlled evaluation of BodiMojo, a mobile application (app) intervention grounded in self-compassion to promote positive body image. | - A sample of 274 adolescents, mean (SD) age = 18.36 (1.34) years, 74% female, were allocated to a control group or used BodiMojo for 6 weeks. Appearance esteem, body image flexibility, appearance comparison, mood, and self-compassion were assessed at baseline, 6, and 12 weeks. - Significant time by group interactions emerged for appearance esteem and self-compassion, with appearance esteem and self-compassion increasing in the intervention relative to the control group. These findings provide preliminary support for BodiMojo, a cost-effective mobile app for positive body image. | United States |
| **Rossi et al., 2020** | Rossi, S., Conti, D., Garramone, F., Santangelo, G., Staffa, M., Varrasi, S.,  et al. (2020). The role of personality factors and empathy in the acceptance and  performance of a social robot for psychometric evaluations. Robotics 9:39. | VI- Design study | Robot-led cognitive tests in socially assistive robotics | - Research and development in socially assistive robotics have produced several novel applications in the care of senior people. However, some are still unexplored such as their use as psychometric tools allowing for a quick and dependable evaluation of human users' intellectual capacity. To fully exploit the application of a social robot as a psychometric tool, it is necessary to account for the users' factors that might influence the interaction with a robot and the evaluation of user cognitive performance. - Senior participants were invited to use a prototype of a robot-led cognitive test. Analysed the influence of personality traits and user's empathy on the cognitive performance and technology acceptance. - Results show a positive influence of a personality trait, the "openness to experience", on the human-robot interaction, and that other factors, such as anxiety, trust, and intention to use, are influencing technology acceptance and correlate the evaluation by psychometric tests. | Italy |
| **Roswell et al., 2020** | Roswell, R. O., Cogburn, C. D., Tocco, J., Martinez, J., Bangeranye, C.,  Bailenson, J. N., et al. (2020). Cultivating empathy through virtual reality:  advancing conversations about racism, inequity, and climate in medicine. Acad.  Med. 95, 1882–1886. doi: 10.1097/ACM.0000000000003615 | VI- Design study | Cultivating empathy through virtual reality by advancing conversations about racism, inequity, and climate in medicine | - Racism and bias are fundamental causes of health inequities, and they negatively affect the climate of academic medical institutions across the United States. In 2019, the Zucker School of Medicine and Northwell Health piloted a virtual reality (VR) racism experience as a component of professional development for medical school and health system leaders, faculty, and staff. Participants experienced a 60-minute, interactive, large-group session on microaggressions and, as individuals, a 20-minute VR module. These were followed by group reflection and debriefing. The sessions, developed in collaboration with a VR academic team, represented a response to institutional climate assessment surveys, which indicated the need for expanded professional training on cross-cultural communication and enhancing inclusion. - Most staff participants (n=112) (90.8%) reported feeling engaged in the VR experience and agreed that VR was an effective tool for enhancing empathy (94.7%), that the session enhanced their own empathy for racial minorities (85.5%), and that their approach to communication would change (67.1%). In open-ended responses, participants frequently conveyed enthusiasm, powerful emotional and physiologic responses, and enhanced empathy. They also suggested more time for follow-up discussions. | United States |
| **Saab et al. 2022** | Saab, M. M., Landers, M., Murphy, D., O’Mahony, B., Cooke, E., O’Driscoll,  M., et al. (2022). Nursing students’ views of using virtual reality in healthcare: a  qualitative study. J. Clin. Nurs. 31, 1228–1242. doi: 10.1111/jocn.15978 | II- Research study | This qualitative study explored nursing students' views of using virtual reality in healthcare | - Nursing students (n = 26) were recruited using convenience and snowball sampling. They were first exposed to a virtual reality intervention aimed to enhance men's awareness of testicular diseases. This was attempted to familiarise participants with the technology and initiate conversations around its use in healthcare. Participants were then interviewed face-to-face, either individually or within focus groups. - Four themes were identified: (i) positive experiences of virtual reality; (ii) challenges to using virtual reality; (iii) settings where virtual reality can be implemented; and (iv) blue-sky and future applications of virtual reality. - Participants described this technology as novel, enjoyable, immersive, memorable and inclusive. They questioned, however, the suitability of virtual reality for older adults, reported minor technical difficulties and stressed the importance of prior preparation in the use of the technology. Virtual reality was recommended for use in outpatient healthcare settings, schools and the community. Participants suggested using virtual reality in health promotion, disease prevention and management, and to promote nurses' empathy towards patients. | Republic of Ireland |
| **Sabo et al., 2022** | Sabo, A., Mehdizadeh, S., Iaboni, A., and Taati, B. (2022). Estimating  Parkinsonism severity in natural gait videos of older adults with dementia. IEEE J.  Biomed. Health Inform. 26, 2288–2298. doi: 10.1109/JBHI.2022.3144917 | VI- Design study | Vision-based human pose- estimation | - Drug-induced parkinsonism affects many older adults with dementia, often causing gait disturbances. New advances in vision-based human pose- estimation have opened possibilities for frequent and unobtrusive analysis of gait in long-term care settings. This work leverages spatial-temporal graph convolutional network (ST-GCN) architectures and training procedures to predict clinical scores of parkinsonism in gait from video of individuals with dementia. Proposes a two-stage training approach consisting of a self-supervised pretraining stage that encourages the ST-GCN model to learn about gait patterns before predicting clinical scores in the finetuning stage. The proposed ST-GCN models are evaluated on joint trajectories extracted from video and are compared against traditional (ordinal, linear, random forest) regression models and temporal convolutional network baselines. Three 2D human pose-estimation libraries (OpenPose, Detectron, AlphaPose) and the Microsoft Kinect (2D and 3D) are used to extract joint trajectories of 4787 natural walking bouts from 53 older adults with dementia. A subset of 399 walks from 14 participants is annotated with scores of parkinsonism severity on the gait criteria of the Unified Parkinson's Disease Rating Scale (UPDRS) and the Simpson-Angus Scale (SAS). - Results demonstrate that ST-GCN models operating on 3D joint trajectories extracted from the Kinect consistently outperform all other models and feature sets. Prediction of parkinsonism scores in natural walking bouts of unseen participants remains a challenging task, with the best models achieving macro-averaged F1-scores of 0.53 +/- 0.03 and 0.40 +/- 0.02 for UPDRS-gait and SAS-gait, respectively. | Canada |
| **Sanal et al., 2019** | Sanal, M. G., Paul, K., Kumar, S., and Ganguly, N. K. (2019). Artificial  intelligence and deep learning: the future of medicine and medical practice.  J. Assoc. Phys. India 67, 71–73. | IV- Commentary | Artificial intelligence and deep learning and the implications for the future of medicine and medical practice | - Discusses how artificial Intelligence (AI) and access to "Big Data" together with the evolving techniques in biotechnology will change the medical practice. Many diseases such as type II diabetes will no longer be considered as a single disease. Many familiar cancers such as cancer of liver or pancreas will have hundreds of subtypes whose management will be very different. The way we think about diseases will change. It will no longer be possible for clinicians to make a diagnosis, remember the names of diseases, the names of drugs or management protocols without the help of computers. As computer intelligence becomes more important than human intelligence in deciding diagnosis and treatment there will be a paradigm in the role of doctors. Internet, computers and social media will become more important than individuals in decision making. As a result, medicine will go more and more egalitarian ("wiki") with increasing community participation in health decision making and management. A socialistic pattern will evolve over time globally as an adaptive reaction to the pressures put by artificial intelligence. This is because the individual differences in knowledge or intellect between human beings will become less apparent compared to the superpowers of artificial intelligence. Qualities which are unique for humans such as compassion, empathy and emotional care will decide the professional success of future physicians even more than today. Today we are using artificial intelligence in diagnosis and prediction to help clinicians. Clinical algorithms and human experience cannot be replaced by machines. It will take many years to completely merge or replace humans with machines. However, we need to modify our medical education system in order to prepare the medical community and sensitize the society well in advance for a smooth transition. | India |
| **Sanders et al., 2021** | Sanders, J. J., Caponigro, E., Ericson, J. D., Dubey, M., Duane, J. N., Orr,  S. P., et al. (2021). Virtual environments to study emotional responses to clinical communication: a scoping review. Patient. Educ. Couns. 104, 2922–2935. doi:  10.1016/j.pec.2021.04.022 | III- Scoping review | Explores the potential for virtual environments (VE) to evaluate emotional outcomes in clinical communication research | - Twenty-one articles met inclusion criteria. They applied different methodological approaches, including a range of VE technologies and diverse emotional outcome measures, such as psychophysiological arousal, emotional valence, or empathy. Major research topics included use of virtual reality to provoke and measure emotional responses, train clinicians in communication skills, and increase clinician empathy. - Researchers may leverage VE technologies to ethically and systematically examine how characteristics of clinical interactions, environments, and communication impact emotional reactions and responses among patients and clinicians. Variability exists in how VE technologies are employed and reported in published literature, and this may limit the internal and external validity of the research. However, virtual reality can provide a low-cost, low-risk, experimentally controlled, and ecologically valid approach for studying clinician-patient communication. - Future research should leverage psychophysiological measures to further examine emotional responses during clinical communication scenarios and clearly report virtual environment characteristics to support evaluation of study conclusions, study replicability, and meta-analyses. | United States |
| **Sarkar et al., 2020** | Sarkar, P. P., Tohin, M. A., Khaled, M. A., and Rahman, M. S. (2020).  “Implementation of an instrumented crutch with scalable E-care architecture  using IoT,” in Proceeding of the 2020 IEEE region 10 symposium, 242–245. | VI- Design study | Study of the implementation of an instrumented crutch with scalable e-care architecture using IoT | - The aim of this research is to convert this project into product by which a paralyzed patient can get the comfort feeling while using it and get into this applicable smart feature of this crutch with its scalable e-care architecture. The motive of this work is to support a paralyzed patient to rise up from a wheelchair and give them the opportunity to move without any other help. Many of paralyzed people have a common problem of falling. The aim is to reduce the users falling tendencies with the help of Ultrasonic sensor. The objective of this research work is to help the patient to move freely, actual health monitoring with real time tracking and give them the effective multifunctional smart applications by which they can feel confident while using the crutch. Digital upgrade of manual crutch with its robotics and IoT features. This crutch as a humanitarian project is designed to explore empathy for paralyzed patients in an effective system. | Bangladesh |
| **Sass, 2014** | Sass, H. (2014). Integrate bioethics in the new epoch. Synthe. Philos. 29, 415–427. | IV- Perspective | Bioethical perspective on advanced technologies and the value of bio-ethics for integrating nature, technology and culture and cultivating a harmonious bio | - The new epoch of the 21th century develops new globally interrelated and interacting high-tech and cyberspace based civilizations and rapid transitions and other interactions between old and new models of life, orientation and behaviour. Biology and sociology describe bios as interrelated interactions of living beings and biotopes, natural, cultural, technical. The concept of bio-ethics in the New Epoch has to be inclusive in integrating natures, technologies, and cultures. The potential for catastrophe or cultivation depends on strengthening and integrating the six basic bioethical human properties, attitudes, and virtues - communication and cooperation, competence and compassion, competition and cultivation. These 6 C-principles have empirically and historically been successful in cultivating harmonious bios, i.e. living-together in interrelation environmentally, economically, politically and culturally. Serve as essential preconditions and components for the successful and comfortable survival of individuals, communities, cultural and natural environments and biotopes in the New Epoch. | United States |
| **Schick et al., 2021** | Schick, A., Paetzold, I., Rauschenberg, C., Hirjak, D., Banaschewski, T., Meyer-  Lindenberg, A., et al. (2021). Effects of a novel, transdiagnostic, hybrid ecological  momentary intervention for improving resilience in youth (emicompass):  protocol for an exploratory randomized controlled trial. JMIR Res. Proto.  10:e27462. doi: 10.2196/27462 | II- Research study | Study to investigate the clinical feasibility, candidate underlying mechanisms, and initial signals of the efficacy of a novel, transdiagnostic, hybrid EMI for improving resilience to stress in youth (EMIcompass) | - Explains that most mental disorders first emerge in youth and, in their early stages, surface as subthreshold expressions of symptoms comprising a transdiagnostic phenotype of psychosis, mania, depression, and anxiety. Elevated stress reactivity is one of the most widely studied mechanisms underlying psychotic and affective mental health problems. Thus, targeting stress reactivity in youth is a promising indicated and translational preventive strategy for adverse mental health outcomes that could develop later in life and for improving resilience. Compassion-focused interventions offer a wide range of innovative therapeutic techniques that are particularly amenable to being implemented as ecological momentary interventions (EMIs), a specific type of mobile health intervention, to enable youth to access interventions in a given moment and context in daily life. This approach may bridge the current gap in youth mental health care. - In an exploratory randomized controlled trial, youth aged between 14 and 25 years with current distress, a broad Clinical High At-Risk Mental State, or the first episode of a severe mental disorder will be randomly allocated to the EMIcompass intervention (ie, EMI plus face-to-face training sessions) in addition to treatment as usual or a control condition of treatment as usual only. Primary (stress reactivity) and secondary candidate mechanisms (resilience, interpersonal sensitivity, threat anticipation, negative affective appraisals, and momentary physiological markers of stress reactivity), as well as primary (psychological distress) and secondary outcomes (primary psychiatric symptoms and general psychopathology), will be assessed at baseline, postintervention, and at the 4-week follow-up. - This study is the first to examine feasibility, evidence on underlying mechanisms, and preliminary signals of the efficacy of a compassion-focused EMI in youth. If successful, a confirmatory randomized controlled trial will be warranted. The approach has the potential to significantly advance preventive interventions in youth mental health provision. | Germany |
| **Schmidt et al., 2022** | Schmidt, L. I., Schlomann, A., Gerhardy, T., and Wahl, H. (2022). “Aging  means to me. . . that i feel lonely more often”? An experimental study on the  effects of age simulation regarding views on aging. Front. Psychol. 13:806233.  doi: 10.3389/fpsyg.2022.806233. | II- Research study | Study to address possible effects of age simulation suits (ASS) interventions on multiple outcomes related to views on aging and age stereotypes | - Explains that over the last decades, educational programs involving age simulation suits (ASS) emerged with the ambition to further the understanding of age-related loss experiences, enhance empathy and reduce negative attitudes toward older adults in healthcare settings and in younger age groups at large. However, the impact of such "instant aging" interventions on individuals' personal views on aging have not been studied yet. - In a within-subjects design, N = 40 participants (M = 61.4 years, SD = 6.16) went through a series of established geriatric assessments (i.e., Timed up and Go) with and without an ASS. Views on aging constructs were assessed in standardized questionnaires before and after the ASS intervention. - Changes in aging-related cognitions were observed, with more negative expectations regarding social integration and continuous development after wearing the ASS. AARC and age stereotypes did not change from pre- to post-assessment, but participants reported an increased susceptibility to age-associated impairments and stronger feelings of obsolescence. Those participants who exhibited higher difficulties in geriatric assessments while wearing the suit reported higher openness to be supported by intelligent assistive devices or robots afterwards. - Concludes that ASS interventions should only be combined with education on losses and gains during the aging process to prevent negative effects on individual views on aging. On the other hand, potentials regarding technology acceptance and formation of intentions to engage in prevention and health behaviours among middle-aged to young-old adults are discussed. | Germany |
| **Scholten et al., 2017** | Scholten, M. R., Kelders, S. M., and Van Gemert-Pijnen, J. E. (2017). Selfguided  web-based interventions: scoping review on user needs and the potential  of embodied conversational agents to address them. J. Med. Int. Res. 19:e383.  doi: 10.2196/jmir.7351 | III- Scoping review | Explores what is known in literature about what support a user needs to stay motivated and engaged in an electronic health (eHealth) intervention that requires repeated use and the current potential of embodied conversational agents (ECAs) to provide this support | - Explains that web-based mental health interventions have evolved from innovative prototypes to evidence-based and clinically applied solutions for mental diseases such as depression and anxiety. Open-access, self-guided types of these solutions hold the promise of reaching and treating a large population at a reasonable cost. However, a considerable factor that currently hinders the effectiveness of these self-guided Web-based interventions is the high level of nonadherence. The absence of a human caregiver apparently has a negative effect on user adherence. It is unknown to what extent this human support can be handed over to the technology of the intervention to mitigate this negative effect. - This study reviews and interprets the available literature on (1) support within eHealth interventions that require repeated use and (2) the potential of ECAs by means of a scoping review. The rationale for choosing a scoping review is that the subject is broad, diverse, and largely unexplored. Themes for (1) and (2) were proposed based on grounded theory and mapped on each other to find relationships. - Results suggest the presence of user needs that largely remain implicit and unaddressed. These support needs can be categorized as task-related support and emotion-related support. The results of the second part of this study suggest that ECAs are capable of engaging and motivating users of information technology applications in the domains of learning and behavioural change. - Longitudinal studies must be conducted to determine under what circumstances ECAs can create and maintain a productive user relationship. Mapping the user needs on the ECAs' capabilities suggests that different kinds of ECAs may provide different solutions for improving the adherence levels. - Autonomous ECAs that do not respond to a user's expressed emotion in real time but take on empathic roles may be sufficient to motivate users to some extent. It is unclear whether those types of ECAs are competent enough and create sufficient believability among users to address the user's deeper needs for support and empathy. Responsive ECAs may offer a better solution. However, at present, most of these ECAs have difficulties to assess a user's emotional state in real time during an open dialogue. By conducting future research with relationship theory-based ECAs, the added value of ECAs toward user needs can be better understood. | Netherlands |
| **Shepherd and Majchrzak, 2022** | Shepherd, D., and Majchrzak, A. (2022). Machines augmenting entrepreneurs:  opportunities (and threats) at the nexus of artificial intelligence and  entrepreneurship. J. Bus. Vent. 37, 883–9026. doi: 10.1016/j.jbusvent.2022.106227 | IV- Commentary | Opportunities and threats of artificial intelligence and entrepreneurship | - Discusses how artificial intelligence and entrepreneurialism are combined and used will determine their impact on humanity. To indicate the scope of current and future AI, this article provides examples of AI (at different levels of development) for four sectors-customer service, financial, healthcare, and tertiary education. Opportunities include (1) capitalize on the "feeling economy," (2) redistribute occupational skills in the economy, (3) develop and use new governance mechanisms, (4) keep humans in the loop (i.e., humans as part of the decision-making process), (5) expand the role of humans in developing AI systems, and (6) expand the purposes of AI as a tool. | United States |
| **Shorey, et al. 2019** | Shorey, S., Ang, E., Yap, J., Ng, E. D., Lau, S. T., and Chui, C. K. (2019). A  virtual counseling application using artificial intelligence for communication skills  training in nursing education: development study. J. Med. Int. Res. 21:e14658.  doi: 10.2196/14658 | VI- Design study | Study of the use of virtual patients (VPs) to better prepare nursing undergraduates for communicating with real-life patients, their family members, and other health care professionals during their clinical postings | - The stages of the creation of VPs included preparation, design, and development, followed by a testing phase before the official implementation. An initial voice chatbot was trained using a natural language processing engine, Google Cloud's Dialogflow, and was later visualized into a three-dimensional (3D) avatar form using Unity 3D. - The VPs included four case scenarios that were congruent with the nursing undergraduates' semesters' learning objectives: (1) assessing the pain experienced by a pregnant woman, (2) taking the history of a depressed patient, (3) escalating a bleeding episode of a postoperative patient to a physician, and (4) showing empathy to a stressed-out fellow final-year nursing student. Challenges arose in terms of content development, technological limitations, and expectations management, which can be resolved by contingency planning, open communication, constant program updates, refinement, and training. - The creation of VPs to assist in nursing students' communication skills training may provide authentic learning environments that enhance students' perceived self-efficacy and confidence in effective communication skills. However, the authors suggest that further refinement and constant enhancements are needed to train the VPs to simulate real-life conversations before the official implementation. | Singapore |
| **Sikstrom et al., 2022** | Sikstrom, L., Maslej, M. M., Hui, K., Findlay, Z., Buchman, D. Z., and Hill, S. L.  (2022). Conceptualising fairness: three pillars for medical algorithms and health  equity. BMJ Health Care Inform 29:e100459. doi: 10.1136/bmjhci-2021-100459 | III- Literature review | Environmental scan of literature on fairness | - Fairness is a core concept meant to grapple with different forms of discrimination and bias that emerge with advances in artificial intelligence. Yet, claims to fairness in ML discourses are often vague and contradictory with technocratic responses. Studies either measure (mathematically) competing definitions of fairness, and/or recommend a range of governance tools (eg, fairness checklists or guiding principles). - The review identified 'Three Pillars for Fairness': transparency, impartiality and inclusion. These insights are used to propose a multidimensional conceptual framework to guide empirical research on the operationalisation of fairness in healthcare. Identifies areas for further research that would bolster ongoing commitments to fairness and health equity in healthcare. | Canada |
| **Slater et al., 2019** | Slater, P., Hasson, F., Gillen, P., Gallen, A., and Parlour, R. (2019). Virtual  simulation training: imaged experience of dementia. Int. J. Older People Nurs.  14:e12243. doi: 10.1111/opn.12243 | II- Research study | Virtual simulation training for dementia awareness | - Study to explore the impact of an interactive training experience on moral, emotive, behavioural and cognitive elements of empathy. - Virtual Dementia Tour (VDT (R)) programme. Interviews were conducted over a two-month period, and qualitative thematic analysis was used to analyse the data. - The four components (moral, emotive, behavioural and cognitive) of empathy were reflected in the findings. Overall the interactive training programme was perceived as useful, and emotionally, it provided an opportunity to "imagine what it is to live with dementia," enabling a cognitive, moral and behavioural reflection to occur, enhancing the empathic state. - In this study, the VDT (R) provides a different way of learning, with participants reporting the emergence of an empathic response. Results suggest that the emotional response laid the foundations to the behavioural or cognitive (objective and subjective) reaction which was underpinned by a moral reaction. - Virtual reality programmes are one step in the process for healthcare professionals caring empathetically for people with dementia; however, further research is required. | United Kingdom |
| **Slomian et al., 2017** | Slomian, J., Emonts, P., Vigneron, L., Acconcia, A., Reginster, J. Y., Oumourgh,  M., et al. (2017). Meeting the needs of mothers during the postpartum period:  using co-creation workshops to find technological solutions. JMIR Res. Protoc.  6:e76. doi: 10.2196/resprot.6831 | VI- Design study | Explains the use of co-creation workshops to find technological solutions to meet the needs of mothers during the postpartum period | - Parents and health professionals want solutions that include empathy (ie, to help fight against the feelings of abnormality and loneliness), that help mothers in daily life, that are personalized and adapted to different situations, that are educational, and that assures some continuity in their contact with health professionals. In practice, we found that parents and professionals think the solution should be accessible to everyone and available at all times. To address these criteria, technology experts proposed different solutions, such as a forum dedicated to the postpartum period that is supervised by professionals, a centralized website, a system of videoconferencing, an online exchange group, a "gift voucher" system, a virtual reality app, or a companion robot. - Suggests that the human component seems to be very important during the postnatal period. Nevertheless, technology could be a great ally in helping mothers during the postpartum period. Technology can help reliably inform parents and may also give them the right tools to find supportive people. Emphasises these new technologies should be tested in clinical trials. | Belgium |
| **Srivastava et al., 2020** | Srivastava, T. K., and Waghmare, L. S. (2020). Implications of artificial  intelligence (AI) on dynamics of medical education and care: a perspective. J. Clin.  Diagn. Res. 2020:2249. | IV- Perspective piece | Implications of artificial intelligence on dynamics of medical education and care | - Argues that AI is likely to profoundly impact systems of health care. Large data storing, processing and its interpretation through Electronic Medical records (EMRs) indicate great potential benefit to health services. New roles of a physician and health professionals should realise the importance of being efficient in interacting with AI technologies. Patient psychology and empathy should take a central place in patient care and doctors in training should be equipped with such relevant competencies. - Accordingly, Al needs to find a suitable place within the curriculum of medical education that deals with technology, interactive learning environments and managing Al systems. It is also imperative that medical teachers realise the potential of Al on health care and are suitably equipped to train these emerging concepts to future doctors. | India |
| **Stargatt et al., 2021** | Stargatt, J., Bhar, S., Petrovich, T., Bhowmik, J., Sykes, D., and Burns, K. (2021).  The effects of virtual reality-based education on empathy and understanding of the physical environment for dementia care workers in Australia: a controlled study.  J. Alzheimers Dis. 84, 1247–1257. doi: 10.3233/JAD-210723 | VI- Design study in educational context | The effects of virtual reality-based education on empathy and understanding of the physical environment for dementia care workers | - Dementia care workers enrolled in workshops on dementia care principles. Once participants were enrolled, workshops were assigned at random to deliver non-VR or VR-based education. Participants (N= 114, 91.8% female, mean age = 46.4; SD = 13.2; n= 60VR condition, 54control condition) completed self-report measures of empathy towards people living with dementia, understanding of dementia care environments, dementia knowledge, and attitudes towards dementia at pre- and post-workshop. - Significant pre-post main effects were observed for empathy, understanding of dementia care environments, and attitudes. Interaction effects were not found; improvements in outcomes were similar between conditions. However, interaction effects were observed for subgroups. Empathy improved significantly more in the VR condition for older participants. Understanding of dementia care environments improved more in the VR condition for younger and non-English-speaking background participants. - Using VR may not augment teaching outcomes for all learners. VR may differentially assist leaners of different ages and English-speaking backgrounds. More research is needed to understand for which variables and for whom VR is a useful teaching tool. | Australia |
| **Stein and Brooks, 2017** | Stein, N., and Brooks, K. (2017). A fully automated conversational artificial  intelligence for weight loss: longitudinal observational study among overweight  and obese adults. JMIR Diab. 2:e28. doi: 10.2196/diabetes.8590 | VI- Design study | Study to evaluate weight loss, changes in meal quality, and app acceptability among users of the Lark Weight Loss Health Coach AI (HCAI) with the overarching goal of increasing access to compassionate health care via mobile health. | - Explains that type 2 diabetes is the most expensive chronic disease in the United States. Two-thirds of US adults have prediabetes or are overweight and at risk for type 2 diabetes. Intensive in-person behavioural counselling can help patients lose weight and make healthy behaviour changes to improve their health outcomes. However, with the shortage of health care providers and associated costs, such programs do not adequately service all patients who could benefit. The health care system needs effective and cost-effective interventions that can lead to positive health outcomes as scale. - Study to evaluate weight loss, changes in meal quality, and app acceptability among 70 users of the Lark Weight Loss Health Coach AI (HCAI), with the overarching goal of increasing access to compassionate health care via mobile health. Lessons learned in this study can be applied when planning future clinical trials to evaluate HCAI and when designing AI to promote weight loss, healthy behaviour change, and prevention and self-management of chronic diseases. - Data were analysed for participants who met engagement standards set forth by the Centers for Disease Control and Prevention criteria for Diabetes Prevention Program, a clinically proven weight loss program focused on preventing diabetes. Weight loss (standard error of the mean) was 2.38% (0.69%) of baseline weight. The average duration of app use was 15 (SD 1.0) weeks, and users averaged 103 sessions each. Predictors of weight loss included duration of AI use, number of counselling sessions, and number of meals logged. Percentage of healthy meals increased by 31%. The in-app user trust survey had a 100% response rate and positive results, with a satisfaction score of 87 out of 100 and net promoter score of 47. - Concludes that use of an AI health coach is associated with weight loss comparable to in-person lifestyle interventions. It can also encourage behaviour changes and have high user acceptability. Research into AI and its application in telemedicine should be pursued, with clinical trials investigating effects on weight, health behaviours, and user engagement and acceptability. | United States |
| **Stenberg et al., 2015** | Stenberg, J. H., Joutsenniemi, K., and Holi, M. (2015). Nettiterapiat -  mitä tiedetään toimivuudesta [online therapies - what is known about their  functionality]. Duodecim Laaketieteellinen Aikakauskirja 131, 1297–1301. | IV- Perspective | Online psychotherapeutic therapies | - Online therapies are partly automated therapies, in which psychotherapeutic contents have been complemented with computer-aided presentational and educational contents, with a therapist giving support to the progress of the patient. As methods, these therapeutic programs incorporate therapeutic methods that have proven effective, such as remodeling of thoughts, activation of behaviour and exposure, empathy, strengthening of cooperative relationship and motivation, and general support for self-reflection. For example, online therapies already constitute part of the Finnish treatment guidelines on depression. Online therapies are available throughout Finland for the essential psychiatric illnesses. | Finland |
| **Sterkenburg and Vacaru, 2018** | Sterkenburg, P. S., and Vacaru, V. S. (2018). The effectiveness of a serious  game to enhance empathy for care workers for people with disabilities: a parallel  randomized controlled trial. Disabil Health J. 11, 576–582. doi: 10.1016/j.dhjo.  2018.03.003 | II- Research study | Study to investigate the effectiveness of the serious game "The world of EMPA" in enhancing empathy in care workers for people with disabilities, and test the effect of personal distress on empathy change post intervention | - Main results showed that the serious game did not significantly enhance empathy in care workers, whereas reading a digital information package yield a significant decrease in empathy. Exploratory analysis showed that the serious game decreased significantly personal distress in care-workers. This study showed that while the serious game "The world of EMPA" did not enhance empathy, it resulted in a decrease in personal distress in care workers for people with disabilities. | Netherlands |
| **Strudwick et al., 2020** | Strudwick, G., Impey, D., Torous, J., Krausz, R. M., and Wiljer, D. (2020).  Advancing E-mental health in canada: report from a multistakeholder meeting.  JMIR Ment. Health 7:e19360. doi: 10.2196/19360 | V- Conference | Electronic mental health (e-mental health) technologies | - E-mental health technologies may offer an important solution to the problem of unmet need for mental health service delivery. This topic was discussed in greater depth at the 9th Annual Canadian E-Mental Health Conference held in Toronto, Canada. - Themes that emerged from the discussions at the conference include (1) the importance of trust, transparency, human centeredness, and compassion in the development and delivery of digital mental health technologies; (2) an emphasis on equity, diversity, inclusion, and access when implementing e-mental health services; (3) the need to ensure that the mental health workforce is able to engage in a digital way of working; and (4) co-production of e-mental health services among a diverse stakeholder group becoming the standard way of working. | Canada |
| **Sukhera and Poleksic, 2021** | Sukhera, J., and Poleksic, J. (2021). Adapting compassion education through  technology-enhanced learning: an exploratory study. Acad. Med. 96, 1013–1020.  doi: 10.1097/ACM.0000000000003915 | II-Exploratory study | Adapting compassion education through technology-enhanced learning | - Participants (n=13) with experience of compassion education provided a range of responses regarding technology and compassion education. While participants revealed concerns about the constraints of technology on human interaction, they also described technology as both inevitable and necessary for the delivery of future compassionate care curricula. Participants also shared ways in which technology may enhance compassion education for health professionals by increasing accessibility and learner comfort with vulnerability. Addressing technological ambivalence, improving facilitation, and maintaining a balance between face-to-face instruction and technology-enhanced learning were identified as elements that could advance compassion education into the future. - Compassion education can be enhanced by technology; however, evidence-informed adaptation may require deliberate efforts to maintain some level of face-to-face interaction to ensure that technology does not erode human connection. - Further research is required to address the uncertainties surrounding technology and compassion education and to provide educators with guidance for adapting compassionate care curricula into a digital domain. | Canada |
| **Sung et al., 2022** | Sung, H. C., Su, H. F., Lee, W. L., Yamakawa, M., and Wang, H. M. (2022).  Effects of a dementia virtual reality-based training with peer support for home care  workers: a cluster randomized controlled trial. Int. J. Geriat. Psychiatry 37:5799.  doi: 10.1002/gps.5799 | II- Research study | Effects of a dementia virtual reality-based training with peer support for home care workers | - Home care workers who are the first-line care workers for community-dwelling dementia patients often have limited dementia knowledge, skills, and empathy towards those with dementia. Research is sparse on dementia care training using virtual reality (VR) technology and support network for home care workers. - This cluster randomized controlled trial evaluated the effects of a dementia VR-based training with peer support on dementia knowledge, attitude, competence, and empathy of home care workers. Each home care worker team was used as the unit for randomization. Sixteen teams were randomly assigned to either VR group or non-VR control group There was a total of 124 participants completed the study, the VR group (n = 61) received a dementia VR-based training consisted of 3-month dementia care e-book modules, dementia VR-based activity and 1-h monthly face-to-face peer support group meetings. The non-VR control group (n = 63) only receive the 3-month dementia care e-book modules and 1-h monthly regular staff meetings with no VR activity. Outcome measures were assessed at three time points: baseline, the end of the 3-month intervention, and 1-month post intervention. - Generalized estimating equations results indicate that the improvement in dementia knowledge, attitudes, competence, and empathy over time is significant in the VR group compared to the non-VR control group. The effects remained significant 1 month after the end of the 3-month intervention. - Innovative and accessible dementia training using VR technology with peer support is a promising training approach to improve dementia knowledge, attitudes, competence, and empathy of home care workers. | Taiwan |
| **Tanioka, 2019** | Tanioka, T. (2019). Nursing and rehabilitative care of the elderly using  humanoid robots. J. Med. Invest. JMI 66, 19–23. doi: 10.2152/jmi.66.19 | VI- Design study | Nursing and rehabilitative care of the elderly using humanoid robots | - Japan's declining birth rate and increasing aging population prompted intercessory efforts towards robot technologies in nursing practice for the elderly. Today, technological companies are developing robots that meet universal health care technology demands. While human caring focuses on human-to-human relationships, but between humans and nonhumans, e.g. Humanoid Nursing Robot (HNRs)-to-human relationships, caring practices have not been forthcoming. Suggests that when HNRs can support patients independently, capabilities much like being human will be required, including intelligence and skill competencies. - Currently, Tanioka's research group is conducting clinical trials of humanoid robots equipped with applications using Pepper (manufactured by SOFTBANK CORPORATION), towards elderly care and rehabilitation at the Mifune Hospital, Kagawa prefecture. Care Prevention Gymnastics Exercises (Pepper-CPGE) was madeby Xing Company, Japan. - Describes the clinical trial outcomes based on the Transactive Relationship Theory of Nursing (TRETON) (Tanioka, 2017) emphasizing nursing engagement processes between HNRs and human persons. Observable effects include positive changes in relationships of patients, humanoid robots and healthcare providers. Emphasizing ethical concerns and human person safety as critical factors of care, and fears for divergent robot use. | Japan |
| **Tanioka et al., 2019** | Tanioka, T., Yasuhara, Y., Dino, M., Kai, Y., Locsin, R. C., and Schoenhofer,  S. O. (2019). Disruptive engagements with technologies, robotics, and caring:  advancing the transactive relationship theory of nursing. Nurs. Administr. Quart.  43, 313–321. doi: 10.1097/NAQ.0000000000000365 | IV- Discussion | Disruptive engagements with technologies, robotics, and caring, advancing the Transactive Relationship Theory of Nursing | - Argues that human caring, founded on a Japanese caring perspective and nursing theory, fosters innovative and creative ideas for an aging society. The growing reality of health care dependency on technology presents a temptation to give robots utility as partners in nursing practice. Human caring expressed in human-to-human relationships, and also between humans and nonhumans, is a futuristic model for health care, with humanoid robots as major supporters. - Explores this disruptive technology, along with its functions and characteristics. Tanioka's Transactive Relationship Theory of Nursing is shared along with its relevance for addressing technological disruptions in health care. | Japan |
| **Tanioka et al., 2021** | Tanioka, T., Yokotani, T., Tanioka, R., Betriana, F., Matsumoto, K., Locsin,  R., et al. (2021). Development issues of healthcare robots: compassionate  communication for older adults with dementia. Int. J. Environ. Res. Public Health  18:4538. doi: 10.3390/ijerph18094538 | VI- Design study | Case study to explore the development issues of healthcare robots in expressing compassionate communication for older adults with dementia. | - Although progress is being made in affective computing, issues remain in enabling the effective expression of compassionate communication by healthcare robots. Identifying, describing and reconciling these concerns are important in order to provide quality contemporary healthcare for older adults with dementia. - An exploratory descriptive case study was conducted with the Pepper robot and older adults with dementia using high-tech digital cameras to document significant communication proceedings that occurred during the activities. The application program for an intentional conversation using Pepper was jointly developed by Tanioka's team and the Xing Company, allowing Pepper's words and head movements to be remotely controlled. - The analysis of the results revealed four development issues, namely, (1) accurate sensing behaviour for "listening" to voices appropriately and accurately interacting with subjects; (2) inefficiency in "listening" and "gaze" activities; (3) fidelity of behavioural responses; and (4) deficiency in natural language processing AI development, i.e., the ability to respond actively to situations that were not pre-programmed by the developer. - Conversational engagements between the Pepper robot and patients with dementia illustrated a practical usage of technologies with artificial intelligence and natural language processing. The development issues found in this study require reconciliation in order to enhance the potential for healthcare robot engagement in compassionate communication in the care of older adults with dementia. | Japan |
| **Terry and Cain, 2016** | Terry, C., and Cain, J. (2016). The emerging issue of digital empathy. Am. J.  Pharm. Educ. 80:58. doi: 10.5688/ajpe80458 | IV- Opinion piece | Digital empathy | - Empathy can have strong positive effects on patient outcomes, increase patient satisfaction, and reduce malpractice litigation. With modern advances in technology, however, the appropriate expression of empathy in today's age is being threatened, largely as a result of psychological processes that form online disinhibition. The digitization of health care and the corresponding decrease in the expression of empathy may be cause for concern. Because empathy is strongly correlated to positive health outcomes and is an important part of health professions in general, the construct of digital empathy should be considered for integration into health professions curricula. | United States |
| **Tiersen et al., 2021** | Tiersen, F., Batey, P., Harrison, M., Naar, L., Serban, A. I., Daniels, S., et al.  (2021). Smart home sensing and monitoring in households with dementia: user-centered  design approach. JMIR Aging 4:e27047. doi: 10.2196/27047. | II- Research study | Study to investigate the functional, psychosocial, and environmental needs of people living with dementia, their caregivers, clinicians, and health and social care service providers toward the design and implementation of smart home systems. | - This study used an iterative user-centered design approach comprising 9 sub studies. First, semi structured interviews (9 people with dementia, 9 caregivers, and 10 academic and clinical staff) and workshops (35 pairs of people with dementia and caregivers, and 12 health and social care clinicians) were conducted to define the needs of people with dementia, home caregivers, and professional stakeholders in both daily activities and technology-specific interactions. Then, the spectrum of needs identified was represented via patient-caregiver personas and discussed with stakeholders in a workshop (14 occupational therapists; 4 National Health Service pathway directors; and 6 researchers in occupational therapy, neuropsychiatry, and engineering) and 2 focus groups with managers of health care services (n=8), eliciting opportunities for innovative care technologies and public health strategies. Finally, these design opportunities were discussed in semi structured interviews with participants of a smart home trial involving environmental sensors, physiological measurement devices, smartwatches, and tablet-based chatbots and cognitive assessment puzzles (10 caregivers and 2 people with dementia). A thematic analysis revealed factors that motivate household members to use these technologies. - Participatory design methods supported the triangulation of stakeholder perspectives to aid the development of more patient-centered interventions and their translation to clinical practice and public health strategy. | United Kingdom |
| **Tong et al., 2022** | Tong, F., Lederman, R., D’Alfonso, S., Berry, K., and Bucci, S. (2022). Digital  therapeutic alliance with fully automated mental health smartphone apps: a  narrative review. Front. Psychiatry 13:819623. doi: 10.3389/fpsyt.2022.819623 | III- Literature review | Review to integrate the extant literature to identify research gaps and future directions in the investigation of digital therapeutic alliance (DTA) in relation to fully automated mental health smartphone apps. | - Fully automated mental health smartphone apps show strong promise in increasing access to psychological support. It is crucial to understand how to make these apps effective through digital therapeutic alliance (DTA) which needs to be conceptualized differently to traditional face-to-face therapeutic alliance (TA). - First, the role of bond in the context of fully automated apps is unclear. Second, human components of face-to-face TA, such as empathy, are hard to achieve in the digital context. Third, some users may perceive apps as more non-judgmental and flexible, which may further influence DTA formation. Subdisciplines of computer science, such as affective computing and positive computing, and some human-computer interaction (HCI) theories, such as those of persuasive technology and human-app attachment, can potentially help to foster a sense of empathy, build tasks and goals and develop bond or an attachment between users and apps, which may further contribute to DTA formation in fully automated smartphone apps. | Australia |
| **Torrence et al., 2022** | Torrence, C., Bhanu, A., Bertrand, J., Dye, C., Truong, K., and Madathil, K. C.  (2022). Preparing future health care workers for interactions with people with  dementia: a mixed methods study. Gerontol. Geriatr Educ. 2022, 1–20. doi: 10.  1080/02701960.2022.2042805 | II- Research study of educational intervention | Dementia sensitivity training using virtual reality: 41 university students were randomized into one of three conditions. All participants completed pre- and post-assessments and were interviewed. | - This mixed-methods study assessed the effectiveness of virtual reality as a delivery format for dementia tours compared to dementia tours that physically alter sensations. It also compared the effectiveness of deficit-focused dementia sensitivity training to reading strengths-focused case studies, a traditional instruction method. - Quantitative results indicate that a dementia tour offered through virtual reality is as effective as a physical-based tour; however, compared to reading case studies, participants reported poorer attitudes about living with AD and feeling less prepared for caregiving. The qualitative results show an increase in empathy across all conditions. Integration of findings indicates that dementia tours in both formats are effective at encouraging empathy and that both strengths-based and deficit-based sensitivity training are important components of education for future health care workers. | United States |
| **Trzeciak et al., 2017** | Trzeciak, S., Roberts, B. W., and Mazzarelli, A. J. (2017). Compassionomics:  hypothesis and experimental approach. Med. Hypoth. 107, 92–97. doi: 10.1016/  j.mehy.2017.08.015 | IV- Discussion | Compassionomics is the branch of knowledge and scientific study of the effects of compassionate healthcare | - Shows that recent reports indicate that healthcare is experiencing a compassion crisis - an absence of (or inconsistency in) compassionate patient care. It is currently unclear if, or to what extent, this exerts significant effects on health and healthcare. Experimental data are few, and this represents a critical knowledge gap for all health sciences. - Hypothesizes that compassionate care is beneficial for patients (better outcomes), healthcare systems and payers (lower costs), and healthcare providers (lower burnout). Compassionomics is the branch of knowledge and scientific study of the effects of compassionate healthcare. Describes a framework for hypothesis testing. If the hypotheses are confirmed, compassionate healthcare can be established in the domain of evidence-based medicine. | United States |
| **Valtolina and Hu, 2021** | Valtolina, S., and Hu, L. (2021). “Charlie: a chatbot to improve the elderly  quality of life and to make them more active to fight their sense of loneliness,”  in Proceeding of the CHItaly 2021: 14th biannual conference of the italian SIGCHI  chapter, doi: 10.1145/3464385.3464726 | VI- Design study | Design and testing of a chatbot to support loneliness and mental health in older people at home | - The development of a conversation agent in the healthcare domain presents several technical, design and linguistic challenges. Describes a chatbot conversing with elderly people, with age-related problems. - A chatbot, named Charlie, able to remember commitments and medicines, connect remotely with doctors, family, entertain and assist elders. The idea is to investigate solutions to increase their quality of life by providing companionship through innovative strategies based on gamification, active notifications, and promotion of self-compassion that can be explored for preventive mental healthcare. The hope of helping the elderly is more needed than ever today due to the Covid-19 pandemic since lockdowns and isolation have disrupted social lives, affecting their mental health due to loneliness. Also describes a preliminary evaluation of Charlie's personality and its level of acceptability by older people. | Italy |
| **van der Lubbe et al., 2022** | van der Lubbe, L. M., Groot, N., and Gerritsen, C. (2022). “Using topic  modelling to personalise a digital self-compassion training,” in Pervasive  computing technologies for healthcare - 15th eai international conference, pervasive  health 2021, proceedings, eds H. Lewy and R. Barkan. | V- Conference | Personalisation of self-compassion training for young people with mental health issues | - Young adults that struggle with mental health issues experience barriers to seek help. Online self-compassion training aims to overcome some of these barriers. To improve the training, personalised exercises can be devised based on topic modelling. Data from a pilot study is used to analyse and evaluate the algorithm. - Overall, the algorithm has an accuracy of 54.1% for predicting the right topic. This accuracy increases to 80.4% when considering an empty prediction to be correct as well. Although this research also shows that data makes the task of topic modelling difficult, it does prove to be a possibility to personalise the self-compassion training. | Netherlands |
| **van Pelt et al., 2022** | van Pelt, B., Nijman, S., van Haren, N., Veling, W., Pijnenborg, G., van Balkom,  I., et al. (2022). Dynamic interactive social cognition training in virtual reality  (DiSCoVR) for adults with autism spectrum disorder: a feasibility study. Res.  Autism Spectr. Dis. 96:102003. doi: 10.1016/j.rasd.2022.102003 | II- Feasibility study | Evaluates the feasibility and acceptance by participants and therapists of the virtual reality Dynamic Interactive Social Cognition training for autism spectrum disorder | - Social cognitive difficulties in Autism Spectrum Disorder (ASD) can affect the daily lives of people with ASD profoundly, impacting the development and maintenance of meaningful social relations. Social cognition training (SCT) is commonly used for improving social functioning but lacks ecological validity and the ability to effectively mimic social situations. - Development of virtual reality (VR) interventions, focusing on enhancing social cognition, could add to the effectiveness of SCT within ASD care, by offering a safe, interactive and practical training setting, where generalization of knowledge and skills to the real-world are promoted. - Training in Virtual Reality (DiSCoVR) protocol as developed for adults with schizophrenic spectrum disorder (SSD), adapted for ASD (DiSCoVR-A). 26 participants, aged 18-63, took part in a pilot study. 22 participants completed baseline and post-assessment, including primary outcome evaluation assessment through a semi-structured interview. Secondary measures focused on social cognition, emotion recognition, mental flexibility, social anxiety, empathy and social responsiveness and were assessed at baseline (T0), post-treatment (T1), and at follow-up (T2) sixteen weeks after completion of the intervention. Results: Our results show that the majority of participant and therapists found the VR intervention acceptable and feasible, as reported in evaluation questionnaires and interviews. These preliminary findings are promising; however, controlled research is needed to further investigate the effectiveness of VR within social cognition training for adults with ASD. | Netherlands |
| **van Rijn et al., 2017** | van Rijn, B., Cooper, M., Jackson, A. P., and Wild, C. (2017). Avatar-based  therapy within prison settings: pilot evaluation. Br. J. Guid. Couns. 45, 268–283. | VI- Design study | Avatar-based virtual reality therapy for mental health | - Presents an introduction of a newly developed, avatar-based virtual reality therapy, as an addition to the therapeutic programme, within a therapeutic community prison in the UK. The participants had six group sessions facilitated by a counsellor. The aim of the project was to investigate whether this approach would improve mental health outcomes for the prisoners, interpersonal relationships within the prison and facilitate the achievement of personal goals for the prisoners. The sample size (n=4) was insufficient to make firm conclusions about the mental health outcomes. However, the qualitative analysis showed a strong engagement with the programme in addressing personal issues, the development of insight and empathy, and improvements in relationships within the participants and with the counsellor. Further research with a larger sample is needed to establish efficacy of this type of therapy with the prison population. | United Kingdom |
| **Verma et al., 2021** | Verma, A. A., Murray, J., Greiner, R., Cohen, J. P., Shojania, K. G., Ghassemi,  M., et al. (2021). Implementing machine learning in medicine. CMAJ Can. Med.  Assoc. J. Assoc. Med. Can. 193, E1351–E1357. doi: 10.1503/cmaj.202434 | IV- Discussion | Proposes a 3-phase framework to develop and implement machine-learned solutions in clinical care | - Machine learning has the potential to transform health care, although its current application to routine clinical practice has been limited. Multidisciplinary partnership between technical experts and end-users, including clinicians, administrators, and patients and their families, is essential to developing and implementing machine-learned solutions in health care. - A 3-phase framework can be used to describe the development and adoption of machine-learned solutions: an exploration phase to understand the problem being addressed and the deployment environment, a solution design phase for the development of machine-learned models and user-friendly tools, and an implementation and evaluation phase to deploy and assess the impact of the machine-learned solution. | Canada |
| **Visram et al., 2022** | Visram, S., Leyden, D., Annesley, O., Bappa, D., and Sebire, N. J. (2022).  Engaging children and young people on the potential role of artificial intelligence  in medicine. Pediatr. Res. [Epub ahead of print]. doi: 10.1038/s41390-022-02053-4 | V- Workshop | Engaging children and young people on the potential role of artificial intelligence in medicine | - Workshop to investigate attitudes towards AI and its future applications in medicine and healthcare at a specialised paediatric hospital using practical design scenarios. Twenty-one members of a Young Persons Advisory Group for research contributed to an engagement workshop to ascertain potential opportunities, apprehensions, and priorities. - When presented as a selection of practical design scenarios, CYP were more open to some applications of AI in healthcare than others. Human-centeredness, governance and trust emerged as early themes, with empathy and safety considered as important when introducing AI to healthcare. Educational workshops with practical examples using AI to help, but not replace humans were suggested to address issues, build trust, and effectively communicate about AI. - Whilst policy guidelines acknowledge the need to include children and young people to develop AI, this requires an enabling environment for human-centred AI involving children and young people with lived experiences of healthcare. Future research should focus on building consensus on enablers for an intelligent healthcare system designed for the next generation, which fundamentally, allows co-creation. - A research gap on involving and engaging CYP in developing AI policies exists, there is little in the way of pragmatic and practical guidance for healthcare staff on this topic. This requires research on enabling environments for ongoing digital cooperation to identify and prioritise unmet needs in the application and development of AI. | United Kingdom |
| **Walker et al., 2020** | Walker, R. C., Tong, A., Howard, K., and Palmer, S. C. (2020). Clinicians’  experiences with remote patient monitoring in peritoneal dialysis: a semi-structured interview study. Peritoneal Dialy. Int. J. Int. Soc. Peritoneal Dialy. 40,  202–208. doi: 10.1177/0896860819887638 | II- Research study | How remote monitoring technologies might be used home care for peritoneal dialysis (kidney disease) | - Explains that fear of catastrophic events and uncertainty about safety at home are barriers to choosing peritoneal dialysis (PD). Remote monitoring may address these concerns and is increasingly being used in patients on automated peritoneal dialysis (APD). This study aims to describe clinicians' perspectives and experiences of remote monitoring in caring for patients on PD. Interviews with 12 nephrologists and 13 dialysis nurses across nine dialysis units in New Zealand who had experience using remote monitoring with patients on APD. - Four themes were identified: promoting and maintaining PD (providing reassurance to patients through continual surveillance, supporting confidence at home and sustaining PD as the patient-preferred treatment); enabling data-driven decisions (using comprehensive clinical data in providing timely and accessible care, and identifying and supporting patient adherence); establishing boundaries for use (negotiating privacy and independence, clarifying clinician and patient responsibilities and strengthening nursing innovation and capability); and enhancing patient-focused care (developing empathy for patients, enabling self-management and reducing time and financial burden in accessing care). - Concludes that remote monitoring is valued by clinicians in promoting and maintaining patients on PD and enabling data-driven decisions. Remote monitoring enhances patient-focused care, but clinicians also emphasise the need to protect patient privacy and establish boundaries for use. Remote monitoring that supports the clinicians' role and adheres to principles of data security maintains patient privacy may enhance care and outcomes for patients on PD. | New Zealand |
| **Wartman 2019** | Wartman, S. A. (2019). The empirical challenge of 21st-century medical  education. Acad. Med. J. Assoc. Am. Med. Coll. 94, 1412–1415. doi: 10.1097/ACM.  0000000000002866 | IV- Commentary | Curricula reform in medical education in relation to artificial intelligence and other changes | - Asserts that medical education is at a crossroads. Facing challenges wrought by science and technology as well as societal change, the curriculum is increasingly out of synch with new needs in teaching content and medical practice. The path to significant curricular reform is difficult because of a variety of factors, including deeply entrenched values, the natural resistance to change, and the accreditation process. Even the very definition of what it means to be a professional is changing with profound implications for the future role of the physician and the sacrosanct doctor-patient relationship. - The author enumerates challenges facing medical education in the current era. To address these challenges, the author recommends specific curricular emphases for 21st-century medical education: knowledge capture and curation, collaboration with and management of artificial intelligence applications, a deep understanding of probabilistic reasoning, and the cultivation of empathy and compassion in accordance with ethical standards. - Argues that given these needs, it is imperative that schools act today to undertake significant curricular reform and, in so doing, strive to make the hard changes necessary to produce optimal practitioners in a rapidly transforming 21st century. The author provides first steps an institution can take to begin to address these challenges. | United States |
| **Wartman and Combs, 2019** | Wartman, S. A., and Combs, C. D. (2019). Reimagining medical education in  the age of AI. AMA J. Ethics 21, E146–E152. doi: 10.1001/amajethics.2019.146 | IV- Commentary | Implications of artificial intelligence technologies for medical education | - Argues that available medical knowledge exceeds the organising capacity of the human mind, yet medical education remains based on information acquisition and application. Complicating this information overload crisis among learners is the fact that physicians' skill sets now must include collaborating with and managing artificial intelligence (AI) applications that aggregate big data, generate diagnostic and treatment recommendations, and assign confidence ratings to those recommendations. Thus, an overhaul of medical school curricula is due and should focus on knowledge management (rather than information acquisition), effective use of AI, improved communication, and empathy cultivation. | United States |
| **Weil-Dubuc, 2019** | Weil-Dubuc, P. L. (2019). Big data: amélioration technique, dégradation ou  transformation du modèle de solidarité ? [big data: technical improvement,  degradation or transformation of the solidarity model?]. Revue Epidemiol. Sante  Publi. 67, S19–S23. doi: 10.1016/j.respe.2018.12.060 | IV- Perspective | Explores perspectives on Big Data and health systems | - Argues that Big Data, the production of a massive amount of heterogeneous data, is often presented as a means to ensure the economic survival and sustainability of health systems. According to this perspective, Big Data could help save the spirit of welfare states based on the principles of risks-sharing and equal access to care for all. According to a second perspective, opposed to the first, Big Data would fuel a process of demutualization, transferring to individuals a growing share of responsibility for managing their health. This article proposes to develop a third approach: Big Data does not induce a loss of solidarity but a transformation of the European model of welfare states. These are the data that are now the objects of the pooling. Individual and collective responsibilities are thus redistributed. However, this model, as new as it is, remains liberal in its inspiration; it basically allows the continuation of political liberalism by other means. | France |
| **Wiljer et al., 2019** | Wiljer, D., Charow, R., Costin, H., Sequeira, L., Anderson, M., Strudwick, G.,  et al. (2019). Defining compassion in the digital health age: protocol for a scoping  review. BMJ Open. 9:e026338. doi: 10.1136/bmjopen-2018-026338 | III- Scoping review (protocol) | Defining compassion in a digital health age | - The notion of compassion and compassionate care is playing an increasingly important role in health professional education and in the delivery of high-quality healthcare. Digital contexts, however, are not considered in the conceptualisation of compassionate care, nor is there guidance on how compassionate care is to be exercised while using digital health technologies. The widespread diffusion of digital health technologies provides new contexts for compassionate care, with both opportunities for new forms and instantiations of compassion as well as new challenges. How compassion is both understood and enacted within this evolving, digital realm has not been synthesised. - This scoping review protocol explains the method to examine dimensions of compassionate professional practice when digital technologies are integrated into clinical care. - The results of the review will inform resource development and strategy for Associated Medical Services (AMS) Healthcare, a Canadian charitable organisation at the forefront of advancing research and leadership development in health and humanities, as part of the AMS Phoenix Project: A Call to Caring, particularly for digital professionalism frameworks so that they are inclusive of a compassion competency. | Canada |
| **Willems et al., 2021** | Willems, E., Vermeulen, J., van Haastregt, J., and Zijlstra, G. (2021). Technologies to improve the participation of stroke patients in their home  environment. Disabili. Rehabili. 2021, 1–11. doi: 10.1080/09638288.2021.1983041 | II- Qualitative research | Study to identify possible technological solutions that can contribute to stroke patients' participation at home | - Data on factors that negatively influenced participation at home were collected via semi-structured interviews with stroke patients (n = 6). Additionally, data on possible technologies to improve this participation were collected via a group interview with experts (n = 4). - Patients reported 21 factors negatively influencing participation at home, including psychological, cognitive, and physical factors. Experts suggested technological solutions regarding these factors to increase participation of stroke patients; digital assistants, apps, and virtual reality were frequently mentioned. To facilitate the use of these technologies, experts indicated the importance of involving patients in their design. They also suggested that rehabilitation specialists and family members could support the uptake and use of technologies. | Netherlands |
| **Wilson-Howard et al, 2021** | Wilson-Howard, D., Vilaro, M. J., Neil, J. M., Cooks, E. J., Griffin, L. N., Ashley,  T. T., et al. (2021). Development of a credible virtual clinician promoting colorectal  cancer screening via telehealth apps for and by black men: qualitative study. JMIR  Format. Res. 5:e28709. doi: 10.2196/28709 | VI- Design study | Study to incorporate the perceptions of Black men in the development of a virtual clinician (VC) designed to deliver precision messages promoting the fecal immunochemical test (FIT) kit for CRC screening among Black men in a future clinical trial | - Explains that traditionally, promotion of colorectal cancer (CRC) screening among Black men was delivered by community health workers, patient navigators, and decision aids (printed text or video media) at clinics and in the community setting. A novel approach to increase CRC screening of Black men includes developing and utilizing a patient-centered, tailored message delivered via virtual human technology in the privacy of one's home. - Focus groups of Black men were recruited to understand their perceptions of a Black male VC. Specifically, these men identified source characteristics that would enhance the credibility of the VC. The modality, agency, interactivity, and navigability (MAIN) model, which examines how interface features affect the user's psychology through four affordances (modality, agency, interactivity, and navigability), was used to assess the presumed credibility of the VC and likability of the app from the focus group transcripts. Each affordance triggers heuristic cues that stimulate a positive or a negative perception of trustworthiness, believability, and understandability, thereby increasing source credibility. - In total, 25 Black men were recruited from the community and contributed to the development of 3 iterations of a Black male VC over an 18-month time span. Feedback from the men enhanced the visual appearance of the VC, including its movement, clothing, facial expressions, and environmental surroundings. Heuristics, including social presence, novelty, and authority, were all recognized by the final version of the VC, and creditably was established. The VC was named Agent Leveraging Empathy for eXams (ALEX) and referred to as "brother-doctor," and participants stated, "wanting to interact with ALEX over their regular doctor." - Argues that involving Black men in the development of a digital health care intervention is critical. This population is burdened by cancer health disparities and incorporating their perceptions in telehealth interventions will create awareness of the need to develop targeted messages for Black men. | United States |
| **Wood et al, 2017** | Wood, A. E., Prins, A., Bush, N. E., Hsia, J. F., Bourn, L. E., Earley, M. D.,  et al. (2017). Reduction of burnout in mental health care providers using the  provider resilience mobile application. Commun. Ment Health J. 53, 452–459.  doi: 10.1007/s10597-016-0076-5 | VI- Design study | Pilot study of the usability, acceptability, and effectiveness of a free Provider Resilience (PR) mobile application (app) designed by the National Center for Telehealth and Technology to reduce provider burnout | - Outpatient mental health providers (N = 30) used the PR app for 1 month. Participants rated the PR app on the System Usability Scale with an overall score of 79.7, which is in the top quartile for usability. - Results of paired sample t tests on the Professional Quality of Life Scale indicated significant decreases on the Burnout (t = 3.65, p < .001) and Compassion Fatigue (t = 4.54, p < .001) subscales. The Provider Resilience app shows promise in reducing burnout and compassion fatigue in mental health care providers. | United States |
| **Wu et al., 2017** | Wu, K., Liu, C., Taylor, S., Atkins, P. W., and Calvo, R. A. (2017). “Automatic  mimicry detection in medical consultations,” in Proceeding of the 2017 IEEE Life  Sciences Conference (LSC), 55–58. | VI- Design study | Automatic mimicry detection in medical consultations | - Good communication and rapport between patients and doctors is important to achieve positive health outcomes. Mimicry, when a person copies the behaviour of someone else, has been related to good communication and building rapport. - Development of a method to automatically detect non-verbal mimicry and study the relationship between mimicry and the perceived quality of the interaction. Automating the detection of mimicry is important since it may be a proxy for empathy and perspective taking, both features of high-quality interactions for caring professions. This pilot study involved 91 sessions between medical students and volunteers acting as patients. The results confirm a correlation between our measure of mimicry and the assessed quality of the interaction. Therefore, medical students may improve their communication skills by practicing behavioural mimicry. | Australia |
| **Wu et al., 2019** | Wu, Y. J. A., Lan, Y. J., Huang, S. B. P., and Lin, Y. T. R. (2019). Enhancing  medical students’ communicative skills in a 3D virtual world. Educ. Technol. Soc.  22, 18–32. | II- Research study | Explores the effects of how collaborating in a virtual world (VW) enhanced learners' healthcare professional-patient communicative skills, including physician-patient and inter-professional communication in medical discourse. | - The results include (1) rich description of the scenarios and plots created by students in the VW group, (2) VW group learners' better performance in using effective communicative skills when role-playing via the VW, including building rapport with the patients and colleagues and showing empathy and understanding toward patients; and (3) VW group learners' higher evaluation of how the role-play helped their English language skills, healthcare professional-patient communication and learning in general. | Taiwan |
| **Xiao et al., 2015** | Xiao, B., Imel, Z. E., Georgiou, P. G., Atkins, D. C., and Narayanan, S. S.  (2015). "Rate my therapist": automated detection of empathy in drug and alcohol  counseling via speech and language processing. PLoS One 10:e0143055. doi: 10.  1371/journal.pone.0143055 | VI-Design study | "Rate My Therapist": Automated detection of empathy in drug and alcohol counselling via speech and language processing. | - The technology for evaluating patient-provider interactions in psychotherapy-observational coding-has not changed in 70 years. It is labour-intensive, error prone, and expensive, limiting its use in evaluating psychotherapy in the real world. Engineering solutions from speech and language processing provide new methods for the automatic evaluation of provider ratings from session recordings. The primary data are 200 Motivational Interviewing (MI) sessions from a study on MI training methods with observer ratings of counsellor empathy. - Automatic Speech Recognition (ASR) was used to transcribe sessions, and the resulting words were used in a text-based predictive model of empathy. Two supporting datasets trained the speech processing tasks including ASR (1200 transcripts from heterogeneous psychotherapy sessions and 153 transcripts and session recordings from 5 MI clinical trials). - The accuracy of computationally-derived empathy ratings was evaluated against human ratings for each provider. Computationally-derived empathy scores and classifications (high vs. low) were highly accurate against human-based codes and classifications, with a correlation of 0.65 and F-score (a weighted average of sensitivity and specificity) of 0.86, respectively. Empathy prediction using human transcription as input (as opposed to ASR) resulted in a slight increase in prediction accuracies, suggesting that the fully automatic system with ASR is relatively robust. Using speech and language processing methods, it is possible to generate accurate predictions of provider performance in psychotherapy from audio recordings alone. This technology can support large-scale evaluation of psychotherapy for dissemination and process studies. | United States |
| **Yaghy et al., 2019** | Yaghy, A., Shields, J. A., and Shields, C. L. (2019). Representing communication,  compassion, and competence in the era of AI. AMA J. Ethics 21, E1009–E1013.  doi: 10.1001/amajethics.2019.1009 | IV- Commentary | Ethical questions relating to quality of care and patient-clinician relationships | - Explains that artificial intelligence (AI) is now integrated into a variety of fields, including medicine. AI applications raise numerous ethical questions, particularly about quality of care and patient-clinician relationships. This article accompanies 2 digital photo-paintings that address these ideas narratively and visually, with special emphasis on communication, compassion, and competence. | United States |
| **Yang, et al., 2022** | Yang, H. C., Rahmanti, A. R., Huang, C. W., and Li, Y. J. (2022). How can  research on artificial empathy be enhanced by applying deepfakes? J. Med. Int.  Res. 24:e29506. doi: 10.2196/29506 | VI- Design study | Using an open data set of doctor-patient interactions to develop artificial empathy based on facial emotion recognition | - Facial emotion recognition allows a doctor to analyse patients' emotions, so that they can reach out to their patients through empathic care. However, face recognition data sets are often difficult to acquire; many researchers struggle with small samples of face recognition data sets. Further, sharing medical images or videos has not been possible, as this approach may violate patient privacy. The use of deepfake technology is a promising approach to deidentifying video recordings of patients' clinical encounters. Such technology can revolutionize the implementation of facial emotion recognition by replacing a patient's face in an image or video with an unrecognizable face-one with a facial expression that is similar to that of the original. - This technology will further enhance the potential use of artificial empathy in helping doctors provide empathic care to achieve good doctor-patient therapeutic relationships, and this may result in better patient satisfaction and adherence to treatment. | Taiwan |
| **Yao et al., 2020** | Yao, H., de Siqueira, A. G., Foster, A., Galynker, I., and Lok, B. C. (2020).  “Toward automated evaluation of empathetic responses in virtual human  interaction systems for mental health scenarios,” in Proceedings of the 20th ACM  international conference on intelligent virtual agents. | V- Conference paper | Automated evaluation of empathetic responses in virtual human interaction systems for mental health scenarios | - This paper investigates the process of automating the evaluation of empathetic response levels in virtual human interaction systems implementing mental health scenarios. Two suicidal virtual patients were developed to collect clinician participants' empathetic responses. - Tested the virtual human interaction with healthcare trainees. Trainees' empathetic responses were evaluated by experts to use the ECCS scale based on the ECCS level (Empathetic Communication Coding System). Clinician participants' empathetic responses to virtual patients were evaluated by experts and the classifiers. The performance of the classifiers was evaluated using the experts' coded level of clinicians' empathetic responses as a test dataset. - This work demonstrates the applicability of using virtual agents techniques to identify empathy levels of clinicians' responses automatically. This work shows the potential of using virtual human interaction to train clinicians' skills to show empathy. Feedback could be provided to clinicians based on the evaluation results. | United States |
| **Yokoo, et al, 2020** | Yokoo, K., Atsumi, M., Tanaka, K., Wang, H., and Meng, L. (2020). “Deep learning based emotion recognition iot system,” in Proceeding of the 2020 international conference on advanced mechatronic systems (ICAMechS), 203–207.  doi: 10.1109/ICAMechS49982.2020.9310135 | VI- Design study | Deep learning based emotion recognition internet of things system | - IoT systems are wildly used in various fields and received the most attention in healthcare research. This study aims to realize an emotion recognition IoT system for helping check the people feeling and health status. In edge side, the designed system applies a motion sensor, and a RGB camera which were equipped on a compact edge device, Raspberry Pi, for detecting human motion and taking people’s images. - When the motion sensor detects motion, the RGB camera is started and an image of the person is taken. Then, the face area in the image are cropped from the image by Haar feature-based cascade classifiers. When the face is detected, deep learning model of MobileNet is applied for human identification, and the face image is sent to a server for emotion recognition by deep learning. - In terms of human identification models, MobileNet, which was equipped on the Raspberry Pi, has been trained previously on a GPU machine. An emotion recognition deep learning model is also trained by GPU machine and equipped on a server machine. Mobile Net is also selected as emotion recognition deep learning model, according to the compassion of nine state-of-the-art deep learning models. - In the experimental results of this technology, the motion sensor and RGB camera work well, and the human identification accuracy almost achieves 100%. Furthermore, face images are transferred from edge to cloud correctly, and emotion recognition also achieves a better accuracy. | Japan |
| **Yun et al., 2021** | Yun, J. H., Lee, E., and Kim, D. H. (2021). Behavioral and neural evidence on consumer responses to human doctors and medical artificial intelligence. Psychol.  Mark. 38, 610–625. | VI- Design study | Study to explore whether consumers will accept artificial intelligence (AI) as a medical care provider | - Using evolution theory to investigate the implicit psychological mechanisms that underlie consumers' interactions with medical AI and a human doctor. In a Behavioral investigation (Study 1), consumers expressed a positive intention to use medical AI's healthcare services when it used personalized rather than mechanical conversation. However, neural investigation (Study 2) using functional magnetic resonance imaging revealed that some consumers' implicit attitudes toward medical AI differed from their expressed behavioural intentions. The brain areas linked with implicitly apathetic emotions were activated even when medical AI used a personalized conversation, whereas consumers' brains were activated in areas associated with prosociality when they interacted with a human doctor who used a personalized conversation. On the basis of this neural evidence, consumers perceive an identical personalized conversation differently when it is offered by a medical AI versus a human doctor. These findings have implications for the area of human-AI interactions and medical decision-making and suggest that replacing human doctors with medical AI is still an unrealistic proposition. | Korea |
| **Zelmer et al., 2018** | Zelmer, J., van Hoof, K., Notarianni, M., van Mierlo, T., Schellenberg, M., and  Tannenbaum, C. (2018). An assessment framework for e-mental health apps in  canada: results of a modified delphi process. JMIR mHealth uHealth 6:e10016.  doi: 10.2196/10016 | II- Research study | Describes an attempt by Canadian stakeholders to develop an e-mental health assessment framework that responds to the unique needs of people living in Canada in an evidence-based manner | - Consensus was reached on 9 guiding principles: evidence based, gender responsive, culturally appropriate, user centered, risk based, internationally aligned, enabling innovation, transparent and fair, and based on ethical norms. In addition, 15 informative and evaluative criteria were defined to assess the effectiveness, functionality, clinical applicability, interoperability, usability, transparency regarding security and privacy, security or privacy standards, supported platforms, targeted users, developers' transparency, funding transparency, price, user desirability, user inclusion, and meaningful inclusion of a diverse range of communities. | Canada |
| **Zhang et al., 2021** | Zhang, Z., Citardi, D., Wang, D., Genc, Y., Shan, J., and Fan, X. (2021). Patients’  perceptions of using artificial intelligence (AI)-based technology to comprehend  radiology imaging data. Health Inform. J. 27:14604582211011215. | II- Qualitative study | Study is to understand patients' perceptions and acceptance of using AI technology to interpret their radiology reports | - Results of radiology imaging studies are not typically comprehensible to patients. With the advances in artificial intelligence (AI) technology in recent years, it is expected that AI technology can aid patients' understanding of radiology imaging data. Interviews with 13 participants to elicit reflections pertaining to the use of AI technology in radiology report interpretation. - Participants have a generally positive attitude toward using AI-based systems to comprehend their radiology reports. AI is perceived to be particularly useful in seeking actionable information, confirming the doctor's opinions, and preparing for the consultation. However, various concerns related to the use of AI in this context, such as cyber-security, accuracy, and lack of empathy. - Highlights the necessity of providing AI explanations to promote people's trust and acceptance of AI. Designers of patient-centered AI systems should employ user-centered design approaches to address patients' concerns. Such systems should also be designed to promote trust and deliver concerning health results in an empathetic manner to optimize the user experience. | United States |
| **Zheng et al., 2022** | Zheng, F., Zheng, Y., Liu, S., Yang, J., Xiao, W., Xiao, W., et al. (2022). The effect  of m-health-based core stability exercise combined with self-compassion training  for patients with nonspecific chronic low back pain: a randomized controlled pilot  study. Pain Ther. 11, 511–528. doi: 10.1007/s40122-022-00358-0 | II- Research study | Study of the effect of M-Health-Based core stability exercise combined with self-compassion training for patients with back pain | - Helping patients self-manage low back pain through a biological-psycho-social model using mindfulness-related therapy (self-compassion training) and mobile health technology. The results showed that the efficacy of the combined group seemed to be more obvious and worthy of further study. - Results indicate that participants in the group of m-health-based core stability exercise combined with self-compassion training may experience faster relief from pain intensity and back disability than those in the group of m-health-based CSE alone. | China |
